# Charting net-zero pathways for ASEAN's energy sector

**DOI:** 10.1093/pnasnexus/pgaf389

**Published:** 2026-01-06

**Authors:** Sheng Zhong, Bin Su, Dimitri Papageorgiou, Fu Sau Yeung, Tsan Sheng Ng, Saifudin Abubakar

**Affiliations:** Energy Studies Institute, National University of Singapore, Singapore 119620, Singapore; Energy Studies Institute, National University of Singapore, Singapore 119620, Singapore; Department of Industrial Systems Engineering and Management, National University of Singapore, Singapore 117576, Singapore; ExxonMobil Technology and Engineering Company, Annandale, NJ 08801, USA; Energy Studies Institute, National University of Singapore, Singapore 119620, Singapore; Department of Industrial Systems Engineering and Management, National University of Singapore, Singapore 117576, Singapore; ExxonMobil Asia Pacific, Singapore 098633, Singapore

**Keywords:** electricity, hydrogen, net zero, energy systems modeling, ASEAN

## Abstract

The Association of Southeast Asian Nations (ASEAN) is at a turning point to drive an energy transition toward a low-carbon future. Investigating ASEAN's decarbonization strategies is timely. We present a capacity expansion model with hourly resolution for ASEAN to meet net-zero emissions by 2050, integrating electricity generation and hydrogen production. The results show two “bookend” pathways. ASEAN can decarbonize its power sector through an accelerated expansion in renewables and battery storage (up to 95% and battery charge up to 28% in 2050) or an expansion in carbon capture and storage (CCS) and hydrogen (up to 46 and 15%, respectively). CCS is found to play a key role in hydrogen production. For power system operation, grid connectivity can lower battery storage demand and power reserves but requires higher power system flexibility. Our findings can help decision-makers identify the roles of key decarbonization strategies in ASEAN and navigate between various scenarios.

Significance Statement Southeast Asia faces rapidly growing electricity and hydrogen demand alongside urgent decarbonization pressures. This study develops a unified hourly Association of Southeast Asian Nations (ASEAN)-wide energy system model that co-optimizes electricity generation, cross-border transmission, and hydrogen production. We show how different combinations of renewables, grid interconnection, storage, carbon capture and storage, and hydrogen strategies shape the region's net-zero pathways. Grid connectivity reduces reserve and storage needs while increasing operational flexibility requirements. These findings offer policymakers a clear, evidence-based foundation for assessing technological trade-offs and strengthening regional cooperation and long-term planning toward ASEAN's net-zero goals.

## Introduction

**Fig. 1.**
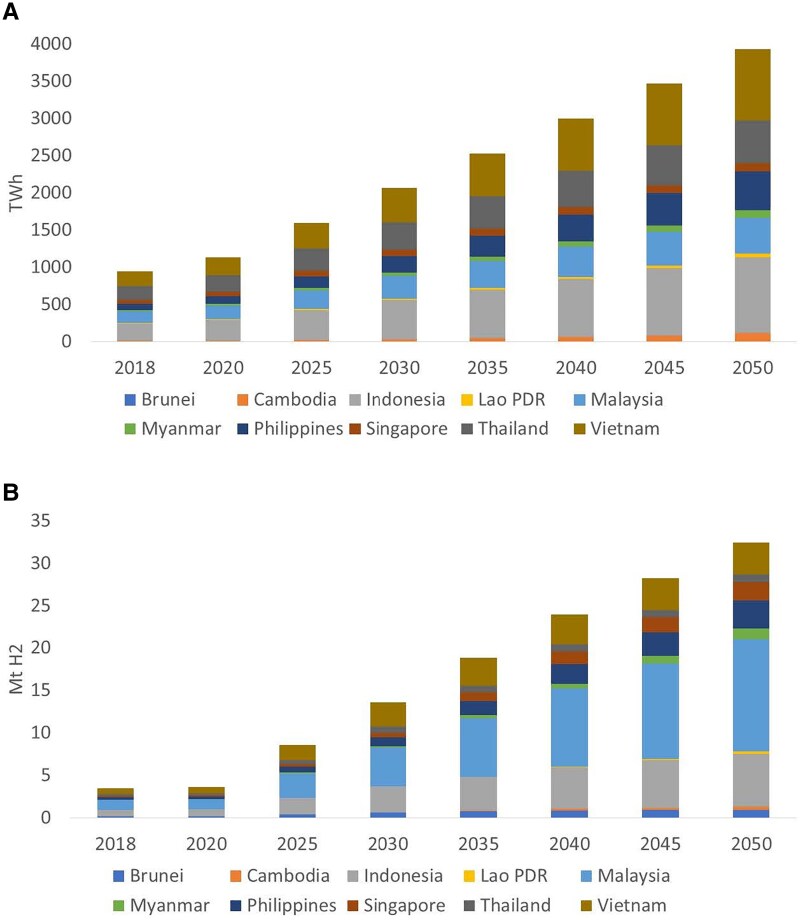
Projections of electricity and hydrogen demand of ASEAN. A) Electricity demand by ASEAN country. B) Hydrogen demand by ASEAN country. See Supporting Information for details.

The Association of Southeast Asian Nations (ASEAN) is one of the fastest-growing regions in the world. The socioeconomic prospects for this region, particularly the ongoing industrialization (1, 2), are perceived to drive the energy demand to grow substantially. Electricity is expected to account for up to 60% of the region's energy supply structure (3), and the electricity demand is projected to more than triple by 2050 from the 2018 level (Fig. 1A). As an emerging energy option, hydrogen demand in this region would increase by more than eight times by 2050 (Fig. 1B). However, ASEAN's power sector still heavily relies on conventional fossil fuels (>73% of the current electricity generation mix) (4), and renewable energy, except for hydropower, is underutilized (2). With ASEAN's emissions growing faster than in other regions (5), investigating ASEAN's decarbonization strategies and the trade-offs is timely.

All ASEAN countries have announced their Nationally Determined Contribution (NDC) targets for 2030 (6). Beyond the NDCs, countries like Brunei, Indonesia, Malaysia, Singapore, Thailand, and Vietnam further plan to achieve net-zero emissions by 2050 or later (3). Key strategies have been identified (7), i.e. renewable energy, cross-border transmission, and emerging low-carbon technologies such as carbon capture and storage (CCS) and hydrogen. For decision-makers, it is crucial to understand the roles of the key strategies in net-zero pathways.

The identified key strategies are only implementable under certain conditions and face various challenges and barriers. In literature, however, those strategies are often addressed in isolation and may not capture the trade-offs among them. Here, we present an energy system optimization model to analyze the pathways to net-zero emissions by 2050 for ASEAN's electricity and hydrogen sectors by integrating all key strategies in a unified framework. Extending the URBS modeling tool (8, 9), this study incorporates hourly resolution for the power sector (Fig. S1) and includes an additional hydrogen production sector.

Across prior studies on the net-zero pathways for ASEAN's energy sector (9–12) and the whole economy (11, 13), a pervasive finding is an aggressive transition toward renewable energy (particularly solar and wind). However, to enhance renewable utilization, ASEAN faces critical institutional barriers, such as limited energy governance reform and capability for renewable energy (14), complicated regulatory and business environments (14, 15), and inefficient market mechanisms (16). Climate change can lead to water constraints for hydropower (17, 18), and the portfolios of hydro dams need to be carefully designed to mitigate the adverse environmental impacts (19). Geothermal resources are limited and mostly available in Indonesia and the Philippines (13). There are also important differences between the technical and achievable potential for renewables, suggesting less favorable situations where renewable potentials cannot be fully utilized (Table S3).

Cross-border transmission through the ASEAN Power Grid (APG) is an important enabler for the energy transition in ASEAN. As renewable resources are unevenly distributed across ASEAN countries, grid connectivity can reallocate renewable energy to the load centers (13) and enhance the utilization of solar (19). Enhanced grid connectivity can reduce energy system costs (9) and improve energy access (20). The APG's growth, however, has been slow due to institutional and technical barriers, such as cross-border policy coordination, information sharing among system operators, and benefit sharing (20, 21). The impacts of the APG on daily power system operations in this region remain underexplored.

CCS and hydrogen are emerging technologies that are perceived to unlock greater decarbonization potential (22, 23). In particular, the International Energy Agency (IEA) (3, 24) hypothesizes that ASEAN's hydrogen demand can increase by 823% by 2050 from the 2018 level (Fig. 1B). There are indeed opportunities for ASEAN to benefit from the applications of CCS (25, 26) and hydrogen (27, 28). The Government of Singapore has already worked with major industry partners such as ExxonMobil and Shell to develop cross-border CCS projects (29) and developed a national strategy to support hydrogen-based energy use for electricity generation and maritime bunkering (30). However, there are also critical barriers, such as the lack of a regulatory framework for CCS and hydrogen markets, cross-border governance coordination, storage security, and challenges due to high costs (26, 28). The hydrogen sector and its connection with the power sector are rarely modeled in the prior studies on ASEAN's net-zero pathways.

The literature on ASEAN's net-zero transition incorporating a broad set of decarbonization strategies remains sketchy (9). A strand of literature has applied approaches such as policy review (31, 32), technology screening (33), life cycle analysis (34), composite index (35), and econometric analysis (36), which cannot provide cost-optimal net-zero transition pathways. Energy system models for ASEAN have investigated cost-optimal power generation portfolios. However, some of the models have only focused on parts of the decarbonization strategies, for example, the pathways for achieving certain renewable targets (e.g. 100% renewables) (12, 13, 37), wind power utilization (38), solar and transmission in the Lower Mekong region (19), and technological options for transmission (39). To support interregional power system planning in ASEAN, Chang et al. (40), Chang and Li (41) propose the dynamic linear programming models, and Kang et al. (42) develop a two-level stochastic-robust optimization model under uncertainty, but those models are at the annual level through 2040 without considering hydrogen. Using the Low Emissions Analysis Platform, the optimization approach has been developed to analyze the net-zero power sector pathways for the whole ASEAN (10, 43) and selected ASEAN countries (44), while the cross-border transmission is not explicitly modeled. The URBS model in Huber et al. (45) is an optimization model incorporating transmission with hourly and subnational resolutions, but country-level climate targets and hydrogen are not included.

This paper seeks to fill the knowledge gap regarding how those key decarbonization strategies can shape the net-zero pathways for ASEAN's energy sector through 2050. Using the URBS modeling tool (8), we minimize total energy system cost for production, transmission, and storage over 2018–2050. The contributions include (i) developing a power capacity expansion model with hourly resolution for ASEAN to capture the differences in intermittent renewables and electricity load, (ii) finding cost-optimal pathways for ASEAN's electricity and hydrogen sectors to achieve net zero by 2050, (iii) assessing the use of hydrogen and the trade-offs between hydrogen and other decarbonization options in ASEAN's power sector, and (iv) quantifying the impacts of cross-border electricity transmission on power system operation in ASEAN.

## Results

### Power generation and hydrogen production

Four main scenarios have been implemented. To design those scenarios, we consider two cases regarding the status of grid interconnections and the extent to which renewable resources can be utilized, i.e. moderate or high levels (Table 1). Different combinations of those cases give four scenarios. Across all scenarios, hydrogen import from outside ASEAN (i.e. the Australian Renewable Energy Hub) is allowed (27), and the restrictions on electricity imports apply. In all scenarios, all countries are required to meet their unconditional NDC targets for 2030 and achieve net-zero emissions by 2050.

**Table 1.** Scenario design.

Area of focus	Status
Grid interconnection	“No APG”: cross-border electricity transmission is prohibited, and thus, countries rely on their resources to meet domestic electricity demand.
	“Full APG”: countries can trade electricity, and thus, expansion in electricity transmission is allowed from the initial level.
Renewable potentials	Moderate renewable resource potentials.
	High renewable resource potentials for hydro, solar, onshore wind, and offshore wind.

Electricity transmission network and country-specific electricity import constraints are available in Fig. S3. Moderate renewable resource potentials are primarily based on those released in the policy documents and are lower than the technical potentials (used in the high potential case). See Table S3 for detailed discussions on the moderate and high renewable resource potentials.

**Fig. 2.**
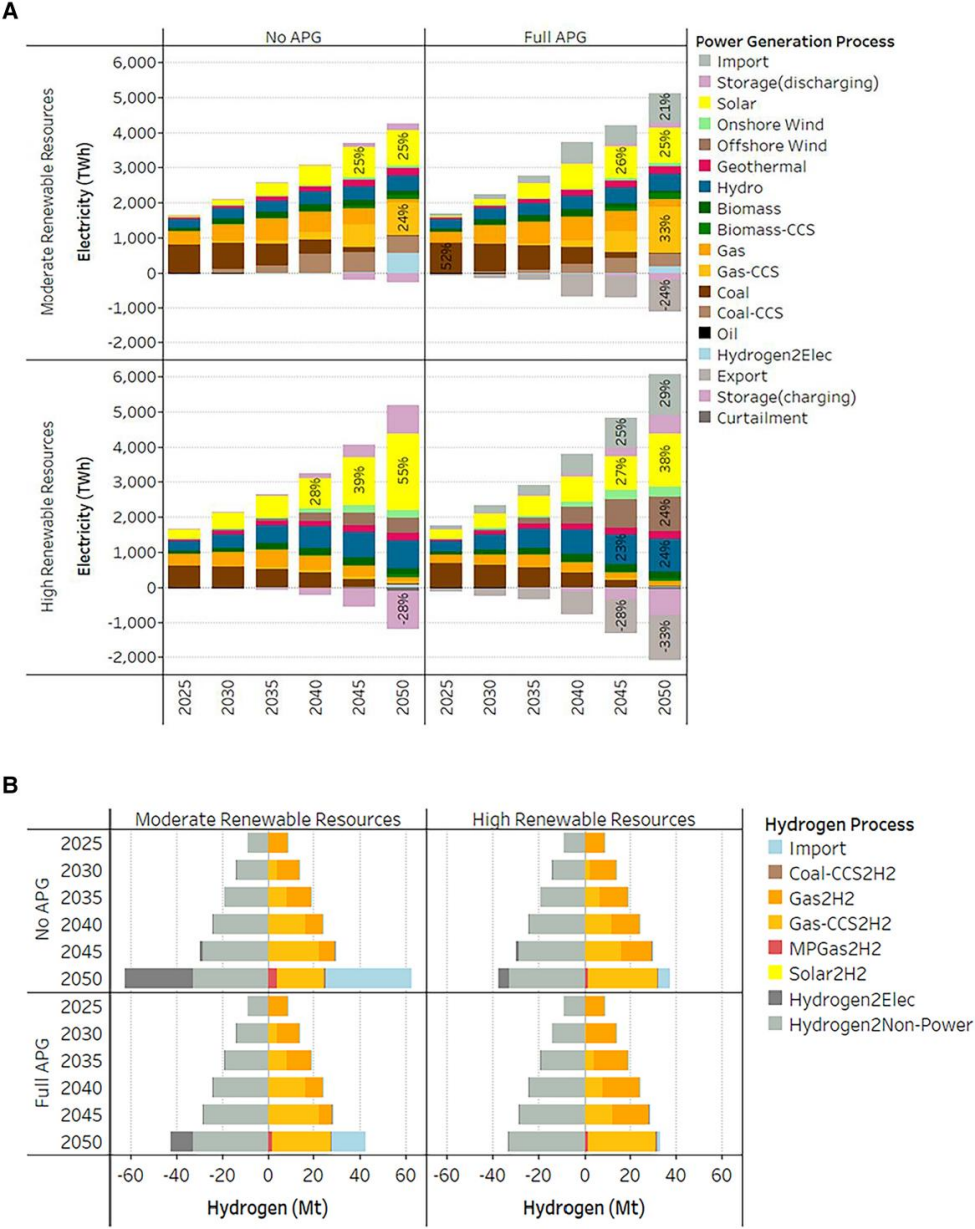
Projected electricity generation and hydrogen production in ASEAN. A) Projected annual electricity generation mix. B) Projected annual hydrogen production and consumption mix. We present the ASEAN electricity generation mix (sum over all individual countries). ASEAN's hourly electricity generation mix is available in Fig. S7, and country-specific annual generation results are presented in Fig. S8. In B), a negative value on the *x* axis indicates hydrogen consumption. Hydrogen production includes domestic production and imports from outside ASEAN. Australia (i.e. Australian Renewable Energy Hub) is a major low-carbon hydrogen sourcing country due to the country's significant wind and solar resources and the policy support (27).

We first present an overview of the electricity generation and hydrogen production results from the four main scenarios. Figure 2A shows ASEAN's optimal electricity generation mix by summing the hourly electricity generation across all countries. Depending on the availability of renewable resources, ASEAN's power sector will follow two very different trajectories.

With moderate renewable resources, the expansion of renewables is restricted, and low-carbon alternatives can play an important role. ASEAN generation mix still has a significant portion of fossil fuel-based electricity (mostly those with CCS), which consists of up to 39% of the mix without APG and 48% of that with full APG in 2050. However, there are two critical transitions in the net-zero pathways, i.e. the switch from coal to gas, followed by further decarbonization efforts that rely on emerging low-carbon alternatives such as CCS and hydrogen. Without APG, 39 and 15% of the electricity generated will be from CCS processes and hydrogen in 2050, respectively. In such a case, hydrogen would be needed in countries like Brunei, Singapore, and Vietnam (Fig. S8). With full APG, however, CCS would account for 46% of 2050 electricity generation, and the share of hydrogen drops to 5%.

With high renewable resources, the portion of fossil fuels in the ASEAN generation mix is reduced from ∼58% in 2025 to ∼5% in 2050. The increasing electricity demand is satisfied by the rapid expansion of renewable energies, especially solar and wind. Without APG, the penetration of solar energy would increase significantly in the 2050 electricity generation mix (reaching 55%), followed by hydro and offshore and onshore wind (19, 11, and 6%, respectively). With full APG, renewable energy still plays a major role, accounting for a larger share than that without APG in the 2050 generation mix. With transmission lines available to deliver electricity between ASEAN countries, the shares of offshore wind and hydro are both increased to 24% of the mix. These two sources substituted the heavy dependence on solar in the no APG scenarios thanks to the connections of Vietnam with rich offshore wind resources and the Mekong River region with excess hydro energy.

Further, Fig. 2B presents ASEAN's domestic hydrogen production and consumption mix. To meet increasing hydrogen demand, in all the scenarios, domestic hydrogen production is mainly based on steam methane reforming with natural gas as the feedstock. Low-carbon transition will take place to achieve net-zero emissions by 2050. CCS is projected to penetrate deeply into the gas-to-hydrogen processing plants. In all the scenario projections, all the gas-to-hydrogen production capacities will be with CCS. On top of that, methane pyrolysis will start to develop in the 2050 as it is emission free. However, the share of production is relatively low, with 6% in the most extreme scenario (moderate renewable resources and no APG) and ∼4% in other scenarios.

Consistent with Fig. 2A, the demand for hydrogen in the power sector is high when the regional power grid connection is limited and renewable potentials are moderate. The induced high demand for hydrogen will need to be supplied by hydrogen imports from outside ASEAN in 2050. In the most extreme scenario (moderate renewable resources and no APG), ∼60% of the supply will be the low-carbon hydrogen import from Australia (e.g. Brunei, Singapore, and Vietnam; see Fig. S8).

In addition to the four main scenarios, we have implemented a series of sensitivity tests by changing key model parameters (i.e. coal price, natural gas price, battery storage cost, variable renewable energy (VRE) cost, CCS cost, transmission line cost, electricity demand, and hydrogen demand). Overall, the two “bookend” pathways remain consistent across all sensitivity tests (see detailed results in Figs. S9–S18).

### Economic incentivize for hydrogen to be viable in electricity generation

As shown in Fig. 2A, hydrogen, as an emerging technological option, is a must-have component for the power sector to achieve net-zero targets in less favorable situations in which renewable resource potentials and grid connectivity are restricted. Due to the high-cost trajectories in the current main scenarios, hydrogen will only be an important part in electricity generation in 2050, and there are indeed some trade-offs, i.e. substitution of hydrogen in domestic electricity generation by electricity imports and renewables. This raises an obvious follow-up question about the extent to which cost reductions are needed for hydrogen to be viable in electricity generation.

To do so, we implement several alternative scenarios of cost reductions for hydrogen on the supply side (i.e. hydrogen production and import). This is done by modifying the two main scenarios in which renewable resource potentials are moderate. We reduce the CAPEX as well as the fixed cost of those low-carbon hydrogen production technologies from 2030 onwards by 60, 70, and 80%. Such technologies include solar-to-H_2_, wind-to-H_2_, gas-to-H_2_-CCS, coal-to-H_2_-CCS, and methane pyrolysis-to-H_2_, given their low-emission factors. The same cost reductions (in terms of percentage) are applied to the hydrogen imports from outside ASEAN as well. The cost reduction design seeks to provide a comparable case to the stated policy target (e.g. 80% cost reduction in the US Hydrogen Shot). We have also tested the scenarios with smaller cost reductions (10–50%), but the results do not greatly differ from those of the main scenarios.

**Fig. 3.**
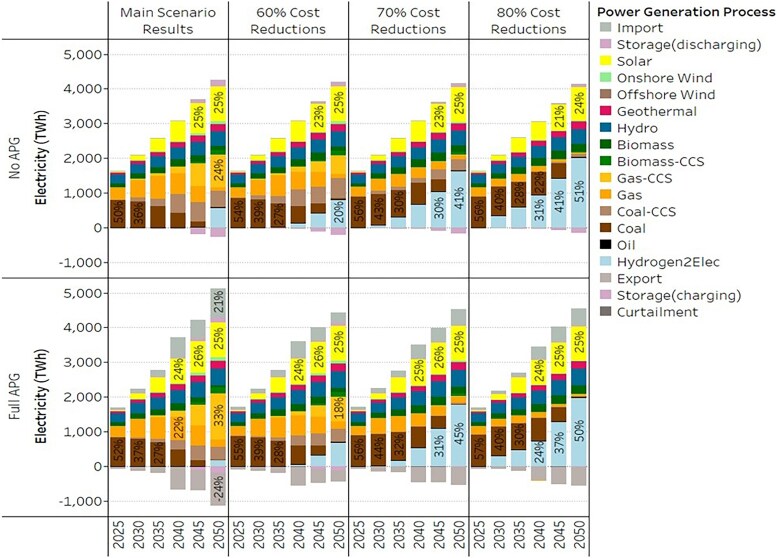
Effects of hydrogen cost reductions on power generation mix in ASEAN. We present ASEAN's electricity generation as a function of cost reductions in hydrogen production and imports. In this figure, moderate renewable resource potentials are considered.

Figure 3 illustrates the electricity generation mix results of those alternative scenarios. With increasing cost reductions, hydrogen as a fuel can be adopted in electricity generation earlier and at a larger scale (up to 51% in 2050). With a 70% cost reduction in CAPEX and fixed cost of hydrogen production and hydrogen import, hydrogen can contribute to electricity generation (in a small share) as early as 2030. This, for example, requires a 77% decrease in hydrogen import price, i.e. from USD 6.91/kg H_2_ in 2020 to about USD 1.56/kg H_2_ in 2030. Such a cost reduction is in line with the stated hydrogen price targets, e.g. the US Hydrogen Shot target of reducing the cost of clean hydrogen by 80% to USD 1/kg H_2_ in one decade (46). The alternative scenarios shown here indicate that substantial economic incentives, e.g. research and development (R&D) funding support and tax credits, are needed for the adoption of hydrogen in electricity generation.

### Effects of renewable resources and grid connectivity on energy storage

**Fig. 4.**
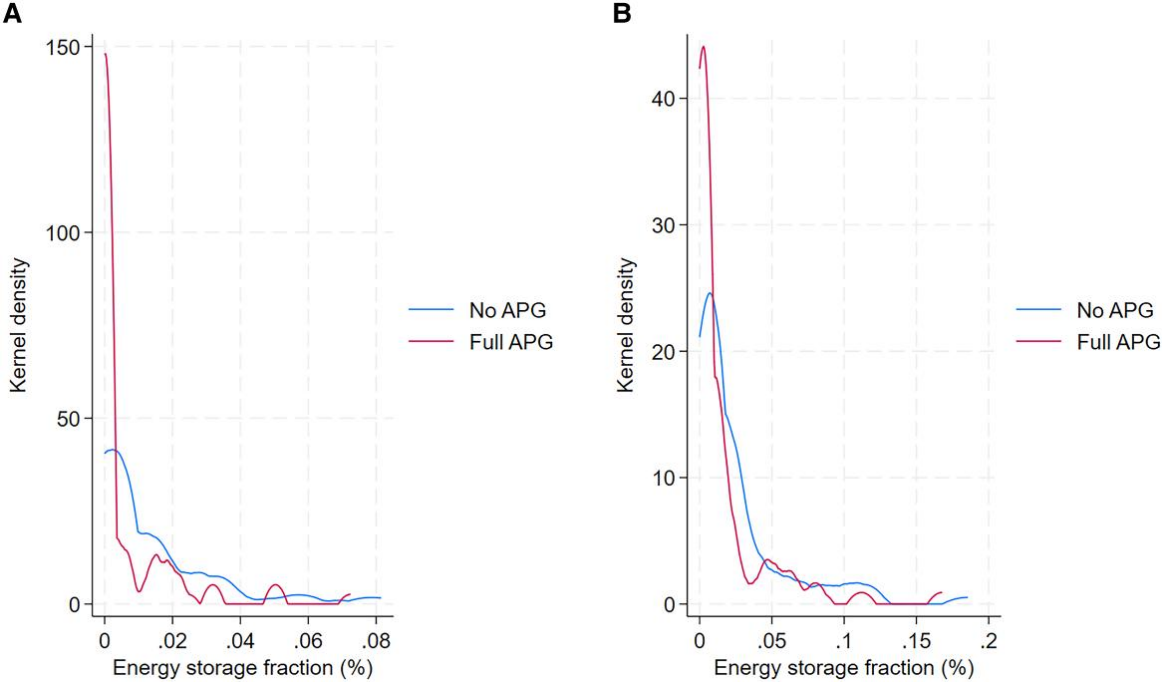
Distribution of energy storage fraction by scenario. A) Moderate renewable resource. B) High renewable resources. In both panels, each distribution curve represents a scenario and is plotted by covering all country–year pairs. We estimate the kernel density using the Epanechnikov kernel. Energy storage fraction is measured in battery capacity (in GWh) relative to annual domestic electricity demand (in GWh) (47).

Higher utilization of renewable energy, particularly VRE, needs to be supported by enhanced energy storage. In Fig. 4, we plot the distributions of energy storage fraction (as measured by battery storage capacity relative to annual electricity demand) (47) for the main scenarios, covering all countries and modeling years. With moderate renewable resources in Fig. 4A, the average VRE in electricity generation of the sample is about 14%, whereas the sample's average VRE share increases to 25% with high renewable resources. In both panels, most country–year pairs have zero or nearly zero energy storage fraction, as the distribution curves peak around the line x=0. This is because in the early years with low VRE in electricity generation, there is little demand for battery storage. In Fig. 4B, more country–year pairs are distributed in the range with an energy storage fraction >0.08% (the maximum energy storage fraction in Fig. 4A). In addition, the density of country–year pairs with a small energy storage fraction (zero or nearly zero) is much lower in Fig. 4B than in Fig. 4A. The average energy storage fraction is 0.009% in Fig. 4A and 0.02% in Fig. 4B.

Further, cross-border transmission can significantly lower the demand for domestic energy storage. In both panels of Fig. 4, an important pattern is that the curve with full APG is distributed to the left of the curve without APG, meaning that the energy storage fractions with full APG are generally smaller than those in no APG scenarios. There are more country–year pairs with zero (or nearly zero) energy storage fraction in scenarios with full APG, as the peak of the full APG curve is above the curve without APG. In Fig. 4A, the average energy storage fraction drops from 0.012 to 0.006% if APG is allowed, and from 0.02 to 0.01% in Fig. 4B.

### Lower power system reserve due to cross-border transmission

**Fig. 5.**
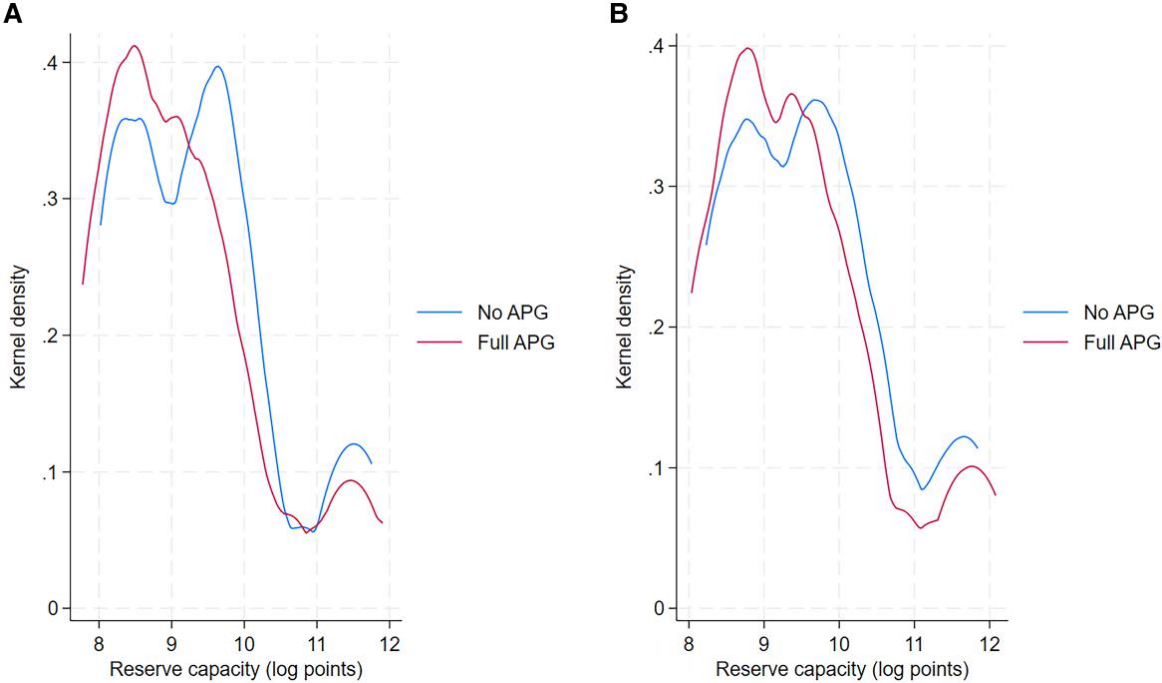
Distribution of reserve capacity by scenario. A) Minimum daily reserve. B) Maximum daily reserve. In both panels, each distribution curve is plotted by covering all pairs of day–year for the scenarios with (or without) APG for ASEAN. We estimate the kernel density using the Epanechnikov kernel. The *x* axis is rescaled in log points. A value of zero on the *x* axis means a power reserve level of 1 MWh.

Further, we investigate the effects of grid interconnectivity on power system reserve. To do so, we calculate the minimum and the maximum reserve by day–year scenario for the entire ASEAN power system. Figure 5 depicts the distributions of the two reserve indicators by distinguishing between the scenarios with and without APG. In both panels, the distribution curve for scenarios with full APG is located below and to the left side of the curve for those scenarios without APG. This means that cross-border transmission can overall reduce the minimum and maximum power reserve for the whole power system on a daily basis. For power system operators and planners, the implication is that reliable cross-border transmission can be beneficial by substituting excess domestic electricity generation capacity.

### Higher power system flexibility due to cross-border transmission

**Fig. 6.**
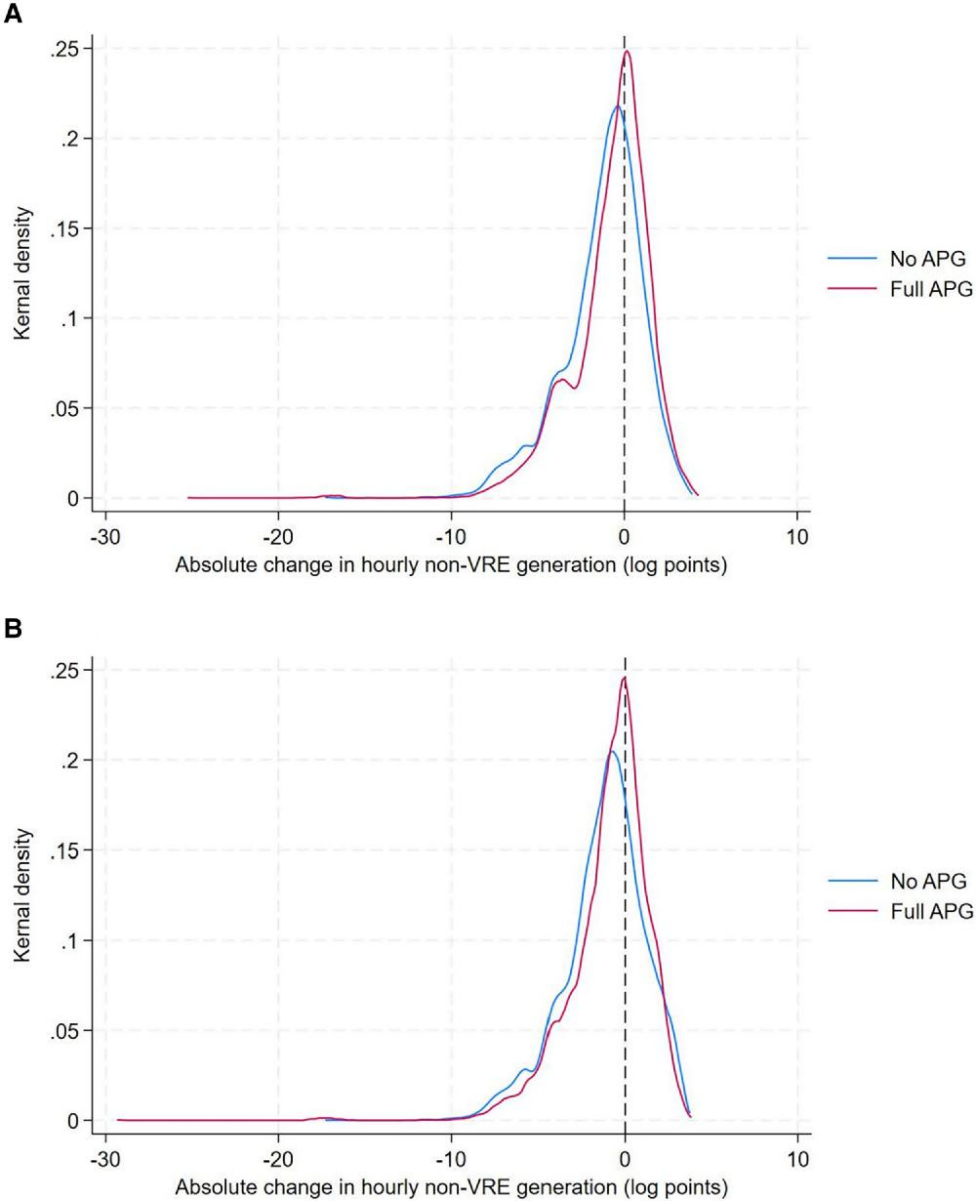
Distribution of absolute change in hourly non-VRE generation by scenario. A) Moderate renewable resources. B) High renewable resources. In both panels, each distribution curve represents a scenario and is plotted by covering all pairs of country–hour–day–year. We estimate the kernel density using the Epanechnikov kernel. The *x* axis is rescaled in log points. A value of zero on the *x* axis means that the absolute change in hourly non-VRE generation is 1 GWh. A negative value on the *x* axis indicates that such a change is <1 GWh.

Enhanced grid connectivity, however, would require a higher requirement for power system flexibility. We calculate the absolute change in non-VRE electricity generation by hour, day, year, country, and scenario. Figure 6 illustrates the distributions of this indicator. Unlike Figs. 4 and 5, the distribution curves for the scenarios without APG are located to the left of the curves for those with full APG in Fig. 6. Also, the peak of the full APG curve is above that of the no APG curve in both panels. This means that in those scenarios with full APG, a larger scale of hourly ramping-up (or ramping-down) of non-VRE electricity generation would be needed. An explanation is that with cross-border transmission, the countries with higher VRE resource potentials can further extend their VRE generation and export to the load centers where renewable resources are limited. Thus, VRE (e.g. solar) can account for a larger share in hourly electricity generation with full APG (e.g. during the hours with high solar capacity factors) and drop to zero in electricity generation (e.g. solar generation during night hours). With greater fluctuations in VRE generation, the non-VRE electricity generation is therefore required to be more flexible to meet system demand on an hourly basis. The development of full APG will bring various regional generation and transmission system operators into an integrated electricity market. This will require better coordination and information sharing between system operators and harmonization of the ancillary services control mechanisms.

## Discussion

This study explores how the interconnectivity of the regional power grid and the integration of renewable energy interact with low-carbon alternatives, including CCS and hydrogen applications, to achieve net zero in 2050. Four different scenarios are considered. Two main net-zero pathways are suggested in the power sector: renewable energy supported by storage and emerging low-carbon alternatives (CCS and hydrogen).

We identify several key findings. First, for the supply of hydrogen, gas-to-hydrogen with CCS will be the key technology for domestic hydrogen production. Low-carbon hydrogen imports from outside of ASEAN will support a more rapid expansion of hydrogen for the power sector. Moreover, we quantify the extent of CAPEX cost reductions needed for the early adoption of hydrogen in ASEAN's power sector. If renewable energy potential is limited or cross-border transmission capacity is constrained, hydrogen will be a necessary part of daily operations in the long run. For early adoption of hydrogen in the power sector, however, substantial cost reductions are needed.

Second, for the pathways with high expansion of VRE and cross-border transmission, non-VRE operations will need to be more flexible to support intermittency. Higher VRE penetration will result in a higher reserve capacity. However, a high extent of cross-border connectivity will reduce the reserve capacity and battery storage capacity of the system. Future studies can consider more cost-competitive energy storage options to facilitate the expansion of VRE, e.g. pumped hydroelectricity storage.

The two different pathways give very different policy implications for the ASEAN member states. First, a more interconnected power grid can reduce the expensive infrastructure investment costs and improve utilization of renewable resources. The integration of fragmented regional electricity markets is needed to unlock the full benefits of APG. This requires more coordinated energy infrastructure planning (e.g. generation, transmission, and power reserve), enhanced information sharing among system operators, and harmonized ancillary services control mechanisms across ASEAN member countries. Proper pricing design for electricity in APG can provide economic incentives and facilitate benefit sharing. Transparent political commitment can also improve the confidence of state governments and investors to take part in APG projects.

Second, for the pathways using emerging low-carbon alternatives, strategies to reduce CCS and hydrogen production costs and support deployment need to be explored. Both technologies face significant uncertainties and risks that may hinder their large-scale deployment, such as technological choice, storage security, policy support, carbon pricing, and public acceptance. For CCS, ASEAN countries need to identify suitable carbon storage sites and develop international regulatory frameworks to manage potential leakage and long-term liability. The cost of CO_2_ transport and storage in ASEAN can be much higher than the assumed cost, resulting in potential trade-offs between CCS and other low-carbon generation (e.g. renewables and hydrogen). For hydrogen, the development of the entire value chain for production, transport, storage, and trading market will be needed for rapid hydrogen-to-electricity process expansion. Market development in various downstream sectors, for example, the hydrogen-based bunkering fuel transition for international shipping and port operations, can further catalyze the development of a hydrogen economy.

## Materials and methods

The viable pathway for electricity and hydrogen requires co-optimizing electricity dispatch and capacity expansion for both generation and transmission infrastructure. Our model builds upon an open-source model called URBS (8, 48), a linear programming optimization model focusing on power capacity expansion planning.

The URBS model considers multiple interconnected regions connected by a set of transmission lines. Each region (or each node in the transmission network) has a set of available generation processes that can be built at the beginning of each modeled year to meet hourly electricity demand while satisfying other constraints, such as the decarbonization targets and electricity import restrictions. The model determines the cost-optimal generation portfolio and transmission capacity for the whole system. This is done by minimizing the energy system costs (i.e. the objective function), comprising investment and fixed maintenance costs for generation InvG, energy storage InvS, and electricity transmission InvT, and those costs based on production levels, i.e. variable costs VarG and fuel costs FuelG:


(1) minCost=min(InvG+InvS+InvT+VarG+FuelG).

**Fig. 7.**
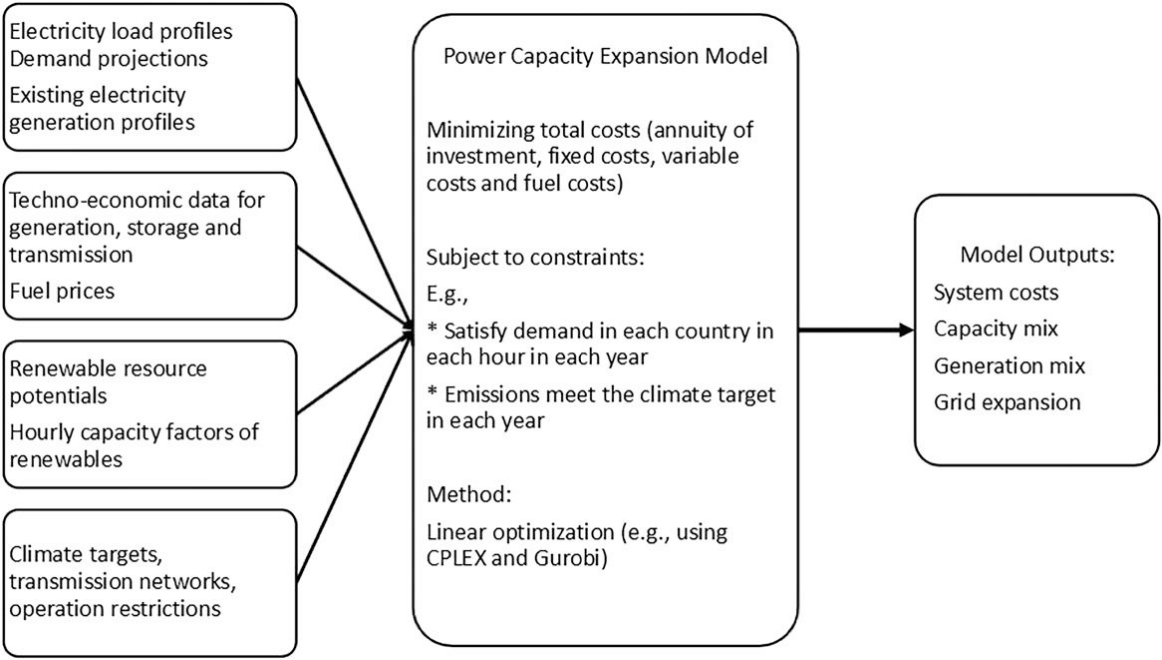
Stylized structure of the model. Detailed discussions on the model inputs are available in Supporting Information (Tables S1–S5 and Figs. S1–S6).

The cost minimization above requires a rich set of data inputs and is subject to various constraints. The complete mathematical description of the basic model, including the objective function and common model constraints, is available in the Supporting Information, which draws on the URBS model documentation (8, 48) and literature (9, 45). Figure 7 shows the basic model structure. The main constraint is that the electricity load in each time step, and each model region needs to be met through domestic generation, energy storage, and transmission. In this study, we also implement a constraint requiring each country to meet hydrogen demand annually (not hourly) through domestic hydrogen production and low-carbon hydrogen import from Australia. Other important constraints include the availability of renewable resources and emissions targets. The model will derive the country-specific generation portfolios for future expansions that can minimize the energy system costs while satisfying all constraints.

This model has been applied to study ASEAN countries in previous works, for example, by dividing the region into subnational areas (45, 49) or representing provinces as nodes (19). This study adopts a country-level representation in which each ASEAN country is viewed as a node in the transmission network. This allows better incorporation of national-level climate and energy policies into the model. Such a system-wide optimization has been implemented in the studies for ASEAN (13, 50). Due to data availability, the time span of the electricity load in the current study consists of 12 days for each modeling year, i.e. 12 × 24 = 288 h (Fig. S1). Each day represents a month of the year. We analyze the period 2018–2050. However, current time resolution may not fully capture the heterogeneity in electricity load patterns (e.g. weekdays and weekends, seasonal changes, etc.). A greater data collection effort is needed to enhance the time resolution in future studies.

We further extend the basic URBS model in several ways. First, we incorporate an additional type of industrial commodity $C_{\mathrm{ind}}$ into the model, with an annual demand constraint specified for each node and modeling year. This new commodity could potentially serve as input fuel in certain processes (e.g. hydrogen as a fuel in power generation), while also acting as the output product of other processes (e.g. domestic hydrogen production). Though an hourly load curve is not available for such an industrial commodity due to data availability, the net production in the energy system for each node must satisfy its annual external demand. Specifically, constraint (2) enforces that the commodity balance, denoted by CB(c,t), which is equivalent to total annual inputs minus the total outputs, must be less than or equal to the negative of annual demand from all external sectors represented by ${\underline{U}}_{c}$.


(2) $$\underset{t\in T_{m}}{\sum }\;CB(c,t)\leq -{\underline{U}}_{c},\;\;\forall c\in C_{\mathrm{ind}}$$

We have introduced a set of technologies for domestic hydrogen production (Table S1). With this new feature, the model can easily endogenously optimize the domestic hydrogen production portfolio and trade-offs across the multi-sector energy system simply by supplying relevant hydrogen demand and production technologies data. This includes accounting for the costs and emissions associated with hydrogen production together with the power sector. It is also capable of calculating the hydrogen demand from the power sector itself, allowing the model to assess questions about the use of hydrogen for electricity generation. However, hydrogen storage and transport between ASEAN countries are beyond the scope of the current paper. Hydrogen can be imported from outside ASEAN. In this study, the low-carbon hydrogen from the Australian Renewable Energy Hub is considered an import source.

Second, renewable resources, such as solar and onshore wind, can be used for both hydrogen production and different types of electricity generation processes, potentially competing for a shared renewable resource potential. To capture this competition, for each node with a set of available generation process p∈P, a list of process groups indexed by i∈I are added to the model. Each group consists of members $p\in P_{i}\subseteq P$. These processes (technologies) share resources or other attributes in common, e.g. solar and solar-to-H_2_, and onshore wind and wind-to-H_2_. Additional constraints are imposed for each group of processes relying on the same renewable potential *K*.


(3) $$\underset{\;p\in P_{i}}{\sum }\;\kappa_{p}\leq K_{i},\;\;\forall i\in I$$

To deal with the fluctuations in electricity demand and generator outages, we introduce power reserve constraints, requiring generation and storage capacity to exceed demand by a marginal percentage. This makes the energy system more resilient. For those emerging technologies that are not available in the initial modeling year (e.g. hydrogen production), we introduce a growth rate constraint to account for the realistic expansion. All additional constraints are included in the Supporting Information.

## Supplementary Material

pgaf389_Supplementary_Data

## Data Availability

The detailed discussions on data processing are available in the Supporting Information (Tables S1–S5 and Figs. S1–S6). Computer codes are available at https://doi.org/10.5281/zenodo.17824323.

## Preprints

This manuscript was posted on a preprint at http://dx.doi.org/10.2139/ssrn.4929232.

## References

[1] Haraguchi N, Cheng CFC, Smeets E. 2017. The importance of manufacturing in economic development: has this changed? World Dev. 93:293–315.

[2] ACE . The 7th ASEAN energy outlook. ACE, Jakarta, 2022.

[3] IEA . World energy outlook 2023. IEA, Paris, 2023.

[4] IEA . 2023. IEA World Energy Statistics and Balances. [accessed 10 Oct 2023]. https://www.oecd-ilibrary.org/energy/data/iea-world-energy-statistics-and-balances_enestats-data-en.

[5] Aleluia J, Tharakan P, Chikkatur AP, Shrimali G, Chen X. 2022. Accelerating a clean energy transition in Southeast Asia: role of governments and public policy. Renew Sust Energ Rev. 159:112226.

[6] Paltsev S, Mehling M, Winchester N, Morris J, Ledvina K. Pathways to Paris: ASEAN. MIT, Boston, 2018.

[7] Government of Singapore . 2022. Singapore's Fifth National Communication and Fifth Biennial Update Report. [accessed 10 Dec 2023]. https://unfccc.int/sites/default/files/resource/Singapore - NC5BUR5.pdf.

[8] Dorfner J . 2023. URBS: A Linear Optimisation Model for Distributed Energy Systems. [accessed 10 May 2024]. https://urbs.readthedocs.io/en/latest/.

[9] Zhong S, et al 2025. Accelerating ASEAN's energy transition in the power sector through cross-border transmission and a net-zero 2050 view. iScience. 28:111547. 39801835 10.1016/j.isci.2024.111547 PMC11719848

[10] Handayani K, et al 2022. Moving beyond the NDCs: ASEAN pathways to a net-zero emissions power sector in 2050. Appl Energy. 311:118580.

[11] IEA . An energy sector roadmap to net zero emissions in Indonesia. IEA, Paris, 2022.

[12] Lu B, Blakers A, Stocks M, Do TN. 2021. Low-cost, low-emission 100% renewable electricity in Southeast Asia supported by pumped hydro storage. Energy. 236:121387.

[13] IRENA . Renewable energy outlook for ASEAN: towards a regional energy transition. 2nd ed. IRENA, Abu Dhabi, 2022.

[14] Vakulchuk R, Overland I, Suryadi B. 2023. ASEAN's energy transition: how to attract more investment in renewable energy. Energy Ecol Environ. 8:1–16.

[15] Dutu R . 2016. Challenges and policies in Indonesia's energy sector. Energy Policy. 98:513–519.

[16] Nong D, Wang C, Al-Amin A. 2020. A critical review of energy resources, policies and scientific studies towards a cleaner and more sustainable economy in Vietnam. Renew Sust Energ Rev. 134:110117.

[17] IEA . 2021. Climate Impacts on South and Southeast Asian Hydropower. [accessed 25 Apr 2022]. https://www.iea.org/reports/climate-impacts-on-south-and-southeast-asian-hydropower.

[18] Liu L, Hejazi M, Iyer G, Forman BA. 2019. Implications of water constraints on electricity capacity expansion in the United States. Nat Sustain. 2:206–213.

[19] Siala K, Chowdhury AK, Dang TD, Galelli S. 2021. Solar energy and regional coordination as a feasible alternative to large hydropower in Southeast Asia. Nat Commun. 12:4159. 34230491 10.1038/s41467-021-24437-6 PMC8260807

[20] UN ESCAP . 2022. Toward Sustainable Energy Connectivity in Asia and the Pacific: Status, Trends, and Opportunities. [accessed 24 Mar 2023]. https://www.unescap.org/kp/2022/toward-sustainable-energy-connectivity-asia-and-pacific-status-trends-and-opportunities.

[21] Ahmed T, et al 2017. ASEAN power grid: a secure transmission infrastructure for clean and sustainable energy for South-East Asia. Renew Sust Energ Rev. 67:1420–1435.

[22] EMA . 2022. Charting the Energy Transition to 2050: Energy 2050 Committee Report. [accessed 28 Mar 2023]. https://www.ema.gov.sg/energy-2050-committee-report.aspx.

[23] IEA . CCUS In clean energy transitions. IEA, Paris, 2020.

[24] IEA . World energy outlook 2022. IEA, Paris, 2022.

[25] IEA . Carbon capture, utilisation and storage: the opportunity in Southeast Asia. IEA, Paris, 2021.

[26] Li YE, et al 2022. CO2 Transport and Storage Feasibility and Cost Study for ASEAN. [accessed 10 Jul 2024]. https://eartharxiv.org/repository/view/4676/.

[27] NCCS . Study of hydrogen imports and downstream applications for Singapore. National Climate Change Secretariat, Singapore, 2021.

[28] ACE . 2021. Hydrogen in ASEAN: Economic Prospects, Development, and Applications. [accessed 1 Oct 2022]. https://aseanenergy.org/hydrogen-in-asean-economic-prospects-development-and-applications/.

[29] ExxonMobil . 2024. ExxonMobil and Shell Selected to Work with the Government of Singapore on a Carbon Capture and Storage Value Chain. [accessed 10 Jun 2024]. https://corporate.exxonmobil.com/locations/singapore/singapore-updates/news-releases/03012024_exxonmobil-and-shell-selected-to-work-with-the-govt-of-singapore-on-a-ccs-value-chain.

[30] MTI . 2022. Singapore's National Hydrogen Strategy. [accessed 10 Aug 2025]. https://www.mti.gov.sg/Industries/Hydrogen.

[31] Fahim KE, De Silva LC, Hussain F, Shezan SA, Yassin H. 2023. An evaluation of ASEAN renewable energy path to carbon neutrality. Sustainability. 15:6961.

[32] Yang F, Li CT. 2024. The Status quo, dilemma, and transformation path of the carbon neutrality-related policy of the ASEAN. Sustainability. 16:1348.

[33] Lau HC . 2023. Decarbonization of ASEAN's power sector: a holistic approach. Energy Rep. 9:676–702.

[34] Nian VC, Mignacca B, Locatelli G. 2022. Policies toward net-zero: benchmarking the economic competitiveness of nuclear against wind and solar energy. Appl Energy. 320:119275.

[35] Heffron RJ, Merdekawati M, Suryadi B, Yurnaidi Z. 2024. Defining a ‘just energy investment’ for the ASEAN just transition. Environ Sustain Indic. 22:100367.

[36] Tan Y, Uprasen U. 2021. Carbon neutrality potential of the ASEAN-5 countries: implications from asymmetric effects of income inequality on renewable energy consumption. J Environ Manage. 299:113635. 34481375 10.1016/j.jenvman.2021.113635

[37] Gulagi A, Bogdanov D, Breyer C. 2017. A cost optimized fully sustainable power system for Southeast Asia and the Pacific Rim. Energies (Basel). 10:583.

[38] Chang YH, Han P. 2021. Harnessing wind energy potential in ASEAN: modelling and policy implications. Sustainability. 13:4279.

[39] Ahmed T, Mekhilef S, Shah R, Mithulananthan N. 2017. Investigation into transmission options for cross-border power trading in ASEAN power grid. Energy Policy. 108:91–101.

[40] Chang Y, Lee J, Ang XW, Chua YJ. 2019. Energy market integration in ASEAN: locational marginal pricing and welfare implications. J Asian Econ Integr. 1:48–72.

[41] Chang YH, Li YF. 2013. Power generation and cross-border grid planning for the integrated ASEAN electricity market: a dynamic linear programming model. Energy Strategy Rev. 2:153–160.

[42] Kang JD, Wu ZC, Ng TS, Su B. 2023. A stochastic-robust optimization model for inter-regional power system planning. Eur J Oper Res. 310:1234–1248.

[43] ACE . The 8^th^ ASEAN energy outlook. ACE, Jakarta, 2024.

[44] Handayani K, Overland I, Suryadi B, Vakulchuk R. 2023. Integrating 100% renewable energy into electricity systems: a net-zero analysis for Cambodia, Laos, and Myanmar. Energy Rep. 10:4849–4869.

[45] Huber M, Roger A, Hamacher T. 2015. Optimizing long-term investments for a sustainable development of the ASEAN power system. Energy. 88:180–193.

[46] U.S. Department of Energy . 2021. Hydrogen Shot. [accessed 20 May 2025]. https://www.energy.gov/sites/default/files/2021-08/factsheet-hydrogen-shot-introduction-august2021.pdf.

[47] Zsiborács H, et al 2019. Intermittent renewable energy sources: the role of energy storage in the European power system of 2040. Electronics (Basel). 8:729.

[48] Dorfner J, et al tum-ens/urbs: urbs v1.0.1. Zenodo; 2019. [accessed 22 May 2023]. https://zenodo.org/records/3265960.

[49] Stich J, Massier T. 2015 IEEE Pes Asia-Pacific Power and Energy Engineering Conference Asia-Pacific Power and Energy Engineering Conference. IEEE; 2015.

[50] IEA . Southeast Asia energy outlook 2019. IEA, Paris, 2019.

