# Supplementary material for: Charting net-zero pathways for ASEAN's energy sector
Source: PNAS Nexus. 2026 Jan 6;5(1):pgaf389. doi: 10.1093/pnasnexus/pgaf389 (PMC12771515; doi:10.1093/pnasnexus/pgaf389)
Supplement: pgaf389_Supplementary_Data [file pgaf389_supplementary_data.pdf]

Charting Net-Zero Pathways for ASEAN's Energy Sector
Supporting Information

1 Model

1.1 Basic Capacity Expansion Model

The basic model formulation is available in the URBS model documentation [1] and Zhong, Yang [2]. Detailed information of the objective function and common model constraints are shown below.

Indices:

V	The set of regions
E	Set of plausible transmission lines from region v_1 to v_2, $E = \{(v_1, v_2)\|v_1, v_2 \in V\}$
Y	Set of modelled years $y \in Y$, $Y := \{y_1, \dots, y_{\|Y\|}\}$
T	Set of modelled time periods $t \in T$, $T := \{t_1, \dots, t_{\|T\|}\}$
G	The set of generation technologies $g \in G$
P	The set of available generation technologies in different regions, $P = \{(g, v)\|g \in G, v \in V\}$
B	The set of available storage technologies in different regions, $B = \{b\|b \in V\}$
C	The set of commodities, including the set of fuels C^F and CO_2 emissions, $C = C^F \cup \{CO_2\}$
A	The set of all assets, including all available generation facilities and all transmission lines, $A = P \cup E \cup B$
$Y(y, a)$	The set of modelled years that an asset $a \in A$ remaining operational if it is installed in year $y \in Y$; we further define $Y(0, a)$ as the set of operational years of the initially installed asset a.
S	The set of technology groups; the members of a group $s \in S$ share the same capacity limits.
P_S	The set of generation technologies (g, v) in group $s \in S$

Parameters:

D_{yvt}	Projected electricity demand for region $v \in V$ in year $y \in Y$ at time $t \in T$
$K_{ya}^{Capex}, K_{ya}^{Fix}$	The unit CAPEX cost and FOM cost (USD per MW) of an asset $a \in P \cup E \cup B$ in year y respectively.
K_{ya}^{Var}, K_{ya}^{F}	The unit variable cost and unit fuel cost (USD per MWh) of an asset $a \in P \cup E \cup B$ in year y respectively.
$\hat{\kappa}_{0a}$	Installed capacity of asset a in the initial year.
$\bar{\hat{\kappa}}_{0ay}$	The upper bound if adding new asset a in year y.
$\underline{\kappa}_{ya}, \overline{\kappa}_{ya}$	The lower and upper bound of asset a in year y.
$\underline{\kappa}_{ys}, \overline{\kappa}_{ys}$	The upper bound of technology group S in year y.
n_a	The lifetime of an asset $a \in P \cup E$
$l_{v_1 v_2}$	The transmission efficiency factor.
$\underline{\lambda}_{yv}, \overline{\lambda}_{yv}$	The minimum and maximum electricity net import for region v in year y.
$e_{yb}^{in}, e_{yb}^{out}, d_{yb}^{con}$	The electricity charging and discharging efficiencies, and hourly discharge rate of storage $b \in B$.
$k_{yb}^{P/C}$	The power to capacity ratio of storage $b \in B$

$\underline{\eta}_{ya}, \overline{\eta}_{ya}$	The minimum and maximum capacity factor for an infrastructure asset a in year y.
T	The number of hours per year (8760 hours)
F_{ygvc}	The input (or output) ratio of commodity $c \in C$, in each asset (g, v)
$\overline{\rho}_{yvc}$	The upper bound of commodity $c \in C$ in region v in year y

Decision variables:

ζ	Total cost
$\kappa_{ya}, \hat{\kappa}_{ya}$	Total available and newly installed capacity of an asset $a \in P \cup E$ in year y, respectively.
τ_{ygvt}	The electricity throughput of a process $(g, v) \in P$ in time t of year y.
$\pi^{in}_{yv_1v_2t}, \pi^{out}_{yv_1v_2t}$	The net electricity input and output of a transmission line $(v_1, v_2) \in E$ in time t of year y.
$\varepsilon^{in}_{ybt}, \varepsilon^{out}_{ybt}, \varepsilon^{con}_{ybt}$	The electricity input, output and storage condition of battery $b \in B$ in time t of year y.
ρ_{ygvc}	The input (or output) of commodity

The model minimizes the total system costs, including the net present value (NPV) of total capital expenditure (CAPEX) costs ζ^{Capex} of newly built generation plants and transmission capacities, the fixed O&M (FOM) costs ζ^{FOM} of these assets, the variable costs ζ^{Var} and the fuel costs ζ^{F} for electricity generation. The transmission grids are modelled as directed arcs that connect the nodes, with two separate directions for each transmission line. A coefficient of 1/2 is used to avoid overcounting the CAPEX costs and fixed costs of a transmission line. The total costs are calculated as follows:

$$\min \zeta = \sum_{y \in Y} \sum_{a \in P} \zeta^{Capex}_{ya} + \sum_{y \in Y} f_y(\omega_y) \cdot \left(\sum_{a \in P} \left(\zeta^{FOM}_{ya} + \zeta^{Var}_{ya} \right) + \sum_{c \in C^F, v \in V, t \in T} \zeta^{F}_{yvc} \right) + \frac{1}{2} \sum_{y \in Y, a \in E} \left(\zeta^{Capex}_{ya} + f_y(\omega_y) \zeta^{FOM}_{ya} \right) \tag{1}$$

where $f_y(\omega_y)$ is the NPV factor for the annuitized cost in year y with a weight ω_y. The weight of a modelled year y_n equals the length of the time interval between year y_n and the next modelled year y_{n+1} , i.e., $\omega_{y_n} = y_{n+1} - y_n$. Therefore, the NPV factor calculated as $f_y(\omega) = (1 + j)^{1-(y-y_1)} \frac{1-(1+j)^{-\omega}}{j}$, where j is the discount rate and y_1 represents the initial year. Essentially, the function $f_y(\omega)$ gives the NPV factor of annuities that start in year y and repeat ω_y times. It implies that the same cost occurs in each year represented by the modelled year y. The CAPEX of the newly installed capacity of asset a in year y is computed as the sum of annuities to distribute equally over the lifetime of the asset. Only the payments that fall within the planning horizon are counted and discounted to the commission year. The cost is calculated as follows:

$$\zeta^{Capex}_{ya} = f_y(n_{ya}) f_a K^{Capex}_{ya} \hat{\kappa}_{ya} \tag{2}$$

where $n_{ya} = \min(n_a, y_{|Y|} - y)$ denotes the number of payments that fall in the planning horizon and $f_a = \frac{i \cdot (1+i)^{n_a}}{(1+i)^{n_a} - 1}$ is the capital recovery factor with interest rate i and asset lifetime n_a.

The rest types of costs are calculated as:

$\zeta_{ya}^{FOM} = K_{ya}^{FOM}\kappa_{ya}$		(3)
$\zeta_{ya}^{Var} = K_{ya}^{Var}\sum_{t\in T}\tau_{yat}, \qquad a \in P$		$(4a)$
$\zeta_{ya}^{Var} = K_{ya}^{Var}\cdot\sum_{t\in t}\left(\pi_{yat}^{in} + \pi_{yat}^{out}\right), \qquad a \in E$		$(4b)$
$\zeta_{ya}^{Var} = K_{ya}^{Var}\cdot\sum_{t\in t}\left(\varepsilon_{yat}^{in} + \varepsilon_{yat}^{out}\right), \qquad a \in B$		$(4c)$
$\zeta_{yvc}^{F} = \sum_{g:(g,v)\in P, t\in t} K_{yrc}^{F}\rho_{ygvct}$		(5)

The linear programming model is subject to a set of constraints on the properties of the power system, for electricity dispatch, generation capacity expansion, transmission and storage (see below).

Capacity equations:		
$\min\zeta$<br>subject to:		(6)
$\kappa_{ya} = \sum_{y'\in Y(y,a)}\hat{\kappa}_{ya} + \mathbf{I}_{Y(0,a)}(y)\kappa_{0a}, \qquad \forall y\in Y, a\in A$<br>Balance of capacity		(7)
$\underline{\kappa}_{ya} \le \kappa_{ya} \le \overline{\kappa}_{ya}, \qquad \forall y \in Y, a \in A$<br>Upper and lower bound constraints on installed capacity.		(8)
$\hat{\kappa}_{ya} \le \hat{\overline{\kappa}}_{ya}, \qquad \forall y\in Y, a\in A$<br>Upper bound constraints on installed capacity		(9)
**Generation process Equations:**		
$T\underline{\eta}_{ygv}\kappa_{ygv} \le \tau_{ygv} \le T\overline{\eta}_{ygv}\kappa_{ygv}, \qquad \forall y\in Y, (g,v)\in P$<br>Balance of generation throughput.		(10)
$\rho_{ygvc} = F_{ygc}\tau_{ygv}, \qquad \forall y\in Y, (g,v)\in P, c\in C$<br>Resource consumption process		(11)
$\sum_{g\in G}\rho_{ygvc} \le \overline{\rho}_{yvc}, \qquad \forall y\in Y, (g,v)\in P, c\in C$<br>Balance of generation throughput		(12)
$\underline{\kappa}_{ys} \le \sum_{a\in P_s}\kappa_{ya} \le \overline{\kappa}_{ys}, \qquad \forall y\in Y, (g,v)\in P, s\in S$<br>Generation group capacity limits		(13)
**Transmission equations**		
$T\underline{\eta}_{yvv'}\kappa_{yvv'} \le \pi_{yvv't}^{in} \le T\overline{\eta}_{yvv'}\kappa_{yvv'}, \qquad \forall y\in Y, \forall t\in T, (v,v')\in E$<br>Transmission throughput constraint.		(14)
$l_{vv'}\cdot\pi_{yvv't}^{in} = \pi_{yvv't}^{out}, \qquad \forall y\in Y, \forall t\in T, (v,v')\in E$<br>Transmission efficiency constraint		(15)
$\kappa_{y,v_1,v_2} = \kappa_{y,v_2,v_1}, \qquad \forall y\in Y, (v_1,v_2)\in E$<br>Symmetry rule: Transmission lines		(16)
$\underline{\lambda}_{yv} \le \sum_{t\in T}\left(\sum_{v':(v',v)\in E}\pi_{yv'vt}^{out} - \sum_{v':(v,v')\in E}\pi_{yvv't}^{in}\right) \le \overline{\lambda}_{yv}, \qquad \forall y\in Y, v\in V$<br>Balance of net import		(17)

**Storage equations**		
$\kappa^{P}_{yb} = \kappa^{C}_{yb} k^{P/C}_{yb}, \quad \forall y \in Y, b \in B$ Power to storage ratio		(18)
$\varepsilon^{con}_{ybt_1} = \kappa^{C}_{yb} I_{yb}$ Initial charge constraint		(19)
$\varepsilon^{in,out}_{ybt} \leq \kappa^{P}_{yb}, \quad \forall y \in Y, b \in B$ Constraint on storage throughput.		(20)
$\varepsilon^{con}_{ybt} \leq \kappa^{C}_{yb}, \quad \forall y \in Y, b \in B$ Constraint on storage capacity.		(21)
$\varepsilon^{con}_{ybt} = \varepsilon^{con}_{yb(t-1)} \cdot \left(1 - d^{con}_{yb}\right) + \varepsilon^{in}_{ybt} \cdot e^{in}_{yb} - \frac{\varepsilon^{out}_{ybt}}{e^{out}_{yb}}, \quad \forall y \in Y, \forall t \in T, b \in B$ Balance of Storage capacity		(22)
$\varepsilon^{con}_{ybt_1} \leq \varepsilon^{con}_{ybt_N}, \quad \forall y \in Y, b \in B$ Constraint on initial and final condition.		(23)
**General Equations:**		
$\sum_{(g,v) \in P} \tau_{ygvt} + \left(\sum_{v':(v',v) \in E} \pi^{out}_{yv'vt} - \sum_{v':(v,v') \in E} \pi^{in}_{yvv't} \right) + \left(\varepsilon^{out}_{yvt} - \varepsilon^{in}_{yvt} \right) \geq D_{yvt}$ $, \forall y \in Y, \forall t \in T, v \in V$ Vertex rule		(24)
$\boldsymbol{\zeta}, \boldsymbol{\kappa}, \boldsymbol{\hat{\kappa}}, \boldsymbol{\tau}, \boldsymbol{\dot{\tau}}, \boldsymbol{\pi}, \boldsymbol{\dot{\pi}}, \boldsymbol{\varepsilon}, \boldsymbol{\dot{\varepsilon}} \geq 0, \boldsymbol{\rho} \in \mathbb{R}$		(25)

The rules for capacity balance and expansion are similar across generation infrastructure P, transmission lines E, and storage infrastructure B, as summarized by constraints (7-9). Constraint (7) maintains the electricity balance for each node. It ensures that domestic electricity generation in each country, as well as that net imports from other countries, satisfy projected demand D_{yr} in each country and year. The imports from other countries are discounted with the transmission efficiency factor $l_{v_1 v_2}$, due to the electricity loss on transmission lines.

Constraints (8) capture each region's preferences regarding electricity trade, which provide boundaries on the annual net import in both directions for each country and year. A value of $\overline{\lambda}_{yv} = 0$ means that the region does not import electricity for its domestic consumption, while $\underline{\lambda}_{yv} = 0$ means that the region is not willing to export electricity.

Constraint (9) gives an upper-bound on the maximum expansion rate for each type of asset. Such bounds reflect conceivable policies, such as the restriction on coal generation technology.

The rules for generation technology are shown in Constraints (10 - 13). Constraint (10) limits the amount of electricity generated by the total operational capacity of the corresponding asset and its annual capacity factor. Constraint (11) computes the commodity inputs and outputs of the electricity generation process $(g, v) \in P$ per timepoint per year. The total amount of commodity c associated with a generation facility is pro-rated to the electricity generation with an input (or output) ratio. For fuels $c \in C^F$, $\frac{1}{F_{ygc}}$ represents the efficiency of the generation technology when fuel c is fed. Constraint (12) limits the maximum allowance for each commodity per region and year. Constraints (13) sets group capacity limits for groups of generation infrastructure, as one form of energy can be harnessed by multiple competing technologies.

The rules for transmission technologies are covered by Constraints (14 - 17). Constraint (14) limits the amount of electricity exported out of a transmission line by the total operational capacity factor and its annual capacity factor. Constraint (15) computes the power output of the transmission line based on the power input and the efficiency of transmission. Constraint (16) ensures that two directions of a transmission line have equal capacities.

Constraint (17) capture each region's preferences regarding electricity trade, which provide boundaries on the annual net import in both directions for each country and year. A value of $\overline{\lambda}_{yv} = 0$ means that the region does not import electricity for its domestic consumption, while $\underline{\lambda}_{yv} = 0$ means that the region is not willing to export electricity.

The rules for battery storage are covered by Constraints (18 – 23). Constraint (18) computes the charging/discharging capacity of the installed batteries based on the energy capacity and the power to energy ratio. Constraint (19) computes the initial state of the battery based on the installed capacity and initial charge fraction. Subsequently constraint (20 – 21), constrain the amount storage is charged and discharged and the storage condition to the rated power and energy capacity. At any given time point, constraint (22) computes the total storage condition of the battery based on the previous time periods storage capacity, the amount of power charged, and discharged from the battery. Finally, to ensure that value isn't extracted from the battery by extracting the storage content over at the end of a simulated year, the initial and final storage condition of the battery are linked using constraint (23).

To ensure overall energy balance of all countries at any given time point and year, the power produced by all generation technologies, and the movement of power into and out of transmission lines and battery storage, are linked by constraint (24).

1.2 Power reserve margins

We have introduced power reserve margins to the model. Although URBS optimizes long-term capacity expansion, as a deterministic model it does not explicitly consider resilience to electricity demand fluctuations or generator outages. Therefore, a set of constraints were revised, requiring firm generator and storage capacity to exceed electricity demand by a minimum percentage [3]. This margin can be provided by a set of process $p \in P_{reserve}$, including non-variable renewables, fossil fuel power plants and battery storage, to provide spinning reserves. The constraints are:

$$\tau_{pt} + \tau_{pt}^{R} \leq \Delta t\, \kappa_p, \forall p \in P_{reserve}, t \in T_m \quad (25)$$

$$\epsilon_{pct}^{in} = r_{pc}^{in}\left(\tau_{pt} + \tau_{pt}^{R}\right), \forall p \in P_{reserve}, c \in C_{sup}, t \in T_m \quad (26)$$

$$\epsilon_{pct}^{out} = r_{pc}^{out}\tau_{pt}^{R}, \forall p \in P_{reserve}, c \in C_{reserve}, t \in T_m \quad (27)$$

$$\sum_t \tau_{pt}^{R} - r_p^{R}\sum_t \tau_{pt} \leq 0, \forall p \in P_{reserve}, t \in T_m \quad (28)$$

$$\epsilon_{st}^{R} \leq \epsilon_{st}^{c}, \forall s \in S, t \in T_m \quad (29)$$

$$\epsilon_{st}^{in} + \epsilon_{st}^{out} + \epsilon_{st}^{R} \leq \Delta t \cdot \kappa_s^{p}, \forall s \in S, t \in T_m \quad (30)$$

$$CB(c,t) = -\sum_{(c,p)\in C_p^{out}} \epsilon_{pct}^{out} - \sum_{(s,c)\in C_s^{R}} \epsilon_{st}^{R} \leq -d_{ct}, \forall c \in C_{reseve}, t \in T_m \quad (31)$$

The operational state of the process is captured by two variables, where the process throughput τ_{pt} is for active electricity generation and reserve throughput τ_{pt}^{R} for unused capacity that can be drawn upon when needed. The total throughput in any time step t is limited by the total installed capacity

κ_p and the length of the discretization time step Δt as shown in constraint (25). While for renewable generation that depends on intermittent resources, the total throughput depends on the availability of the input commodity as indicated in constraint (26), reserved throughput requires no fuel inputs as it represents unused capacity. Reserve throughput has only one output, which is the reserved power ϵ_{pct}^{out}, as indicated in constraint (27). Finally, constraint (28) ensures that the process that provides reserve must be in active running status, by restricting that the reserved throughput cannot exceed a factor r_p^R times the total actual electricity generation $\sum_t \tau_{pt}$ from the process.

In addition to generation capacity, storage technologies may also provide reserve to the system, denoted by ϵ_{st}^R. The reserve provision from storage is constrained by both the storage energy level ϵ_{st}^c and the total power capacity κ_s^p, as constraints (29) and (30) indicate.

Finally, the provision of reserve must meet the total demand for reserves. Specifically, the total reserve output from generation power plants and storage must be greater than the total demand for reserves, following Kirchhoff's current law as indicated by constraint (31).

1.3 Maximum growth rates of novel technologies

The model considers long-term capacity expansion for novel technologies that are not yet commercially available (e.g., MP gas-to-H2). To account for the realistic ramp-up of such technologies, constraint (32) is introduced to limit the growth rate. Let y_i and y_{i+1} denote two consecutive modelling years that are k years apart. The new capacity addition for process $p \in P$ expanded at the beginning of y_{i+1}, denoted as $\hat{\kappa}_{y_i+1,p}$, is restricted to not exceed an annual growth rate $r_{y_ip}^{GR}$ times the number of years k times available capacity in year y_i, denoted as κ_{y_ip}. In case the available capacity κ_{y_ip} is 0, a minimum capacity addition δ_{y_ip} is still allowed.

$$\hat{\kappa}_{y_i+1,p} \leq r_{y_ip}^{GR} \cdot k \cdot \kappa_{y_ip} + \delta_{y_ip}, \forall (y_{i+1}, y_i) \in Y, p \in P \qquad (32)$$

2 Demand projections and electricity load profiles

Given the socio-economic prospects for ASEAN, the demand for electricity and potentially hydrogen in this region will grow substantially. Historical electricity demand by country (prior to 2025) is taken from IEA [4], and electricity demand projections are taken from Zhong et al. [2], which are consistent with projections from IEA [5], IRENA [6].

Historical data on ASEAN-level hydrogen demand are based on an earlier version of the IEA World Energy Outlook [7]. To derive ASEAN-level hydrogen demand projections under net-zero emissions by 2050, we first calculate the growth of global hydrogen demand under the IEA's Announced Pledges Scenario (APS) relative to that under its Net-Zero Scenario IEA [8]. Then, we apply such increases to ASEAN's hydrogen demand under APS [8]. ASEAN's hydrogen demand will increase from 3.51 Mt H2 in 2018 to 32.47 Mt H2. We apply the country's shares in hydrogen demand reported in NCCS [9] to obtain the hydrogen demand by country. As hydrogen demand data for ASEAN in IEA [8] are only available for 2030 and 2050, we interpolate the hydrogen demand for the remaining modelling years.

Hourly electricity load data for representative days throughout the year are required for each country. We collect the data of hourly electricity load profiles from official sources where available, including national energy authorities or companies [10-12] and Handayani, Anugrah [13]. Figure S1 presents the 288-hour electricity load profiles for all ASEAN countries (expressed as a percentage of system load).

The load curves of the Philippines and Singapore exhibit less fluctuation, whereas the changes in electricity load within a day and across days are greater in other countries.

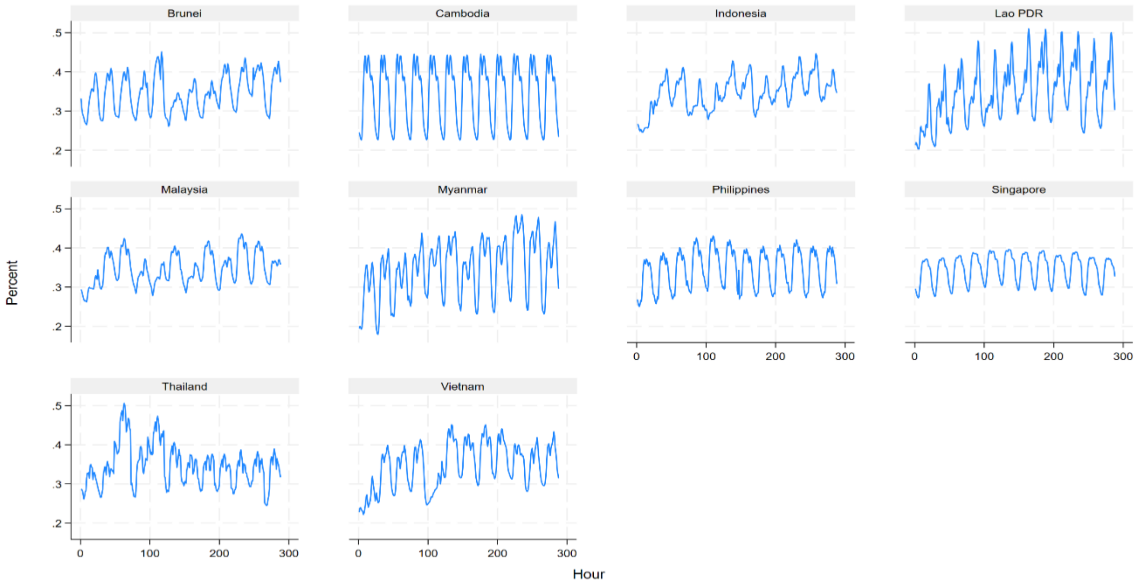

Figure S1. Electricity load profiles of ASEAN countries

3 Techno-economic inputs of technologies

This study considers two types of energy processes—electricity generation and hydrogen production—each covering conventional fossil fuel-based technologies, renewable energy, and emerging technologies, such as CCS. Where available, it uses ASEAN-specific techno-economic data to characterise ASEAN's local circumstances, making it relevant to ASEAN energy sector planning. Table S1 summarizes the techno-economic inputs for the initial modelling year. The technologies in this table are used in domestic production of electricity and hydrogen.

Several emerging technologies' CAPEX is perceived to decline through 2050 (see Figure S2). For electricity generation, the CAPEX trajectories of coal with CCS, natural gas combined cycle with CCS, solar, onshore, and offshore wind are based on the CAPEX growth rates derived using NREL [14].[1] The CAPEX of biomass with CCS power plants is assumed to remain unchanged over time [15]. For hydrogen production, the CAPEX trajectories for electrolysis and gas-to-H2-CCS are taken from IEA [16]. Based on prototype applications, methane Pyrolysis (MP) gas-to-H2 is projected to have significant cost reductions [9], and MP gas-to-H2 is assumed to have the same CAPEX reduction rates as those of gas-to-H2-CCS.

Conventional natural gas and coal-fired power plants have lower thermal efficiency than other emerging Asian countries [17] and improvement in thermal efficiency is crucial for ASEAN [18]. Therefore, the efficiencies of natural gas and coal-fired power plants are assumed to increase to 58% and 51.55% by 2050, respectively, which is the average given to China and India [19]. The same efficiency growth rates are applied to those with CCS. For hydrogen production, electrolysis efficiency is projected to achieve 74% by 2050 [16].

[1] For example, the CAPEX of natural gas with CCS is projected to decrease from USD 1840 / kW in 2018 to USD 1171 / kW in 2050.

Table S1. Characteristics of power generation and hydrogen production technologies in 2018

Technology	CAPEX (USD / kW)	Fixed O&M cost (% of CAPEX)	Variable cost (USD / MWh)	Efficiency (%)	Lifetime (years)	Emission factor (kg CO_2 / kWh)
Power generation						
Oil	1310	3.52	2.5	34.11	30	1.28
Coal	1551	4.00	4.0	33.52	40	0.99
Natural gas	1042	3.52	2.3	42.63	40	0.45
Hydro	1597	0.50	0	N.A.	50	0
Geothermal	3001	2.00	0	N.A.	30	0
Solar	1487	2.30	0	N.A.	20	0
Onshore wind	2168	3.00	0	N.A.	20	0
Offshore wind	3090	3.00	0	N.A.	20	0
Biomass	2322	2.00	6.0	35.00	20	0
Coal-CCS	3470	2.84	25.1	26.22	40	0.10
Natural gas-CCS	1840	1.77	15.9	37.89	30	0.04
Biomass-CCS	5453	1.17	34.9	26.81	20	-1.31
H2-to-electricity	1042	3.52	2.3	42.63	30	0
Hydrogen production						
Solar-to-H2	2598	4.70	0	64.00	16	0
Wind-to-H2	3257	4.70	0	64.00	16	0
Gas-to-H2	910	4.70	0	76.00	40	0.32
Gas-to-H2-CCS	1583	3.00	0	69.00	40	0.03
Coal-to-H2	2672	5.00	0	60.00	40	0.54
Coal-to-H2-CCS	2783	5.00	0	58.00	40	0.05
MP gas-to-H2	3314	2.52	0	28.30	40	0

Notes: (1) N.A.: Not applicable. (2) We assume the CCS technologies have 90% CO_2 capture rates and that CCS is viable from 2030 and onwards. (3) The variable cost of CCS technologies includes the cost of CO2 transport and storage, assumed to be USD 20 / tonne CO2 [16]. (4) Electrolysis process is paired with solar (solar-to-H2) and onshore wind (wind-to-H2). This allows us to identify the renewable electricity source in hydrogen production, whereas a generic power-to-H2 technology may use the electricity from fossil fuels. (5) Revenue streams from hydrogen production are not considered.

Sources: For power generation, CAPEX [13, 20-24]; fixed O&M cost [14, 15, 23, 25]; variable cost [13, 14, 16, 25, 26]; efficiency [14, 17, 25, 26]; lifetime [21, 25, 27]; emission factor [4, 26, 28]. The detailed information of data collection and estimates is available in Zhong et al. [2]. For hydrogen production, CAPEX and fixed O&M cost [2, 16, 29, 30]; variable cost [16, 30]; efficiency [16, 29, 31]; lifetime [30]; emission factor [30, 32, 33].

This study considers battery storage (4-hour duration lithium battery), and onshore and offshore transmission technologies. Table S2 presents the techno-economic inputs based on Stich and Massier [27]. The CAPEX of battery storage is assumed to decline to USD 105 / kWh by 2050 (see Figure S2c) [14]. The techno-economic inputs of transmissions are applied to each transmission line in the model (see Table S2).

Table S2. Techno-economic data of energy storage and transmission technologies in 2018

(a). Energy storage

Energy storage	CAPEX (USD/kWh)	Fixed O&M cost (% of CAPEX)	Variable cost (USD/MWh)	Efficiency (%)	Lifetime (years)
Battery	300	1.36	0	85.00	15

(b). Transmission

Transmission technology	CAPEX (USD/MW km)	Fixed O&M cost (USD/km a)	Variable cost (USD/MWh)	Efficiency (%)	Lifetime (years)
HV onshore	500	10	0	90	40
HV offshore	3500	70	0	90	40

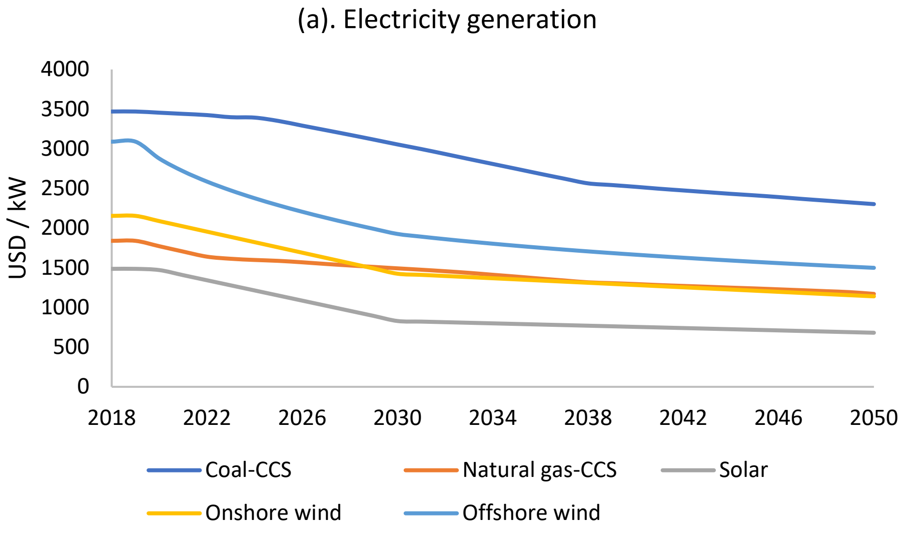

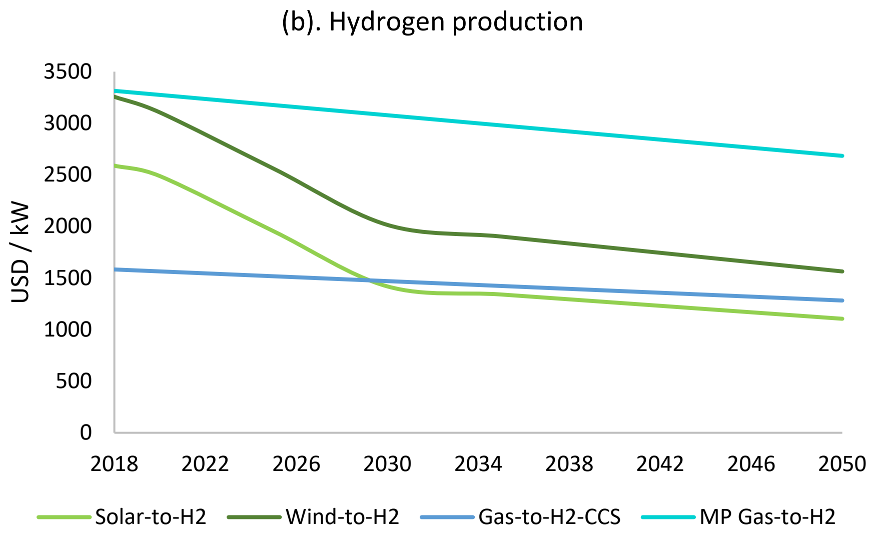

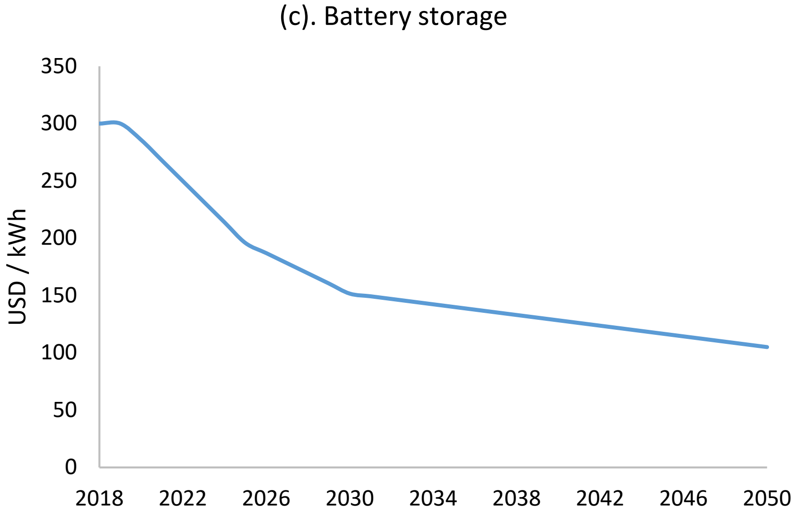

Figure S2. CAPEX reductions of selected technologies

Existing power generation profiles for each country (e.g., initial capacity and remaining lifetime) are mostly collected using plant-level microdata [20] for fossil fuel-based plants and IRENA [34] for renewables. Due to data availability, we assume no hydrogen production capacity for commercial use in the initial year.

4 Renewable resource potentials and hourly capacity factors

Table S3 presents the renewable resource potentials used in this study. We explicitly distinguish between the moderate and high potentials for hydro, solar, onshore wind and offshore wind. Moderate hydro potentials reflect historical trends in hydropower expansion, whereas high hydro potentials are based on literature surveys. Moderate variable renewable energy (VRE) potentials, i.e., solar, onshore wind and offshore wind, are those estimates from policy documents, whereas high VRE potentials are taken from those technical assessments.

Table S3. Renewable resource potentials (unit: GW)

Country	Hydro		Geothermal	Solar		Onshore wind		Offshore wind		Biomass
	High	Moderate		High	Moderate	High	Moderate	High	Moderate	
Brunei	0	0	0	16.0	2.5	0	0	0	0	0
Cambodia	10.0	5.5	0	3198.0	8.1	69.0	65.0	88.8	0	2.15
Indonesia	94.0	13.8	29.0	1052.0	208.0	61.0	61.0	589	0	33.00
Lao PDR	26.0	20.3	0	1278.0	8.8	13.0	3.5	0	0	1.20
Malaysia	35.0	20.6	0	1965.0	59	2.0	2.0	53.3	0	3.67
Myanmar	19.6	9.0	0	7717.0	30.0	482.0	6.97	0	0	0.99
Philippines	13.1	8.2	4.5	1910.0	45	217.0	5.54	69.4	0	4.45
Singapore	0	0	0	8.6	8.6	0	0	0	0	0.26
Thailand	6.4	4.1	0.0003	10538.0	22.8	239	17.00	29.6	0	7.00
Vietnam	35.0	35.0	0	2847.0	94.2	311	27.75	322.1	0	1.00

Sources: hydro (high) [13, 25, 35-38]; geothermal [35, 36]; solar (high) [21, 22]; solar (moderate) [25, 35, 38-43]; onshore wind (high): [21, 22] onshore wind (moderate) [35, 37-39, 42, 44, 45]; offshore wind (high) [6]; biomass [13, 25, 35, 46, 47].

5 Cross-border electricity transmission

Figure S3 shows the transmission network used in the current study in accordance with the literature [48, 49]. The transmission capacity includes existing capacity and those under construction and planning. The total planned capacity in the network is 29.75 GW, which is consistent with ACE [18]. We further consider restrictions for electricity imports. For Singapore, annual net electricity imports can provide up to 30% of the country's electricity demand by 2035 and up to 60% by 2050 [50]. The same restrictions are applied to Brunei. For the remaining countries, we assume that annual net imports can contribute up to 20% of domestic electricity demand by 2035 and up to 30% by 2050.

In current analysis, we do not consider the hydrogen storage and transport between ASEAN countries. Hydrogen can be produced locally or imported from outside ASEAN. In this study, Australia, i.e., the Australian Renewable Energy Hub, is identified to be the sourcing country of hydrogen outside ASEAN.

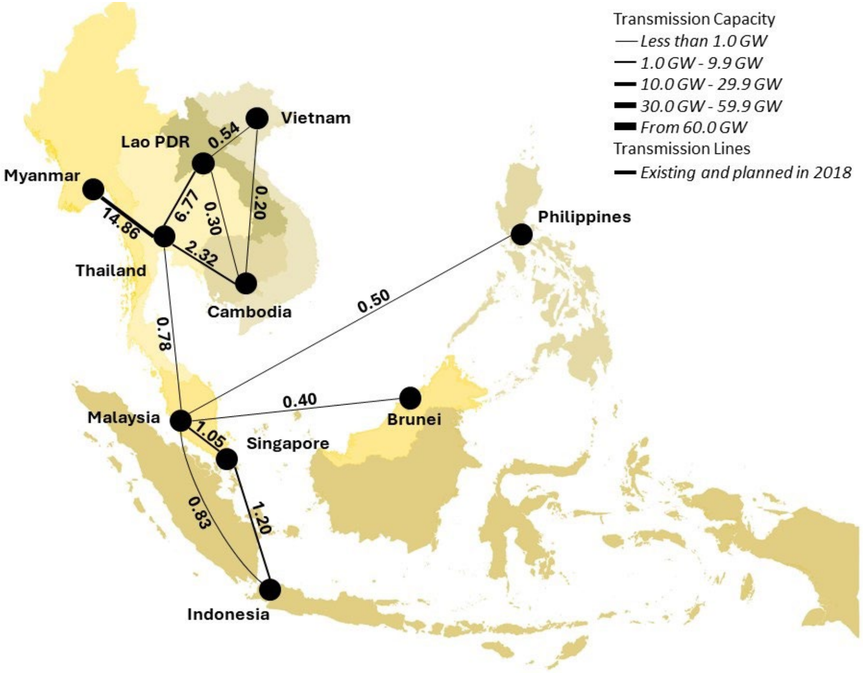

Figure S3. Transmission network of the ASEAN Power Grid in initial year

Note that (1) the value on the edge indicates the transmission capacity (GW) that is existing and under construction and planning in the initial year. Expansion in electricity transmission capacity takes place on the transmission lines in this figure. (2) Transmission lines are not drawn to scale. The locations and lengths of transmission lines do not reflect the actual projects.

Data on transmission lines are originally collected from literature [48, 49, 51], as shown in Table S4.

Table S4. Planned transmission lines

Transmission line	Capacity (GW)	Length (km)	Type
Thailand - Lao PDR	6.77	213	HV onshore
Lao PDR - Vietnam	0.54	192	HV onshore

Thailand - Myanmar		14.86	450	HV onshore
Vietnam - Cambodia		0.20	88	HV onshore
Lao PDR - Cambodia		0.30	56	HV onshore
Thailand - Cambodia		2.32	290	HV onshore
Malaysia - Singapore		1.05	24	HV onshore
Malaysia - Indonesia	Malaysia (Peninsular) - Indonesia (Sumatra)	0.60	295	HV offshore
	Malaysia (Sarawak) - Indonesia (West Kalimantan)	0.23	300	HV onshore
Philippines (Palawan) – Malaysia (Sabah)		0.50	529	HV offshore
Malaysia (Sarawak) - Brunei		0.40	111	HV onshore
Thailand - Malaysia		0.78	132	HV onshore
Indonesia - Singapore	Indonesia (Batam) - Singapore	0.60	38	HV offshore
	Indonesia (Sumatra) - Singapore	0.60	84	HV offshore

6 Climate targets

All ASEAN countries have announced their commitments to mitigate climate change under the Paris Agreement. Such climate targets are set for 2030 and expressed in either emissions intensity reduction or emissions reduction. We rely on countries' NDC submissions to the UNFCCC to derive their unconditional NDC targets (in terms of emissions reduction) for the power sector, except for Lao PDR, Malaysia and the Philippines, in which data from literature are used [36]. Beyond the 2030 NDCs, several ASEAN countries have established net-zero emissions targets for 2050 (e.g., Brunei, Malaysia, Singapore and Vietnam) or the post-2050 period (e.g., Indonesia and Thailand) [8].

7 Fuel prices

The URBS model expresses all fuel prices in USD / MWh. The projections of fossil fuel and biomass prices in Figures S4(a) and S4(b) are taken from Zhong et al. [2]. Thailand's residual fuel oil price is chosen as the representative ASEAN price in the initial year [52], while the fuel oil price projections are based on the oil price growth rates in the U.S. power sector [53].

Australia's coal price (6000 kcal/kg) is the representative price for the Asia-Pacific region. We collect data on historical coal prices from the World Bank [54] and the coal price projections from OPIS [55]. The LNG price of Japan hub is used as the reference natural gas price for Asia. The natural gas prices are taken from the World Bank [54] for 2025 and before. For the period 2025 – 2050, the natural gas price projections are based on the natural gas price growth rates in IEA [7], IEA [56].

The biomass price in the initial year (i.e., USD 5.03 / MWh) is taken from the literature [2], corresponding to an average biomass price of USD 20 / tonne [57]. Long-run price trajectories are based on the biomass price growth rates in Paardekooper, Lund [58].

Hydrogen import prices in Figure S4(c) are obtained from NCCS [9]. In current model, Australia is identified as the major sourcing country for the hydrogen import in ASEAN, due to Australia's significant solar and wind potentials and the policy support for hydrogen production and export [9]. Hydrogen import price is based on the hydrogen production in the Australian Renewable Energy Hub, and thus it reflects the import price of low-carbon hydrogen. In current mode, we do not consider the hydrogen storage and transport between ASEAN countries.

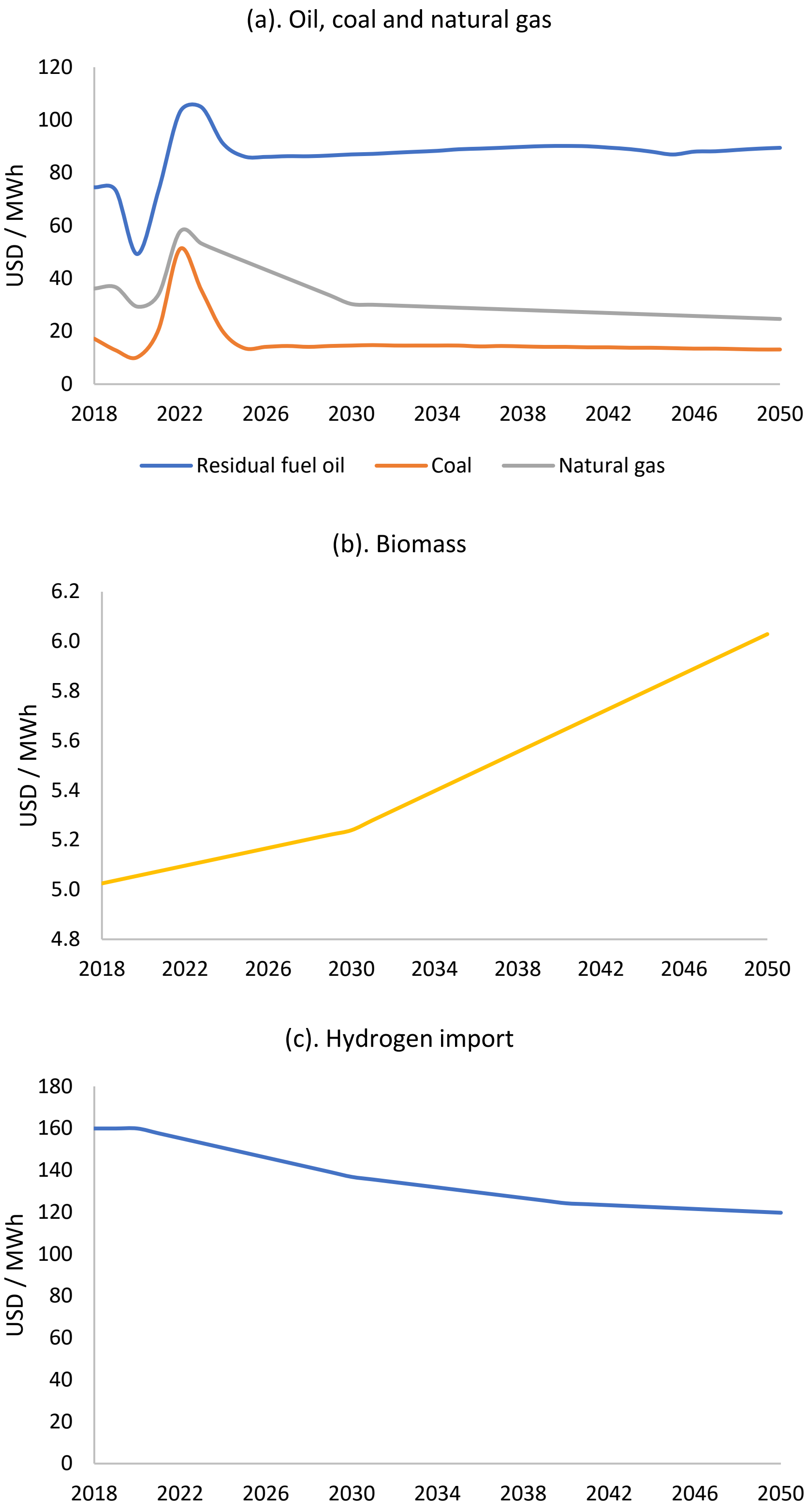

Figure S4. Trajectories of fuel prices

8 Capacity factors of hydro, solar and wind

Zhong, Yang [2] provide annual capacity factors for hydro, solar, and onshore wind. For offshore wind, the annual capacity factors are estimated using a simulation tool [63], following the approach in the IEA [85]. In the current analysis, hourly capacity factors of those renewables are required.

For hydro, we first collect monthly rainfall data [59] and then assign all 12 months to two even-sized groups based on the rainfall data, i.e., rainy season and dry season. The monthly hydro capacity factor for each group is based on the group-level average rainfall and is rescaled to match annual hydro capacity factors (Table S5). Hourly hydro capacity factors are assumed to be unchanged within the same month.

Table S5. Monthly hydro capacity factors (%)

Country /Month	1	2	3	4	5	6	7	8	9	10	11	12	Annual capacity factor
Brunei	0.00	0.00	0.00	0.00	0.00	0.00	0.00	0.00	0.00	0.00	0.00	0.00	0.00
Cambodia	12.85	12.85	12.85	12.85	69.15	69.15	69.15	69.15	69.15	69.15	12.85	12.85	41.00
Indonesia	53.57	53.57	53.57	53.57	37.43	37.43	37.43	37.43	37.43	37.43	53.57	53.57	45.50
LAO PDR	16.44	16.44	16.44	16.44	75.56	75.56	75.56	75.56	75.56	75.56	16.44	16.44	46.00
Malaysia	56.81	41.19	41.19	56.81	56.81	41.19	41.19	41.19	41.19	56.81	56.81	56.81	49.00
Myanmar	7.28	7.28	7.28	7.28	78.72	78.72	78.72	78.72	78.72	78.72	7.28	7.28	43.00
Philippines	21.96	21.96	21.96	21.96	21.96	44.04	44.04	44.04	44.04	44.04	44.04	21.96	33.00
Singapore	0.00	0.00	0.00	0.00	0.00	0.00	0.00	0.00	0.00	0.00	0.00	0.00	0.00
Thailand	12.58	12.58	12.58	12.58	57.42	57.42	57.42	57.42	57.42	57.42	12.58	12.58	35.00
Vietnam	24.92	24.92	24.92	24.92	24.92	83.08	83.08	83.08	83.08	83.08	83.08	24.92	54.00

To construct the dataset of hourly solar capacity factors, first, we collect hourly solar irradiance data for each country using NASA [60]. For a given country, we then assign all hours of a year to four even-sized groups based on their solar irradiance values (i.e., 0 - 25%, 25% - 50%, 50% - 75% and 75% - 100% quantiles). The hours in the same group would be assigned an identical solar capacity factor. Solar capacity of a group is proportional to the average hourly solar irradiance of that group and rescaled to match that country's annual solar capacity factor in Figure S5(a). We further assume improvements in annual solar capacity factor over years.

For onshore wind, we have collected wind speed data (at 50 meters) from NASA [60] for those countries with wind resources. For a given hour, we then estimate the onshore wind capacity factor by simulating the electricity output of an assumed typical wind turbine. This is done based on the relationship between the wind speed and the characteristics of the wind turbine [61]. Such an assumed wind turbine has a capacity of 900 kW and a diameter of 52.73 meters [62]. All hourly onshore wind capacity factors are then rescaled in accordance with the country’s annual onshore wind capacity factor in Figure S5(b). Onshore wind capacity factors are assumed to be stable over time.

For offshore wind, the hourly capacity factors in Figure S5(c) are generated using a simulation tool developed by Pfenninger and Staffell [63]. This is the major tool used in the IEA Offshore Wind Outlook 2019 [64]. This simulation tool can only provide results based on historical data. Following the findings in IEA [64], annual capacity factors of offshore wind are assumed to improve by 1% annually over time.

(a). Hourly capacity factors of solar in 2018

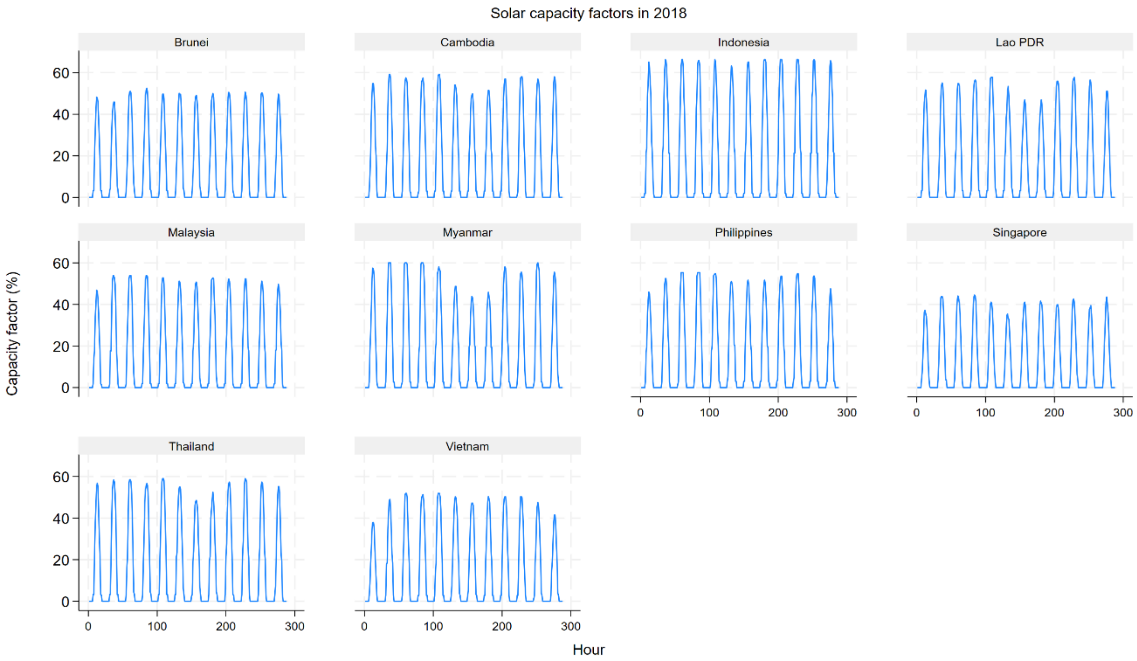

(b). Hourly capacity factors of onshore wind

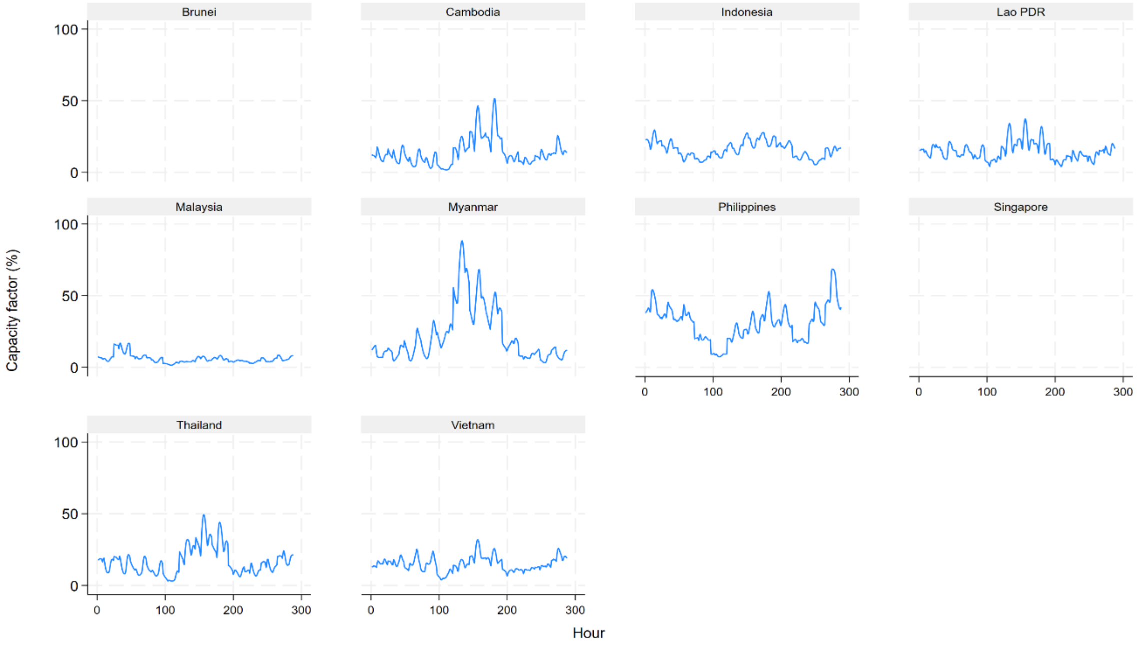

(c). Hourly capacity factors of offshore wind in 2018

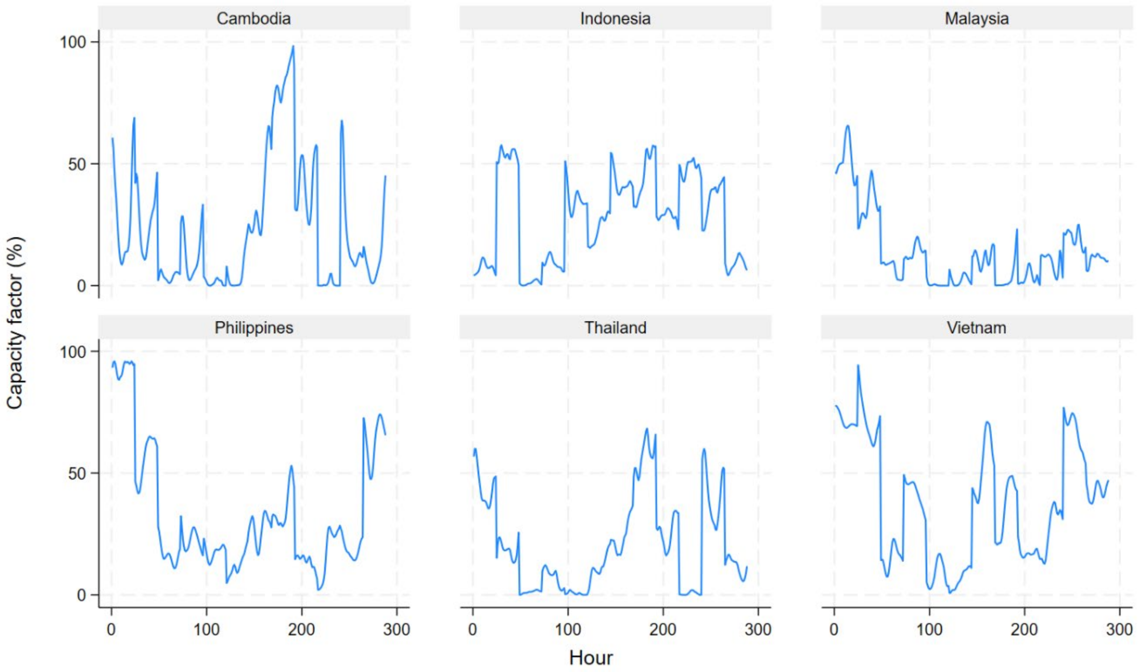

Figure S5. Hourly solar capacity factors in 2018 (%)

Note that hourly capacity factors of onshore wind are assumed to be unchanged over time. The capacity factors of solar and offshore wind differ across modelling years.

We use a "PVGIS" simulation tool developed by the Joint Research Centre of the European Commission [65]. This tool can simulate the annual electricity output for an assumed solar PV generation process with various features, such as capacity (e.g., 1 kWp), geographical locations, PV cell technology (e.g., crystalline silicon, CIS and CdTe) and tracking option (e.g., optimal slope). We have run multiple simulations for each country by using different combinations of those features. The maximum annual capacity factor among all simulations is used as the annual solar capacity factor in the long run in Figure S6(a).

For offshore wind, the simulation tool developed by Pfenninger and Staffell [63] does not cover future years. In Figure S6 (b), we assume that the annual capacity factor will increase by 1% annually through 2050. This is consistent with the historical trends in the IEA [64]. Hourly offshore wind capacity factors for 2020 are adjusted based on the projected annual offshore wind capacity factors.

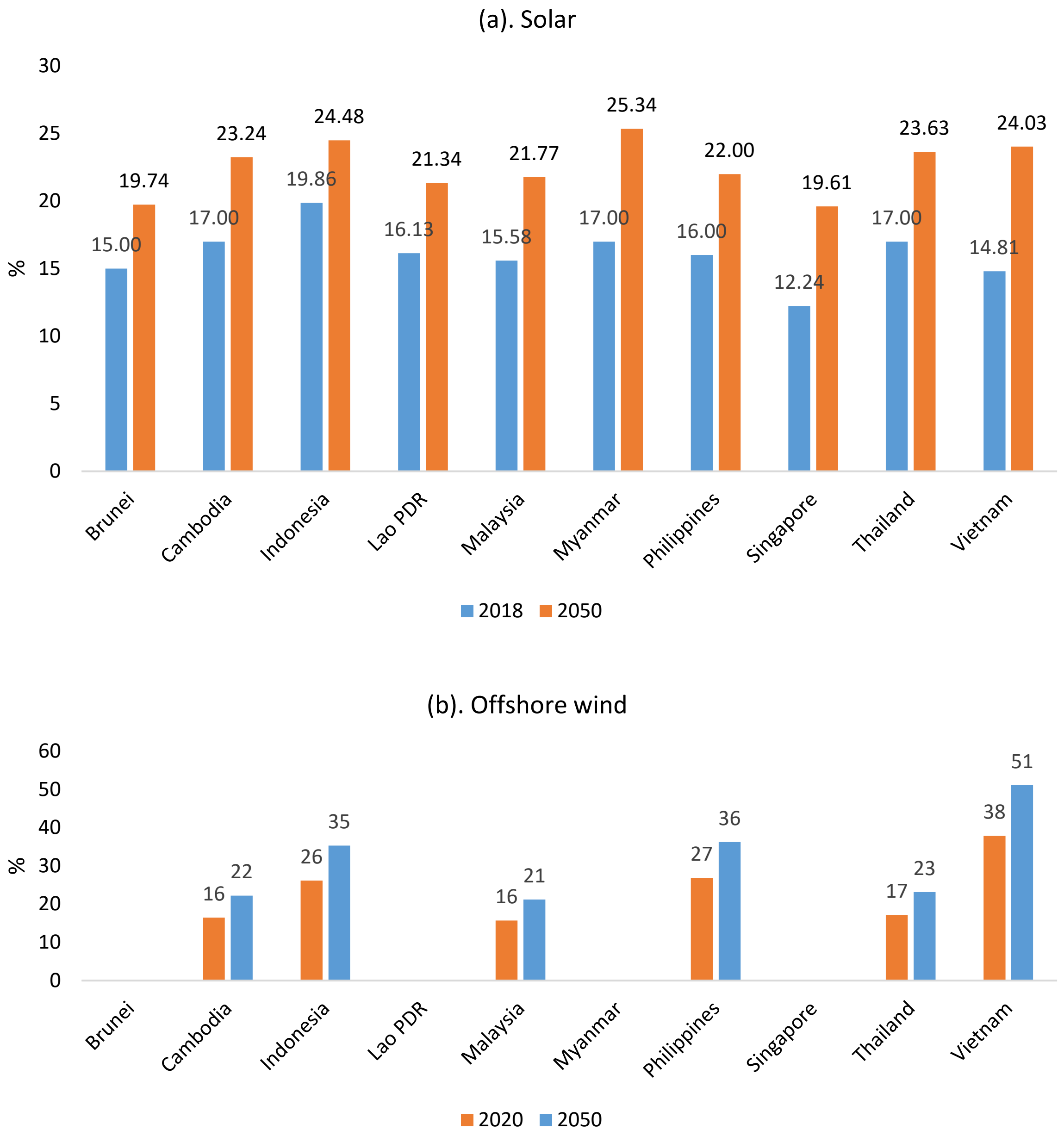

Figure S6. Trajectories of annual capacity factors of solar and offshore wind (%)

9 ASEAN's projected hourly dispatch in electricity generation in 2030 and 2050

(a). 2030

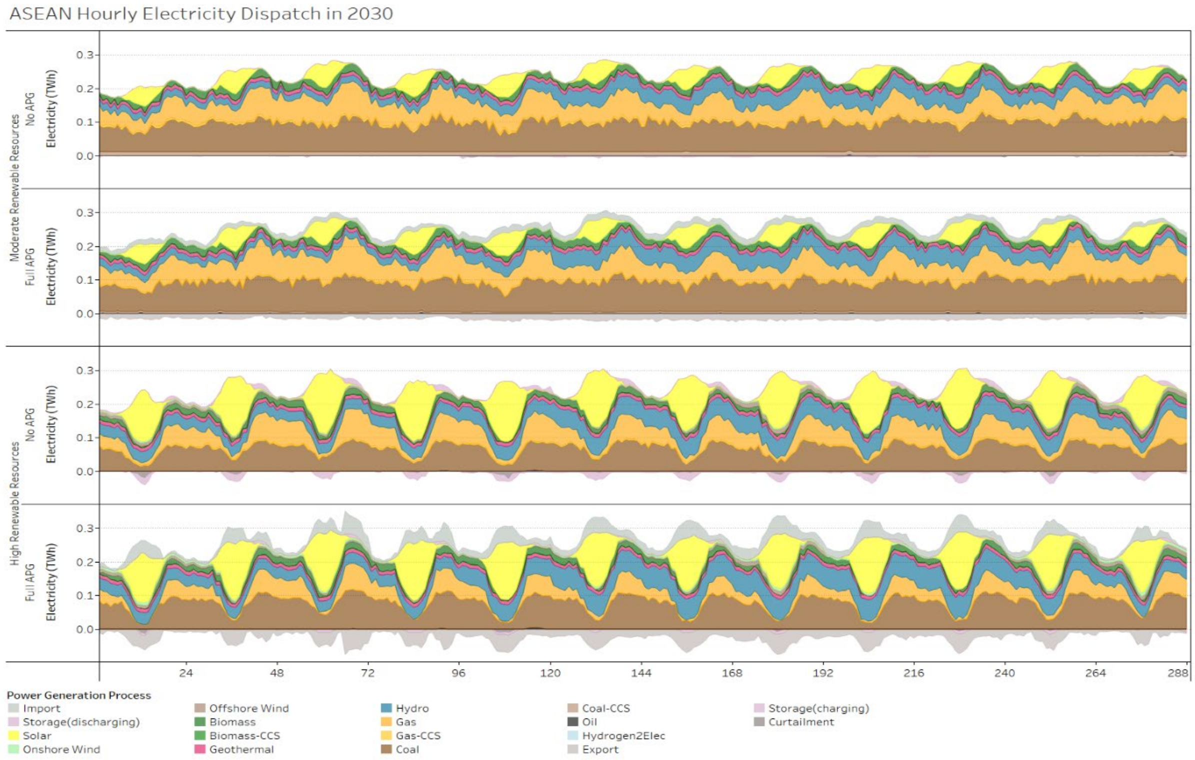

(b). 2050

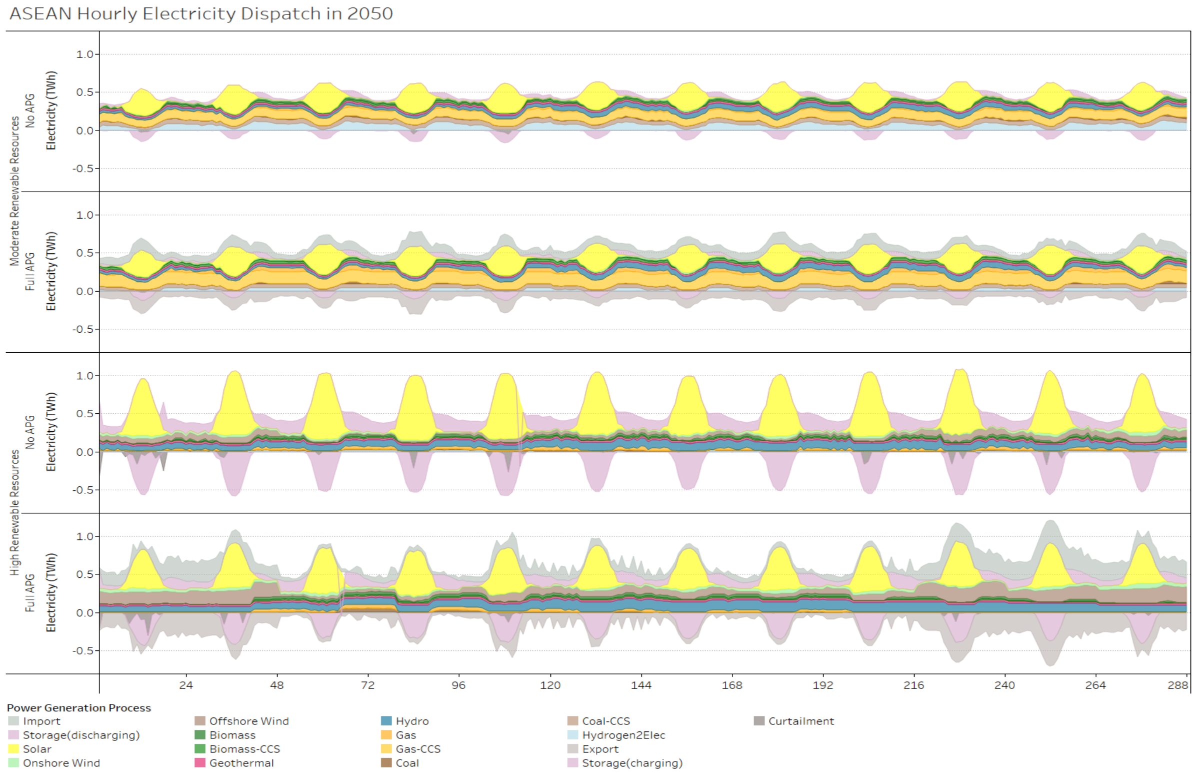

Figure S7. ASEAN's projected 2030 and 2050 hourly dispatch in electricity generation

Note that this figure shows the hourly dispatch in electricity generation for ASEAN (288 hours and aggregate results over all countries).

10 Country-specific results of electricity generation and hydrogen production

(a) Projected annual electricity generation in 2050

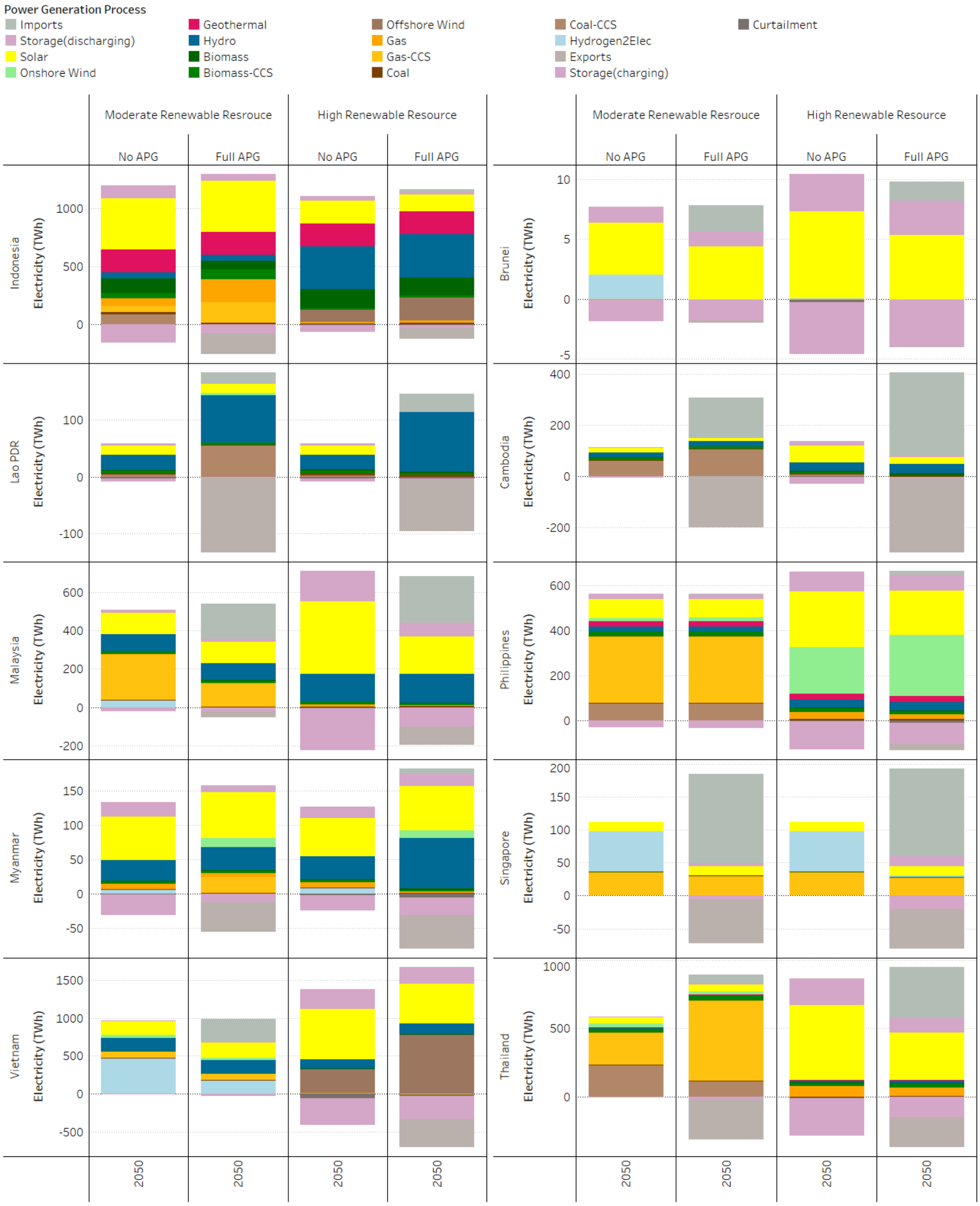

(b) Projected annual hydrogen production in 2050

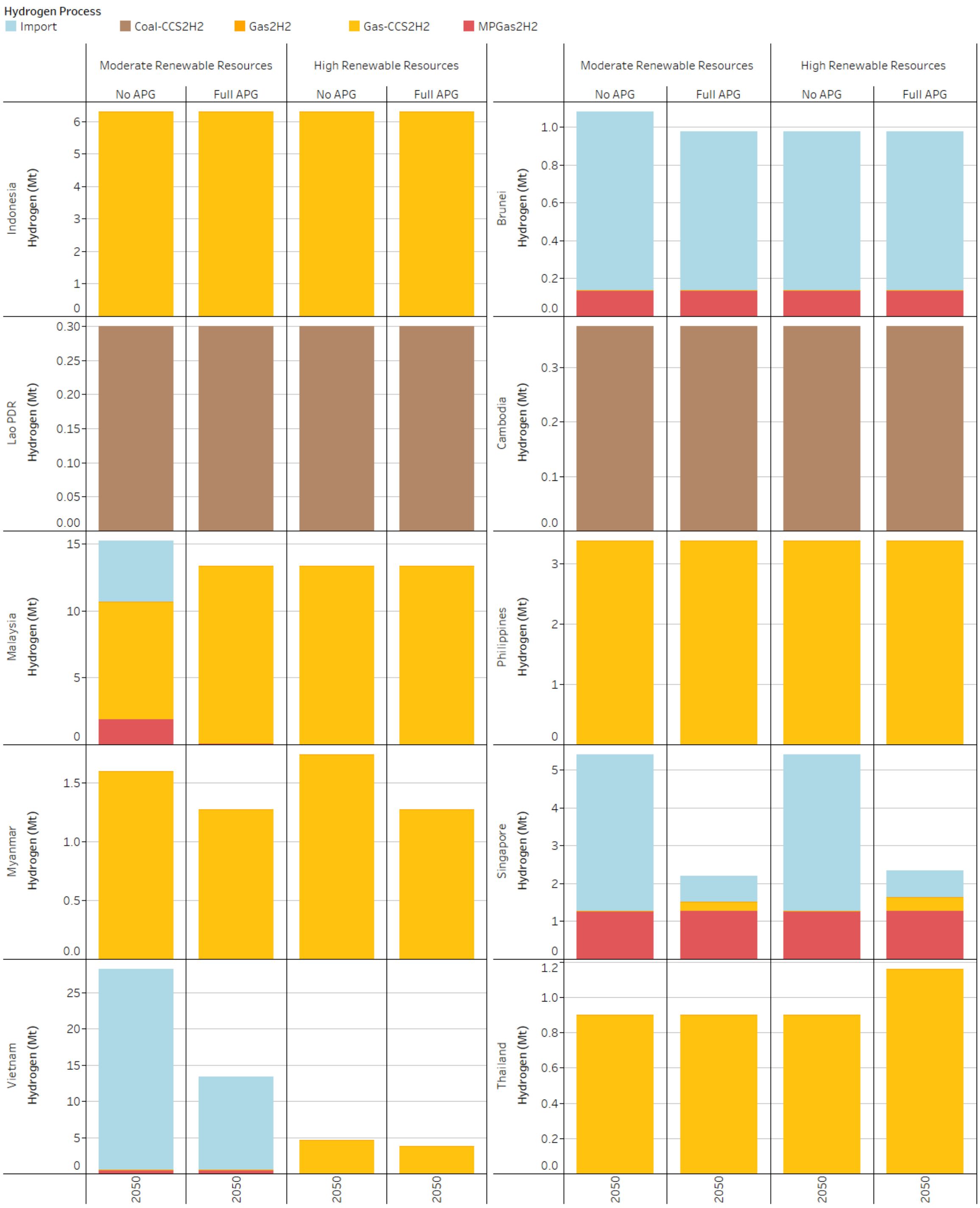

Figure S8. Projected electricity generation and hydrogen production by country, 2050

11 Sensitivity test results

This section presents the results of sensitivity tests. We decrease (-20% and -10%) and increase (10% and 20%) various key parameters of the model, including coal price, natural gas price, battery cost, VRE cost, CCS cost, transmission line cost, electricity demand, and hydrogen demand. Such changes are applied to all four base scenarios presented in the manuscript. Section 11.1 – Section 11.9 below provide the test results.

Key findings from the sensitivity tests are the following.

11.1 Total system costs from sensitivity tests

Figure S9 presents the total system cost over all modelling years by scenario. Across all scenarios, a larger increase in the key parameter (i.e., fossil fuel price, technology cost or demand) would lead to a larger energy system cost. Reducing the key parameter can lower total energy system cost. Changes in the electricity demand have the largest impact on the energy system cost, whereas the impacts of changing other key parameters tend to be much smaller.

Further, we calculate the difference in total energy system costs between the scenarios with Full APG and those with No APG. It is evident that cross-border transmission can lower system cost (Figure S10). This is because more cost-effective supply options would be available through the connected networks. With a larger increase in electricity demand, gas price or battery storage cost, the benefit (in terms of the reduction in total energy system cost) arising from APG becomes larger. If the cost of transmission increases, the benefit due to APG would slightly drop.

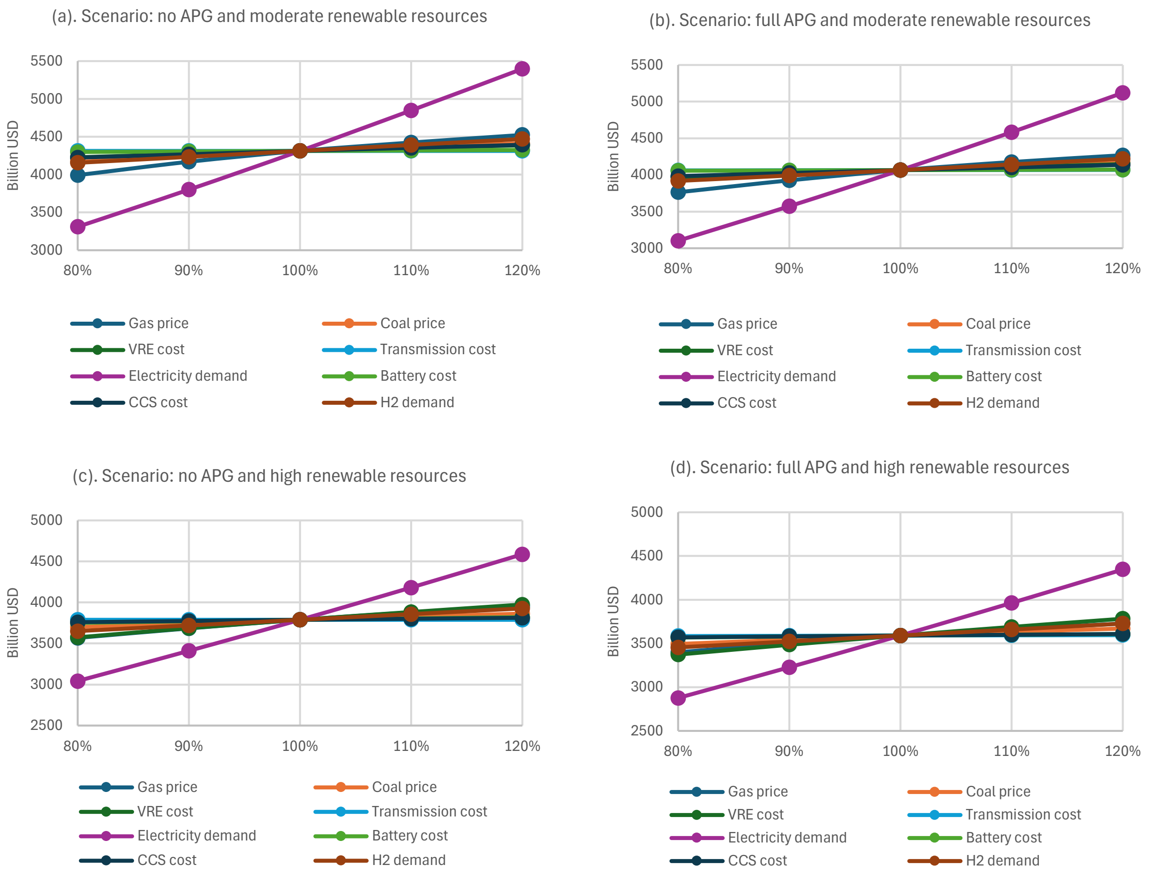

Figure S9. Total system costs of sensitivity tests by scenario

Note that "100%" on the X axis indicates the base scenario results. "80%", "90", "110%" and "120%" on the X axis indicate a 20% reduction, a 10% reduction, a 10% increase and a 20% increase, respectively.

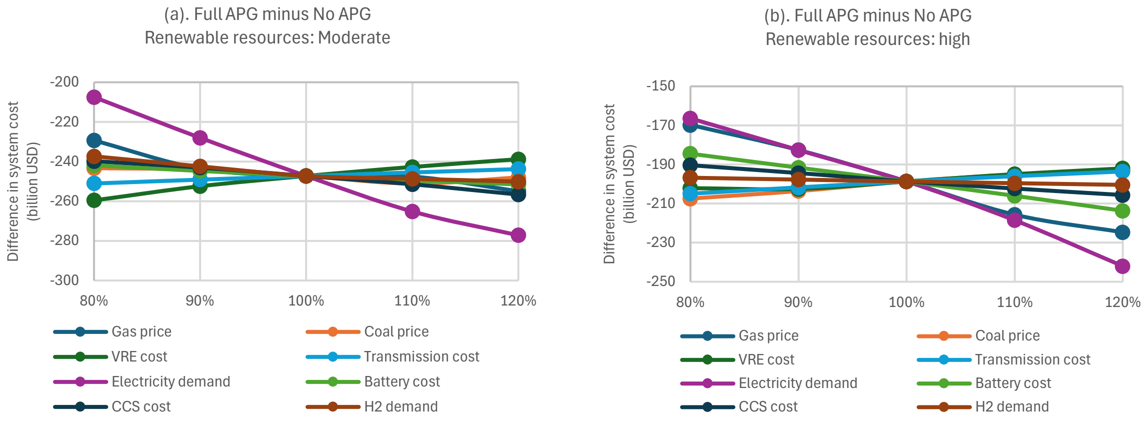

Figure S10. Differences in total energy system costs between Full APG and No APG

Note that "100%" on the X axis indicates the base scenario results. "80%", "90", "110%" and "120%" on the X axis indicate a 20% reduction, a 10% reduction, a 10% increase and a 20% increase, respectively.

11.2 Sensitivity tests of electricity demand

In this section we apply a 20% reduction, a 10% reduction, a 10% increase and a 20% increase in the electricity demand. This applies to all countries and modelling years. Figure S11 presents the results of ASEAN's annual electricity generation and hydrogen production.

The two "bookend" pathways of electricity generation remain consistent (Panel a of Figure S11). With moderate renewable resource potentials, renewable potentials are restricted below the technical levels. In such cases, there would be an expansion in emerging low-carbon generation, i.e., natural gas with CCS (replacing coal-fired electricity) and hydrogen-to-electricity, to meet the rising electricity demand. The share of those low-carbon generation under Full APG is smaller than that under No APG. If electricity demand drops, the share of CCS and hydrogen-to-electricity in generation mix would decrease. With high renewable resource potentials, there would be an expansion in VRE and battery storage (i.e., solar and offshore wind) to meet the rising electricity demand. In all cases, the share of battery storage under Full APG is smaller than that under No APG.

For hydrogen production, result patterns remain consistent with those in base cases (Panel b of Figure S11). With moderate renewable resource potentials, rising electricity demand would increase the demand for hydrogen. The increasing hydrogen demand is projected to be met by low-carbon hydrogen import (e.g., the case with a 20% increase in electricity demand). With high renewable resource potentials, as there is little change in the hydrogen demand from power sector, the hydrogen production mix remains stable in the sensitivity tests.

(a) Effects of changing electricity demand on electricity generation

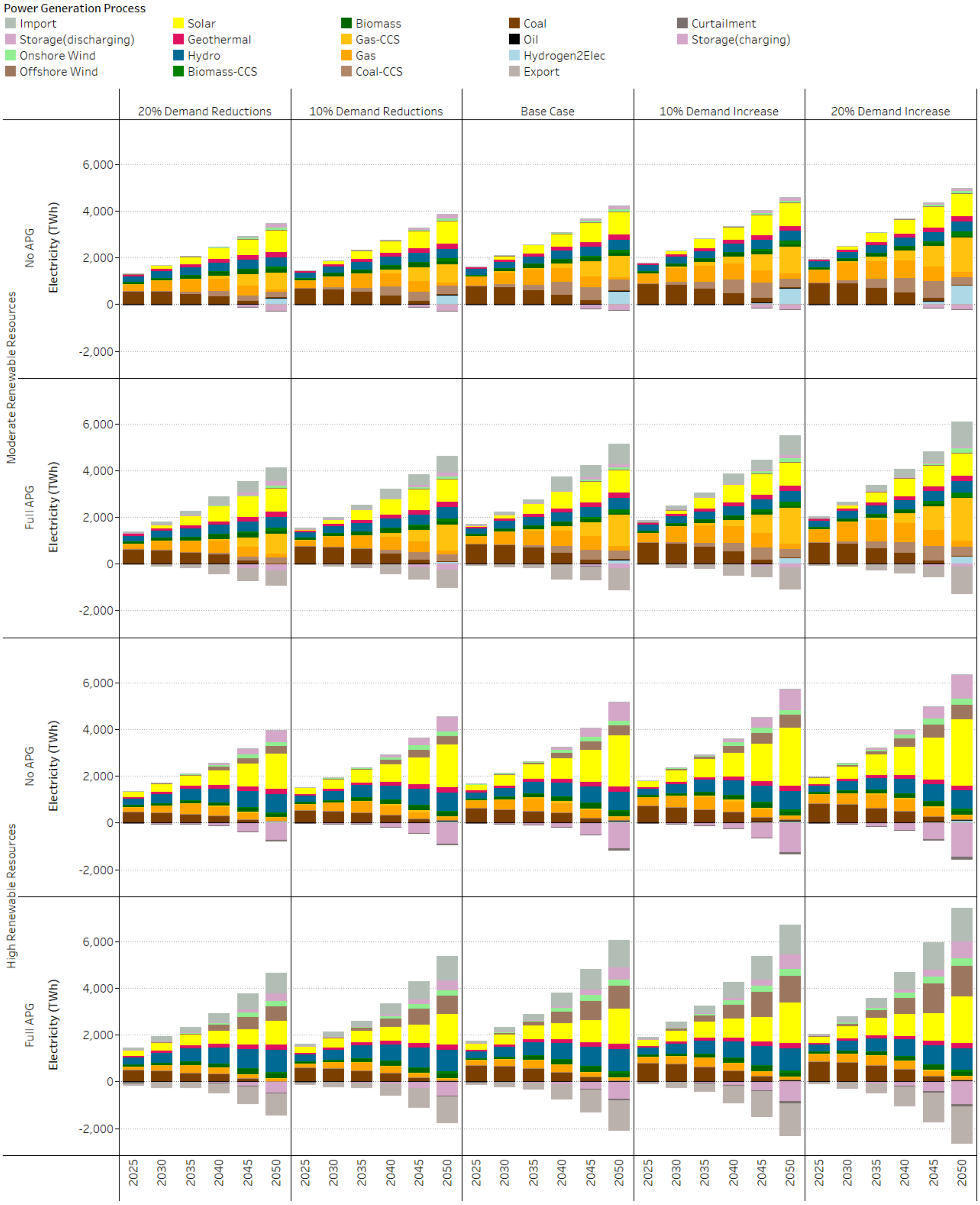

(b) Effects of changing electricity demand on hydrogen production

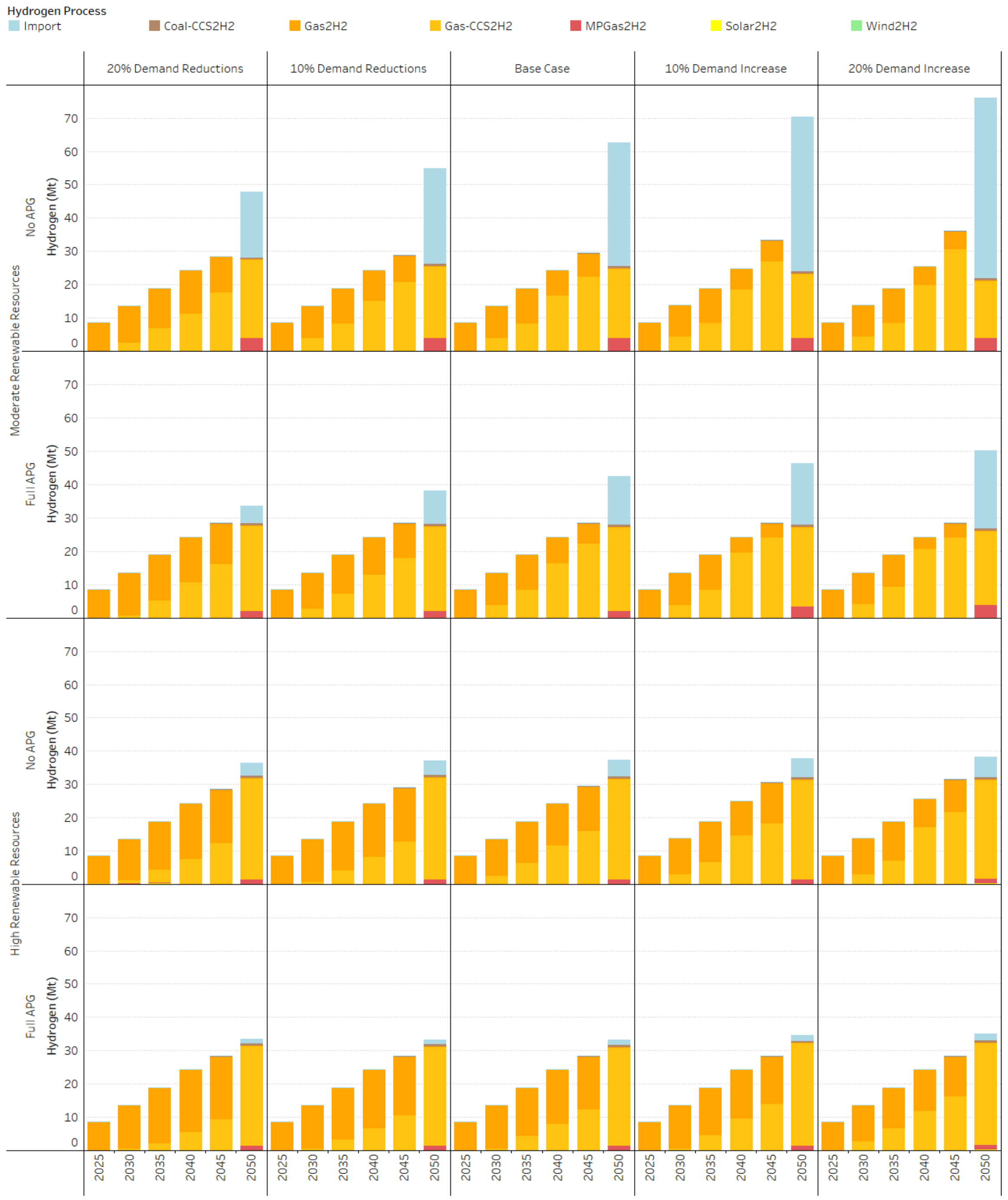

Figure S11. Effects of changing electricity demand on electricity generation and hydrogen production

11.3 Sensitivity tests of coal price

In this section, we apply a 20% reduction, a 10% reduction, a 10% increase and a 20% increase in the coal price for all countries and modelling years. Figure S12 shows the results of ASEAN's annual electricity generation and hydrogen production.

Overall, the electricity generation mix remains consistent across sensitivity test scenarios (Panel a of Figure S12). With moderate renewable resource potentials, there would be trade-offs between coal

with CCS and natural gas with CCS. if coal price increases, there would be a substitution of coal-fired power (with CCS) with natural gas with CCS, and thus a decline in coal with CCS in generation mix. If coal price decreases, the share of coal with CCS would become larger. With high renewable resource potentials, the 2050 generation mix remains relatively stable across sensitivity tests. However, there would be a faster phasing-out of coal-fired power in early years if coal price increases.

For hydrogen production, since coal with CCS only accounts for a marginal share in the production structure. Changes in coal price have litter impact on hydrogen production (Panel b of Figure S12).

(a) Effects of changing coal price on electricity generation

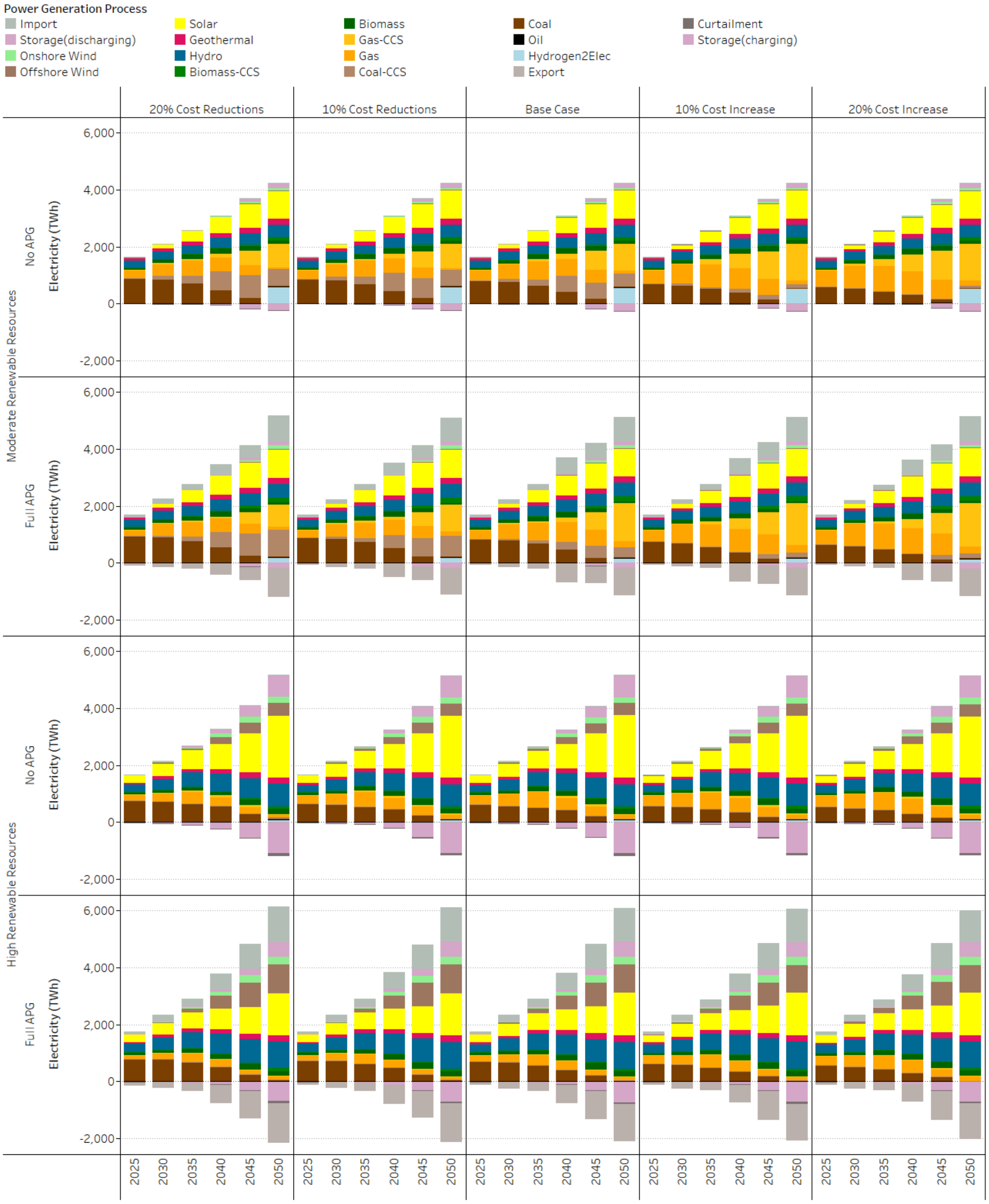

(b) Effects of changing coal price on hydrogen production

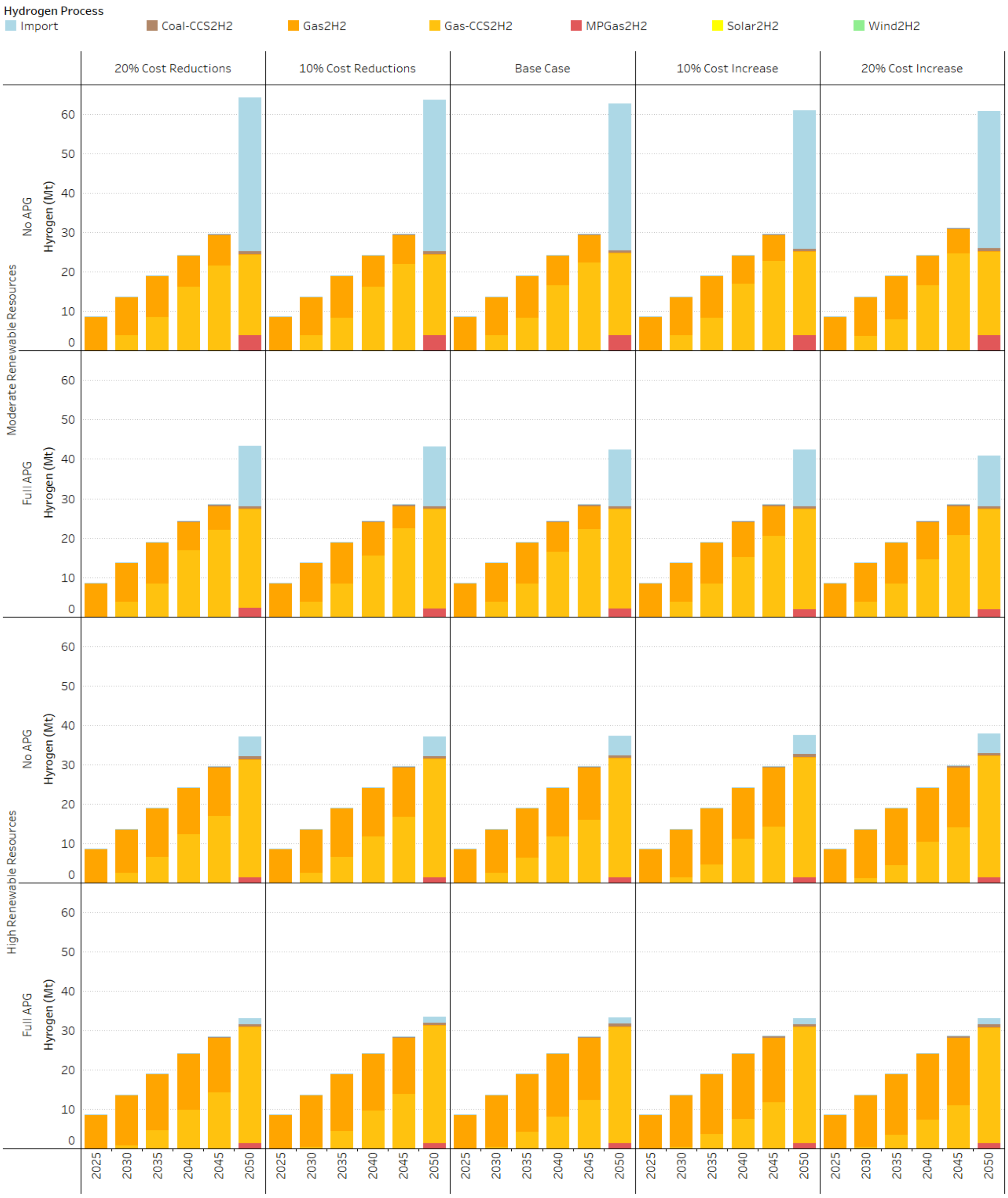

Figure S12. Effects of changing coal price on electricity generation and hydrogen production

11.4 Sensitivity tests of natural gas price

In this section we apply a 20% reduction, a 10% reduction, a 10% increase and a 20% increase in natural gas price for countries and modelling years. Figure S13 shows the sensitivity test results of the changes in natural gas price.

To summarize, there are no radical changes in the electricity generation mix by changing natural gas price (Panel a of Figure S13). With moderate renewable resource potentials, similarly, there would be trade-offs between coal with CCS and natural gas with CCS. If natural gas becomes more expensive,

coal with CCS would replace natural gas with CCS and thus the share of gas-fired power would decrease. Shares of other generation technologies remain relatively stable in the generation mix. With high renewable resource potentials, similar to the case of changing coal price, the 2050 generation mix remains stable, and there would be a faster substitution of gas-fired power with coal-fired power, if natural gas price increases.

Regarding the hydrogen production, methane pyrolysis (natural gas as the feedstock) will be phasing out from the production mix, if natural gas price increases (Panel b of Figure S13). With a larger decrease in natural gas price, there would be an expansion in this production technology. As changing natural gas price does not lead to significant changes in hydrogen from power sector, there is no large expansion in the hydrogen production in all sensitivity tests.

(a) Effects of changing natural gas price on electricity generation

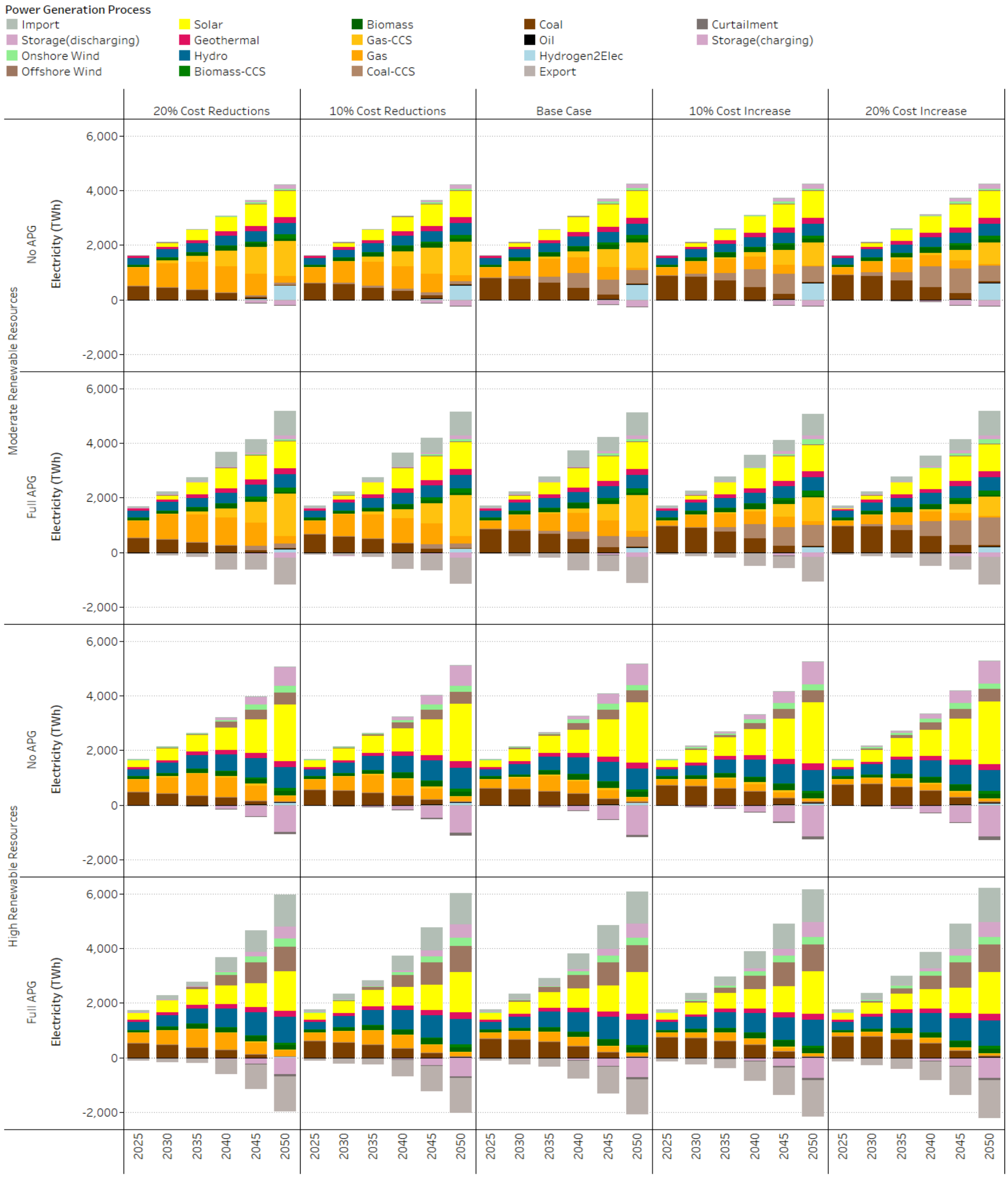

(b) Effects of changing natural gas price on hydrogen production

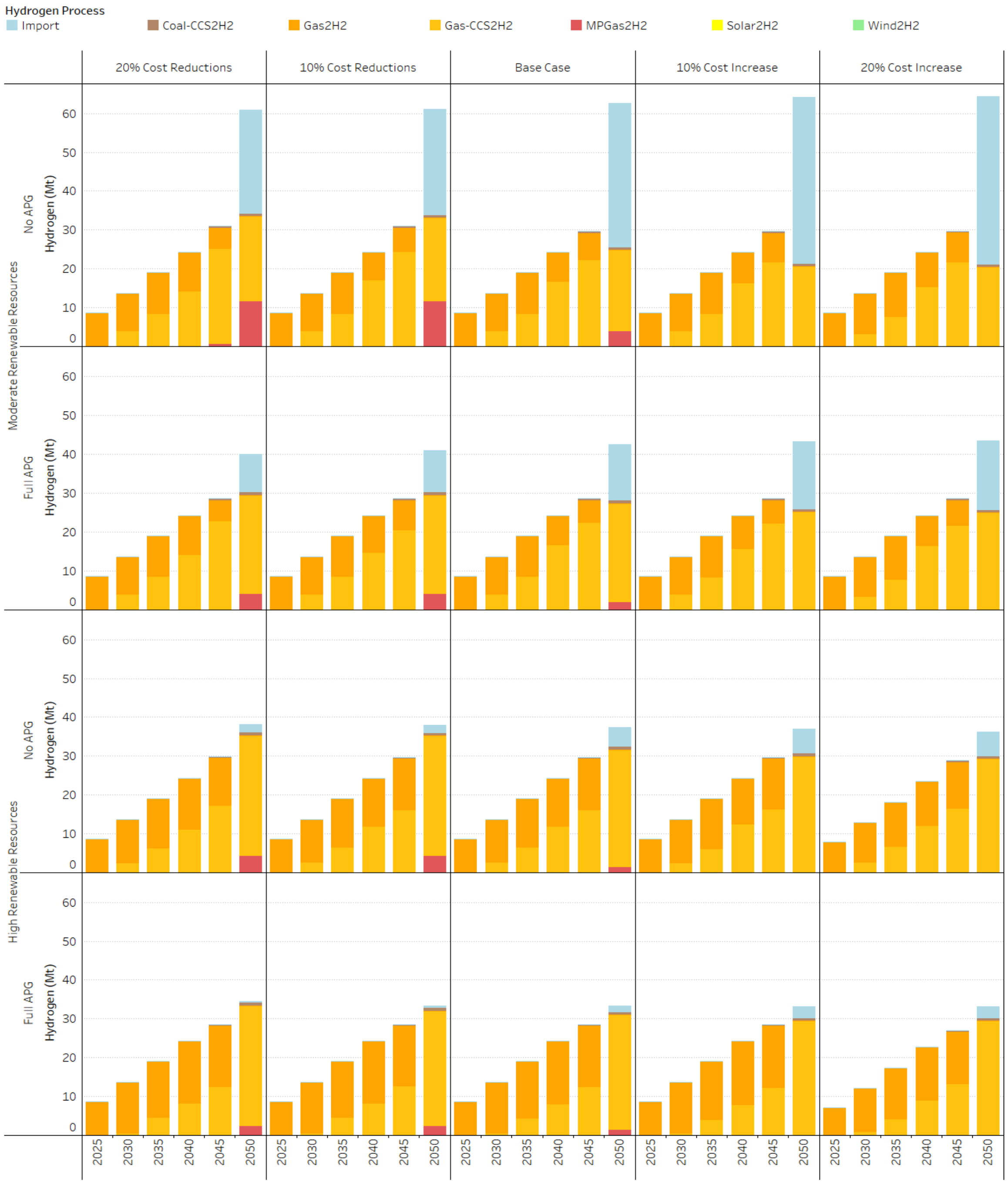

Figure S13. Effects of changing natural gas price on electricity generation and hydrogen production

11.5 Sensitivity tests of VRE cost

In this section we apply a 20% reduction, a 10% reduction, a 10% increase and a 20% increase in the CAPEX and fixed cost of VRE technologies (i.e., solar, onshore wind and offshore wind). In all base scenarios, we have already assumed cost reductions for those VRE technologies (see Figure S2). The cost changes in this section are additional to those assumed cost reductions in the base scenarios. Figure S14 presents the sensitivity test results of changes in VRE cost.

In general, the electricity generation mix is consistent across sensitivity tests (Panel a of Figure S14). With moderate renewable resource potentials, reducing VRE cost can lower the share of natural gas with CCS, and increase the share of onshore wind that is underutilization (e.g., up to a 5% increase in generation mix from the base case with Full APG and a 20% cost reduction). With high renewable resource potentials, high VRE costs would slightly lower the share of solar (replaced by hydro) and battery storage. On other hand, decrease in VRE costs can lead to expansions in VRE and battery storage (e.g., up to a 3% increase for solar in the No APG case).

Hydrogen production mix remains stable in all sensitivity tests, as there is no large change in the hydrogen demand. Current model assumes local hydrogen production and hydrogen import from outside ASEAN and does not consider hydrogen storage and transport between ASEAN countries. The ASEAN country with high hydrogen demand may not be the country with high VRE potentials. Reducing VRE cost can only increase the share of solar-to-hydrogen in production mix by up to 2%.

(a) Effects of changing VRE cost on electricity generation

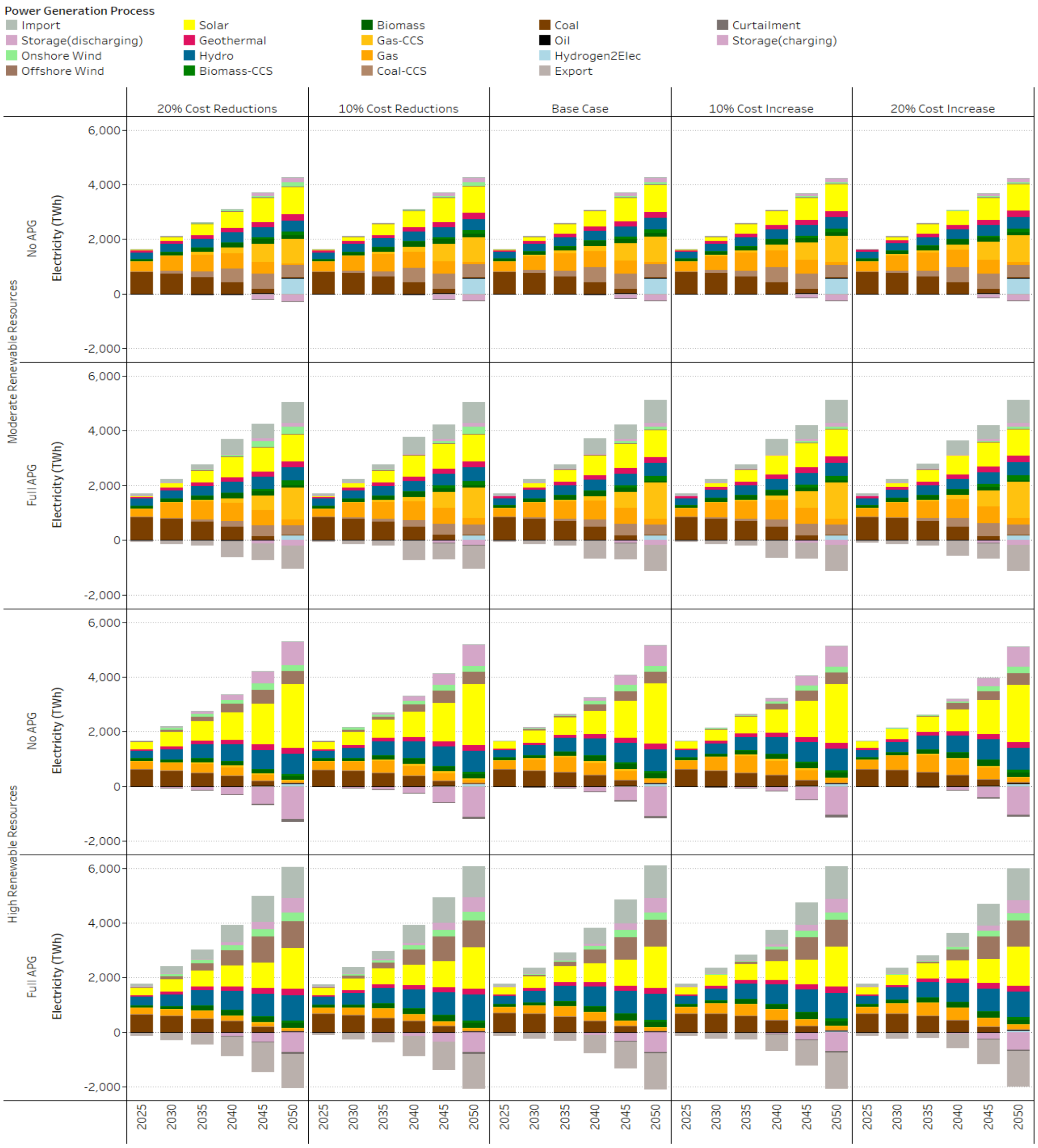

(b) Effects of changing VRE cost on hydrogen production

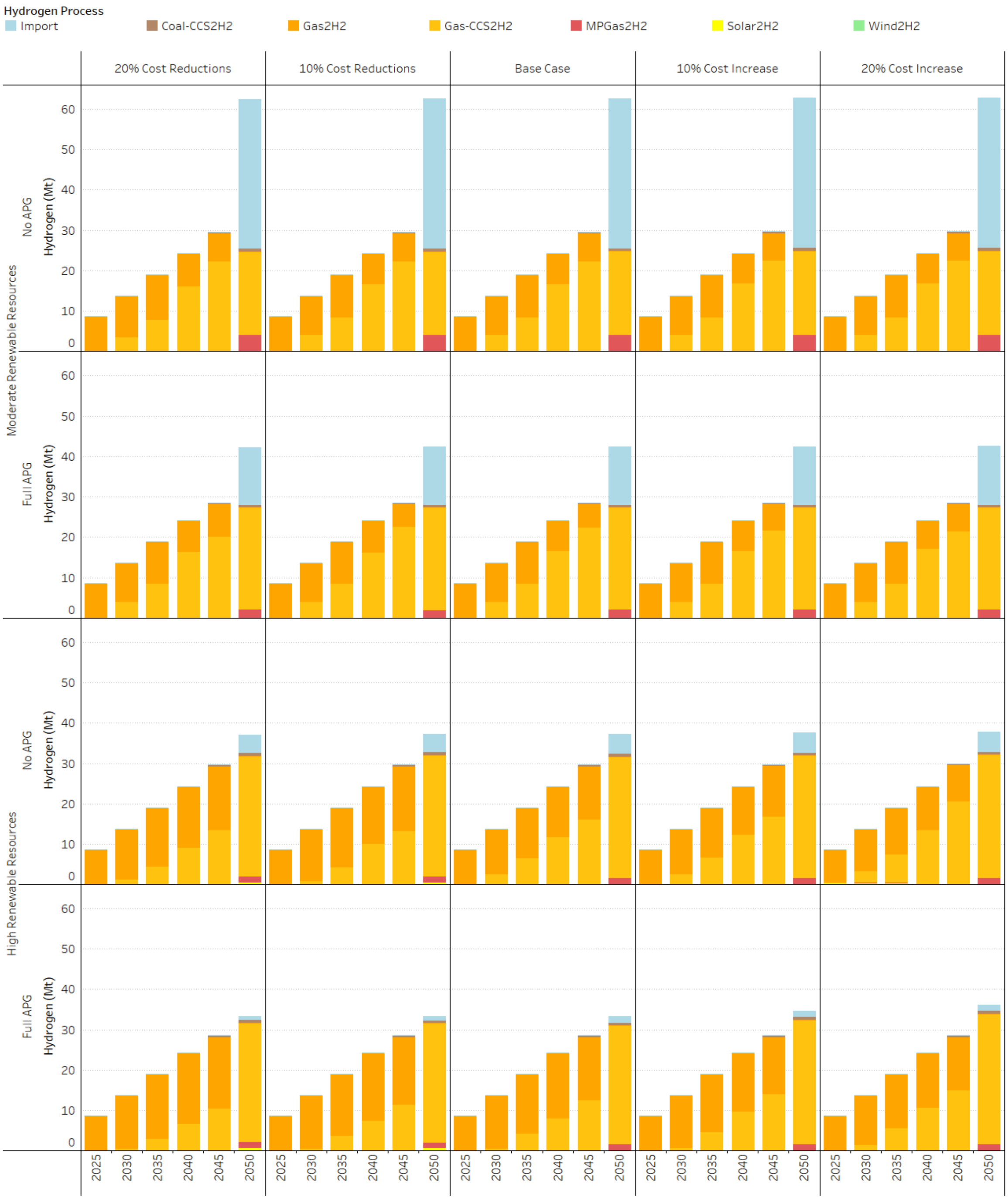

Figure S14. Effects of changing VRE cost on electricity generation and hydrogen production

11.6 Sensitivity tests of battery storage cost

In this section, we apply a 20% reduction, a 10% reduction, a 10% increase and a 20% increase in the CAPEX and fixed cost of battery storage. Such changes are additional to those battery cost reductions implemented in current model (see Figure S2). Figure S15 below presents the sensitivity test results of ASEAN's electricity generation and hydrogen production.

Overall, the trends in electricity generation are consistent (Panel a of Figure S15). An increase (or a decrease) in the battery storage cost can lead to a larger (or smaller) share of battery storage in electricity generation (e.g., up to about a 4% increase from the base scenario with No APG), regardless

of the levels of renewable resource potentials. In all tests, scenarios with Full APG have a smaller share of battery storage than those with No APG, because cross-border transmission can lower the demand for battery storage. This is consistent with the findings from the base scenarios.

When renewable resource potentials are restricted in the moderate case, lowering battery cost can lead to the substitution of coal-fired power with natural gas with CCS. With high renewable resource potentials, low-cost battery storage can promote the expansion in domestic solar and thus lower the demand for electricity import and export.

For hydrogen production, as the changes in battery storage do not lead to significant changes in hydrogen demand for electricity generation, the hydrogen production mix remains stable across all sensitivity tests (Panel b of Figure S15).

(a) Effects of changing battery storage cost on electricity generation

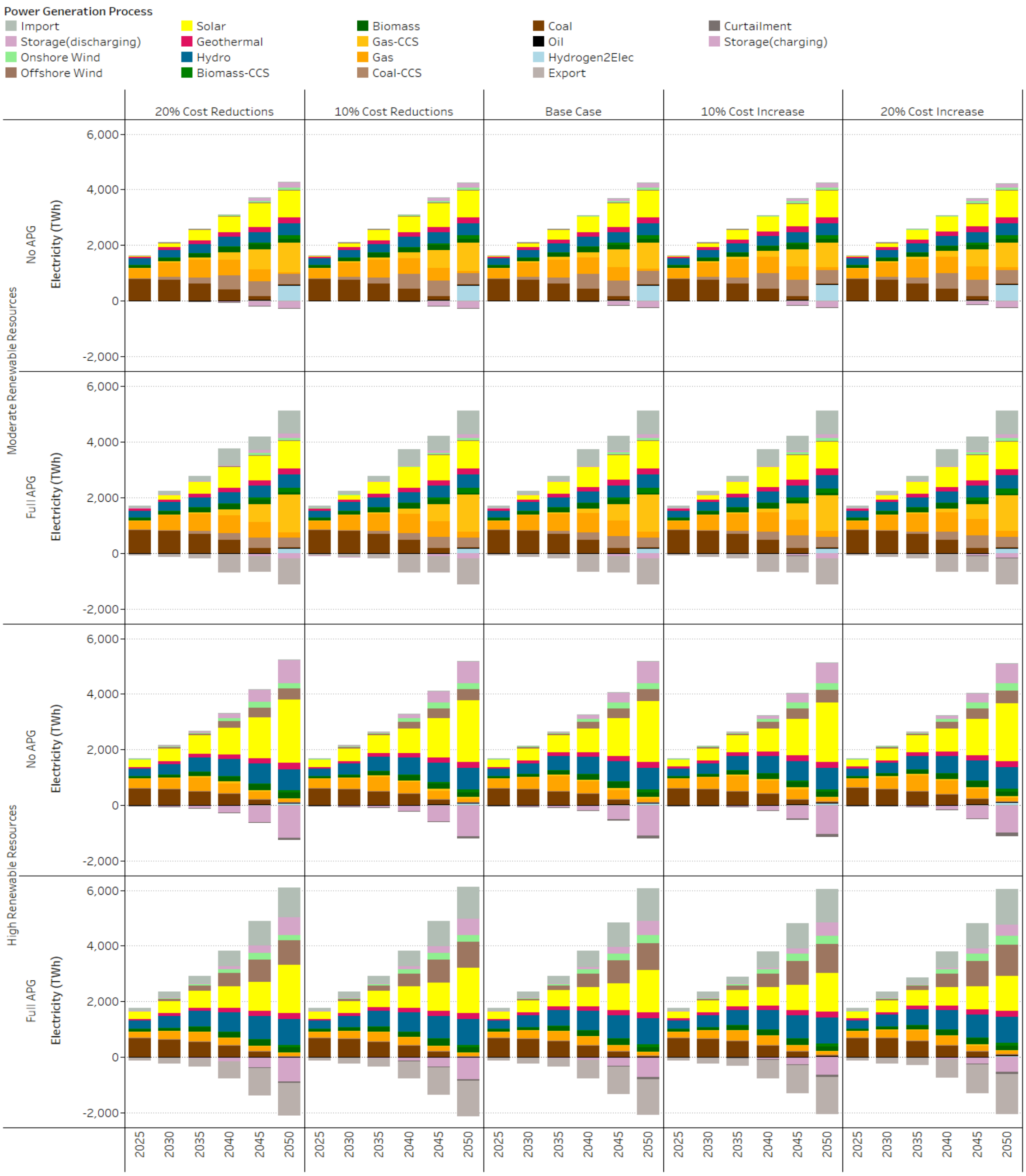

(b) Effects of changing battery storage cost on hydrogen production

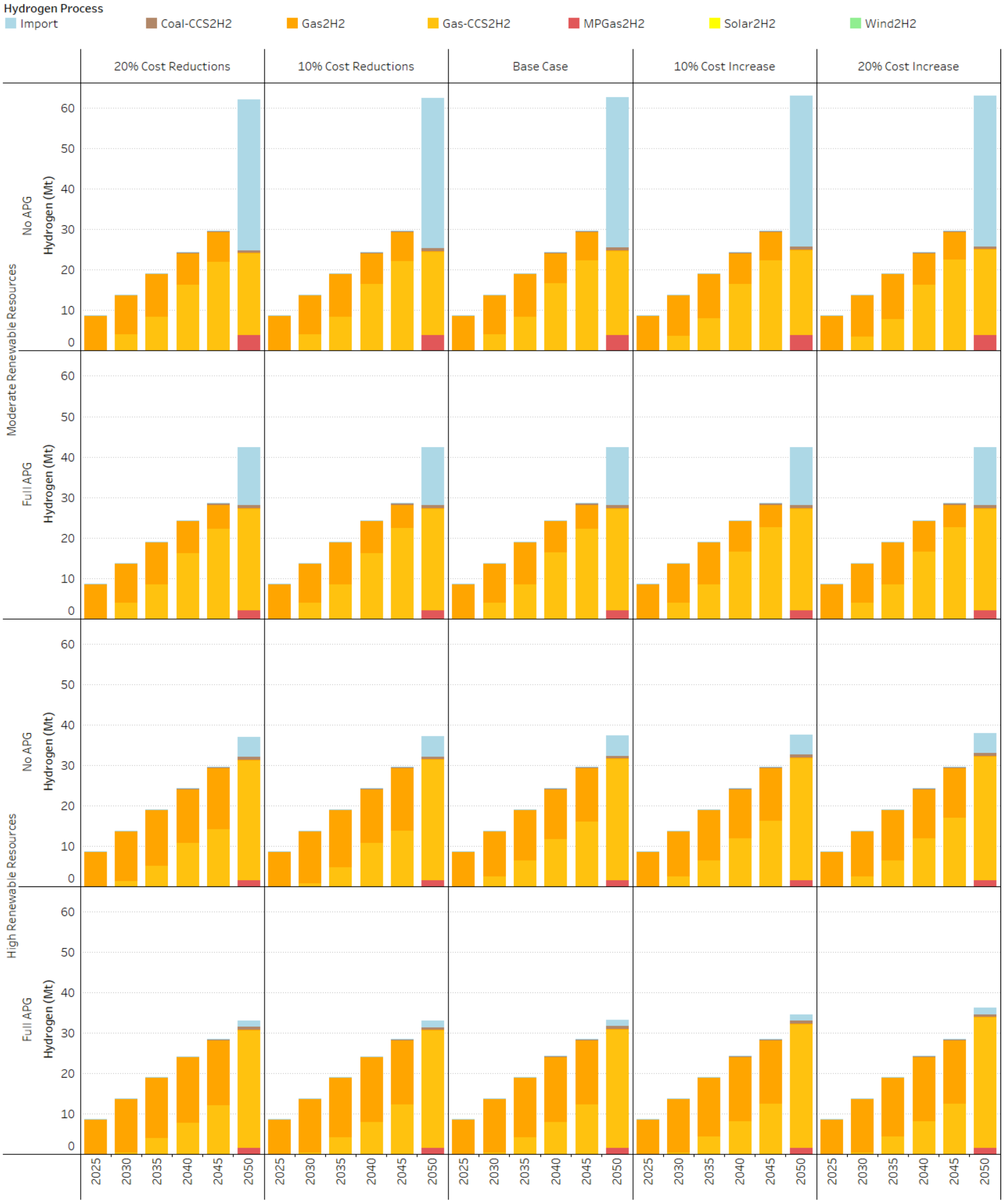

Figure S15. Effects of changing battery storage cost on electricity generation and hydrogen production

11.7 Sensitivity tests of CCS cost

In this section we apply a 20% reduction, a 10% reduction, a 10% increase and a 20% increase in the CAPEX and fixed cost of all CCS technologies. All changes are additional to the cost reductions of CCS implemented in current model (Figure S2). Figure S16 shows the sensitivity test results.

Overall, the electricity generation results in sensitivity tests are in line with those in the base scenarios (Panel a of Figure S16). With moderate renewable resource potentials, fossil fuel-based CCS can play a

role. Under current sensitivity test settings, increasing (or decreasing) the costs of CCS can lead to a larger (or smaller) share of CCS in generation mix, but only marginally (changes in generation mix up to about 2%). This is because there are limited decarbonisation options with moderate renewable potentials, and CCS is still cost-competitive compared to hydrogen. As shown in Figure 3 in the manuscript, for hydrogen to be a viable option in power sector, significant cost reduction (more than 60%) would be needed. If high renewable resource potentials are available, CCS accounts for a marginal share in generation. Changing CCS costs under current settings has little impact on the electricity generation.

For hydrogen production, as there is no significant change in hydrogen demand from power sector, hydrogen production structure remains stable (Panel b of Figure S16).

(a) Effects of changing CCS cost on electricity generation

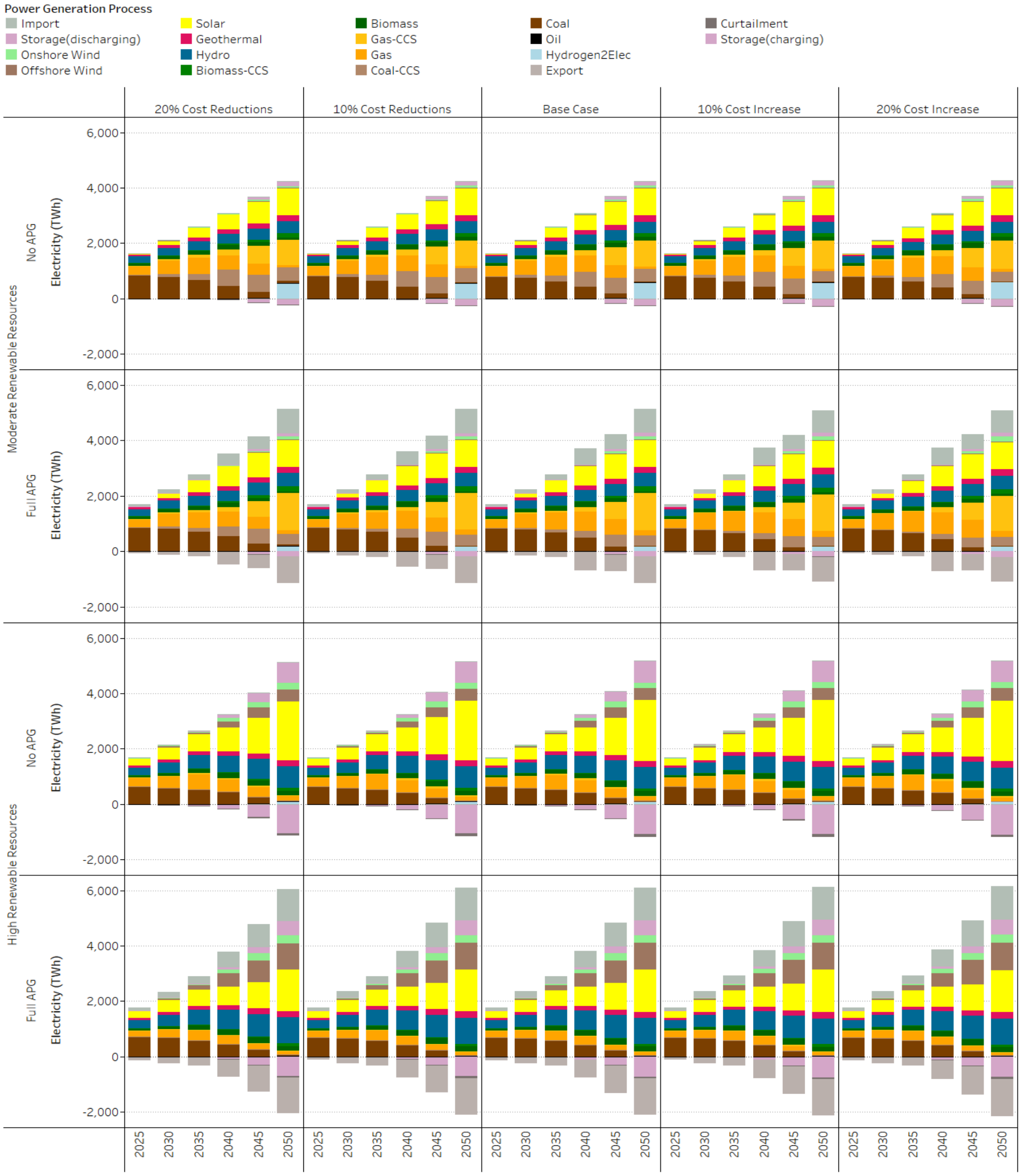

(b) Effects of changing CCS cost on hydrogen production

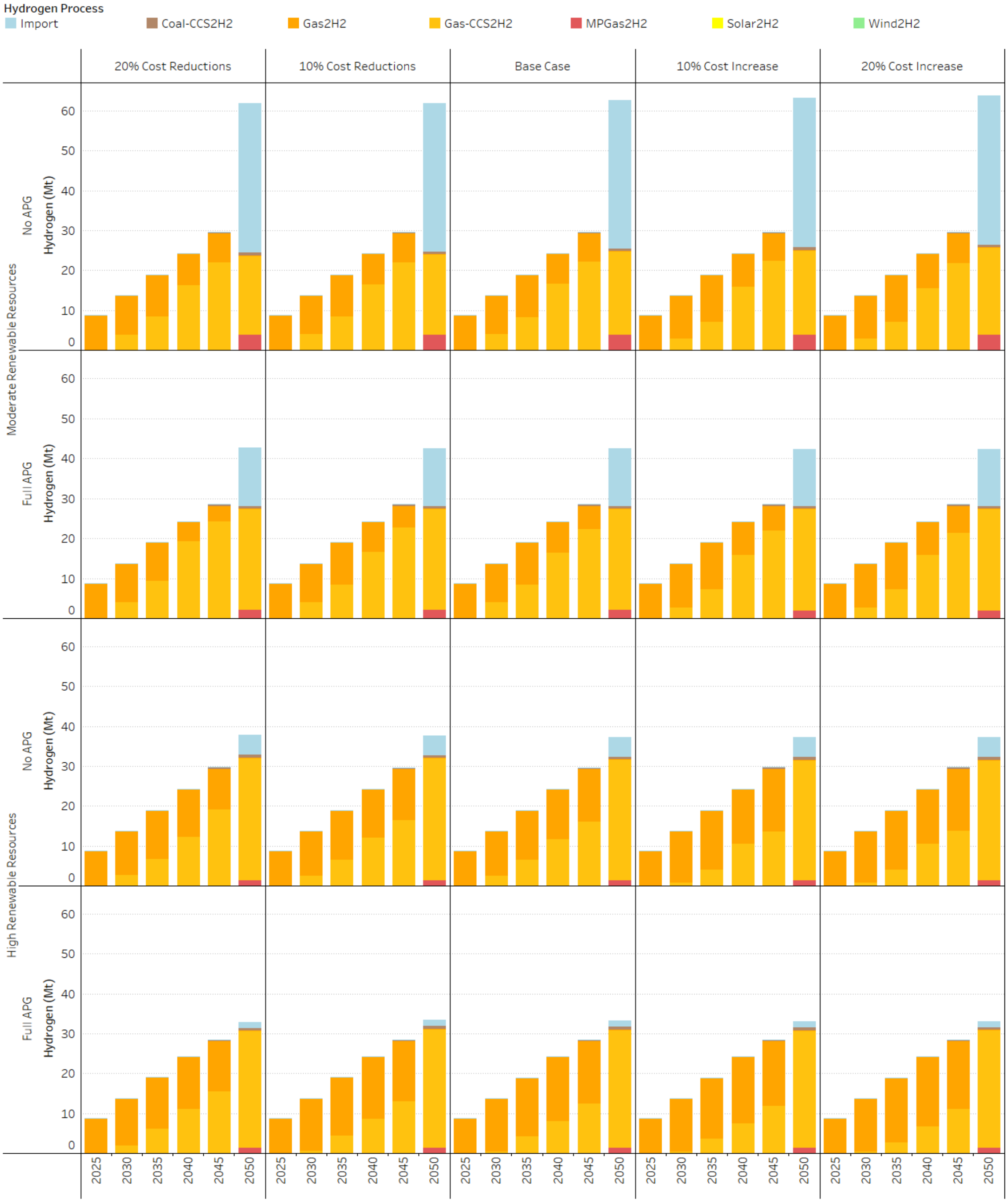

Figure S16. Effects of changing CCS cost on electricity generation and hydrogen production

11.8 Sensitivity tests of transmission cost

In this section, we apply a 20% reduction, a 10% reduction, a 10% increase, and a 20% increase in the CAPEX and fixed cost of transmission technologies (HV onshore and HV offshore, see Table S2). Figure S17 shows the sensitivity test results.

To summarize, both electricity generation mix and hydrogen production mix remain quite stable. For the scenario with No APG, changing the transmission line cost has no impact on the electricity generation and hydrogen production, as cross-border transmission is prohibited. For those scenarios

with Full APG, this is largely due to the upper limits of electricity imports we set in the current model (see Section 5 of this document). Lowering transmission cost can only increase electricity exports by about 1% with high renewable resource potentials (restricted by countries' electricity import upper limits). There is no significant change in hydrogen from power sector. Changes in transmission cost has little impact on hydrogen production mix.

(a) Effects of changing transmission cost on electricity generation

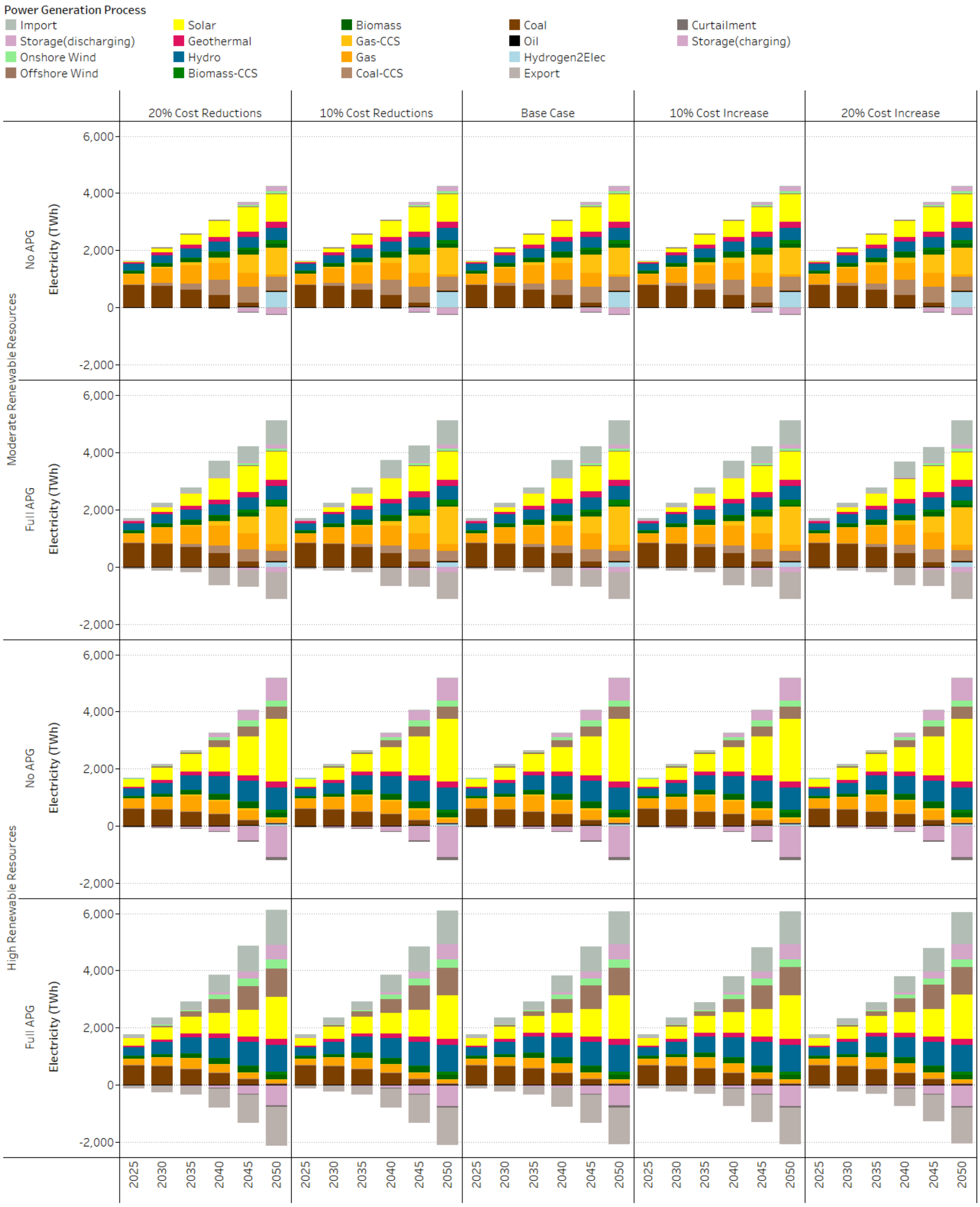

(b) Effects of changing transmission cost on hydrogen production

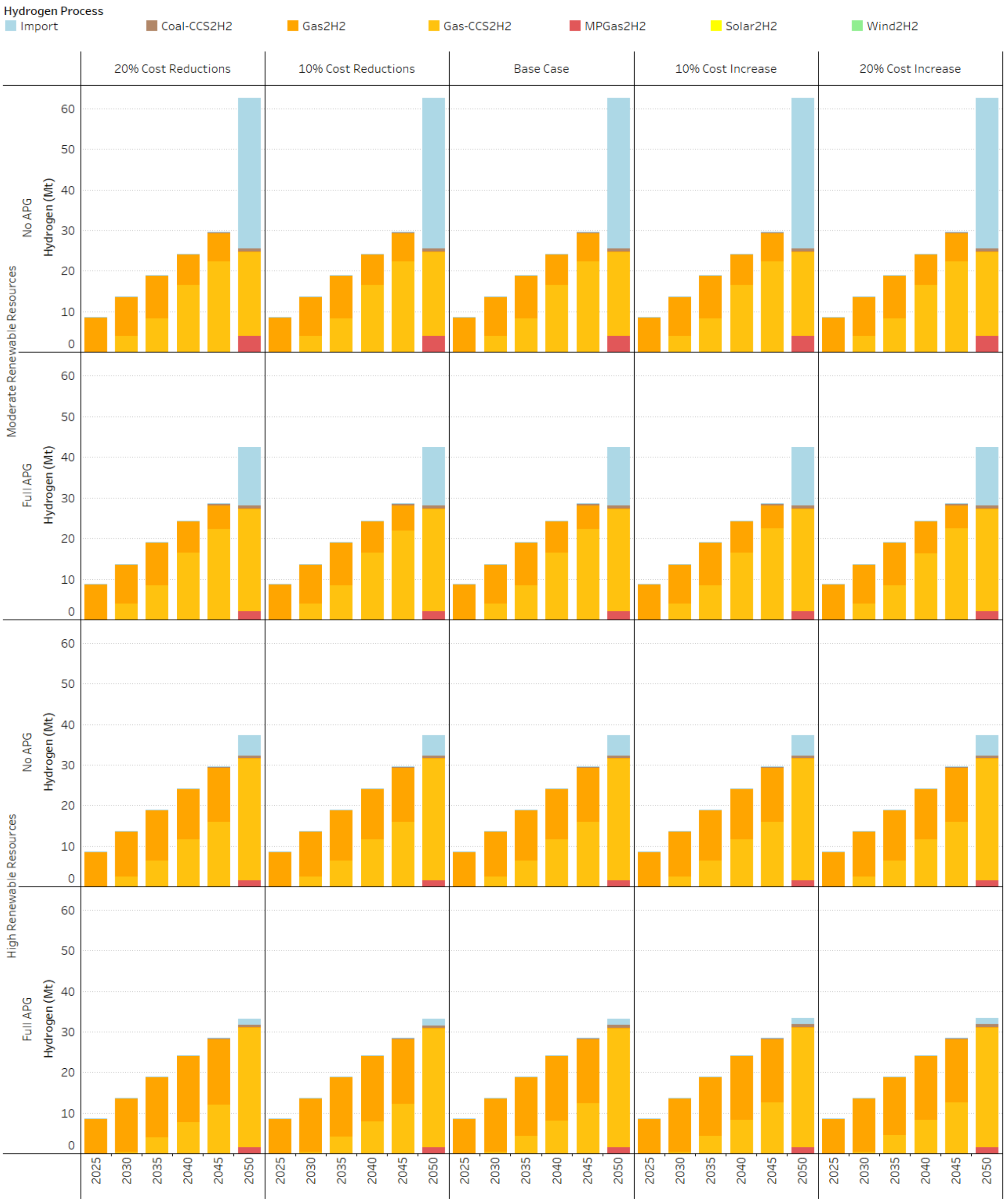

Figure S17. Effects of changing transmission cost on electricity generation and hydrogen production

11.9 Sensitivity tests of hydrogen demand

In this section we apply a 20% reduction, a 10% reduction, a 10% increase, and a 20% increase in the hydrogen demand. Figure S18 presents the sensitivity test results.

As shown in Panel b of Figure S18, the key technological choices for hydrogen production remain unchanged (i.e., gas with CCS and hydrogen import from outside ASEAN). Rising demand for hydrogen is met by increasing shares of gas with CCS and hydrogen import. Note that current model does not consider the hydrogen storage and transport between ASEAN countries. Rising hydrogen demand does

not lead to a higher utilization of renewable resources (e.g., VRE) in ASEAN for hydrogen production. Alternatively, low-carbon hydrogen import from Australia can play an important role (e.g., No APG and with moderate renewable resource potentials).

The patterns in electricity generation across all sensitivity tests are consistent (Panel a of Figure S18). An expansion in gas with CCS for hydrogen production may take place, due to rising hydrogen demand. With moderate renewable resource potentials, there would be only a marginal increase in biomass with CCS (to offset increasing emissions from hydrogen production) and hydrogen-to-electricity. Similarly, if high renewable potentials are available, the share of biomass with CCS in generation mix would increase (or decrease) due to the expansion (or reduction) in gas with CCS for hydrogen production. But such changes in generation mix are only marginal (less than 1%).

(a) Effects of changing hydrogen demand on electricity generation

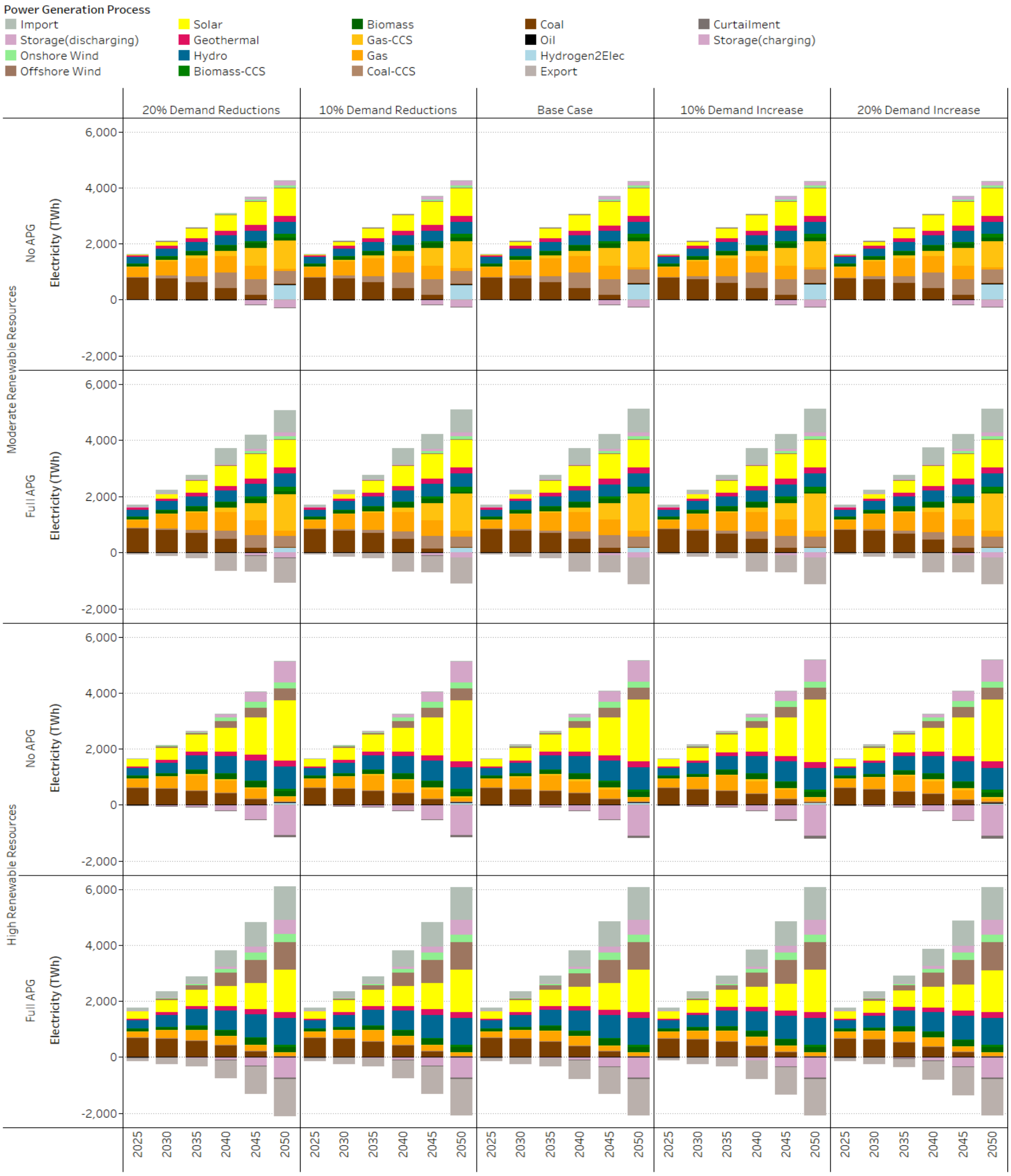

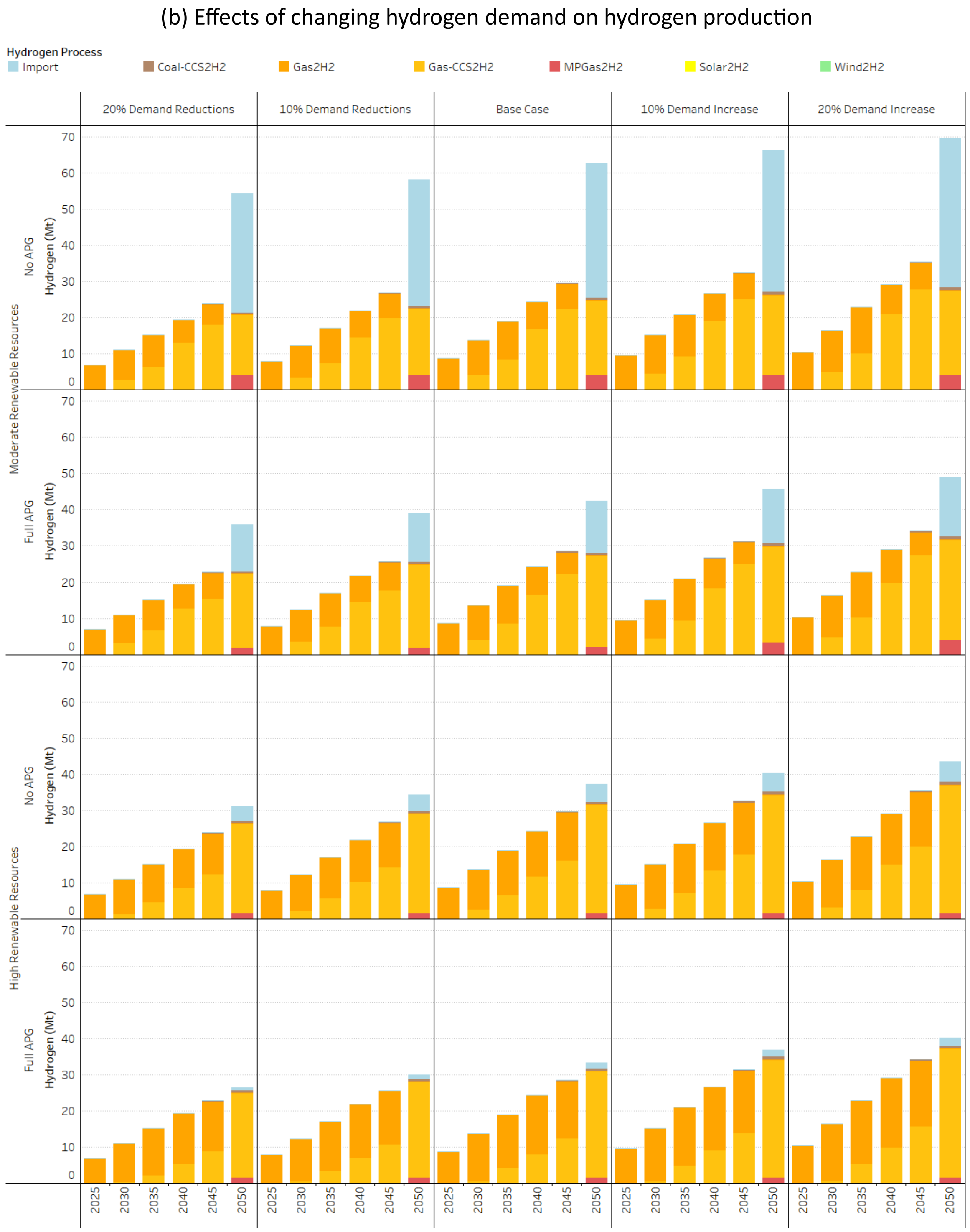

Figure S18. Effects of changing hydrogen demand on electricity generation and hydrogen production

References

[1] Dorfner J. urbs: A Linear Optimisation Model for Distributed Energy Systems. 2023.
[2] Zhong S, Yang L, Papageorgiou DJ, Su B, Ng TS, Abubakar S. Accelerating ASEAN's Energy Transition in the Power Sector through Cross-Border Transmission and a Net-Zero 2050 View. iScience. 2025;28(1).
[3] Cole W, Eurek K, Vincent N, Mai T, Mowers M, Brinkman G. Operating Reserves in Long-term Planning Models. NREL/PR-6A20-71148 ed: NREL; 2018.
[4] IEA. IEA World Energy Statistics and Balances. Paris: IEA; 2023.
[5] IEA. Southeast Asia Energy Outlook 2022. Paris: IEA; 2022.
[6] IRENA. Renewable Energy Outlook for ASEAN: Towards a Regional Energy Transition (2nd Edition). Abu Dhabi: IRENA; 2022.
[7] IEA. World Energy Outlook 2022. Paris: IEA; 2022.
[8] IEA. World Energy Outlook 2023. Paris: IEA; 2023.
[9] NCCS. Study of Hydrogen Imports and Downstream Applications for Singapore. Singapore: National Climate Change Secretariat; 2021.
[10] EMA. Half-hourly System Demand Data. Singapore: Energy Market Authority of Singapore (EMA); 2023.
[11] NGCP. Hourly Demand. Manila: National Grid Corporation of the Philippines (NGCP). 2023.
[12] PEA. Load Research of PEA. Provincial Electricity Authority of Thailand (PEA). 2023.
[13] Handayani K, Anugrah P, Goembira F, Overland I, Suryadi B, Swandaru A. Moving beyond the NDCs: ASEAN Pathways to a Net-Zero Emissions Power Sector in 2050. Appl Energy. 2022;311:118580.
[14] NREL. Annual Technology Baseline. Golden, CO: NREL; 2021.
[15] Tsiropoulos I, Tarvydas D, Zucker A. Cost Development of Low Carbon Energy Technologies: Scenario-based Cost Trajectories to 2050: Scenario-based Cost Trajectories to 2050, 2017 Edition. Luxembourg: Publications Office of the European Union, 2018.
[16] IEA. CCUS in Clean Energy Transitions. Paris: IEA; 2020.
[17] Goh T, Ang BW, Su B, Wang H. Drivers of Stagnating Global Carbon Intensity of Electricity and the Way Forward. Energy Policy. 2018;113:149-56.
[18] ACE. The 7th ASEAN Energy Outlook. Jakarta: ACE; 2022.
[19] IEA. Projected Costs of Generating Electricity 2020. Paris: IEA; 2020.
[20] Enerdata. Power Plant Tracker. 2021.
[21] ACE. Spatial Estimate of Levelised Costs of Electricity (LCOE) in ASEAN. Jakarta: ASEAN Centre for Energy; 2019.
[22] NREL. RE Explorer. Golden, CO: NREL; 2022.
[23] MEMR, Danish Energy Agency, Ea Energy Analysis. Technology Data for the Indonesian Power Sector-Catalogue for Generation and Storage of Electricity. Danish Energy Agency; 2021.
[24] IEA. World Energy Outlook 2019. Paris: IEA; 2019.
[25] Huber M, Roger A, Hamacher T. Optimizing Long-Term Investments for a Sustainable Development of the ASEAN Power System. Energy. 2015;88:180-93.
[26] Sanchez DL, Nelson JH, Johnston J, Mileva A, Kammen DM. Biomass Enables the Transition to a Carbon-Negative Power System across Western North America. Nat Clim Chang. 2015;5(3):230-4.
[27] Stich J, Massier T. Enhancing the Integration of Renewables by Trans-Border Electricity Trade in ASEAN. 2015 IEEE Pes Asia-Pacific Power and Energy Engineering Conference. New York: IEEE; 2015.
[28] IEA. CO2 Emissions from Fuel Combustion Statistics. Paris: IEA,; 2021.
[29] Kerscher F, Stary A, Gleis S, Ulrich A, Klein H, Spliethoff H. Low-Carbon Hydrogen Production via Electron Beam Plasma Methane Pyrolysis: Techno-Economic Analysis and Carbon Footprint Assessment. Int J Hydrog Energy. 2021;46(38):19897-912.
[30] ACE. Hydrogen in ASEAN: Economic Prospects, Development, and Applications. Jakarta: ASEAN Centre for Energy; 2021.

[31] Liszka M, Malik T, Manfrida G. Energy and Exergy Analysis of Hydrogen-Oriented Coal Gasification with CO2 Capture. Energy. 2012;45(1):142-50.
[32] Hermesmann M, Müller TE. Green, Turquoise, Blue, or Grey? Environmentally friendly Hydrogen Production in Transforming Energy Systems. Progress in Energy and Combustion Science. 2022;90:100996.
[33] Blank TK, Molloy P. Hydrogen's Decarbonization Impact for Industry: Near-Term Challenges and Long-Term Potential. Rocky Mountain Institute; 2020.
[34] IRENA. Statistical Profiles. Abu Dhabi: IRENA; 2021.
[35] Government of Indonesia. Indonesia Second Biennial Update Report. UNFCCC; 2018.
[36] Paltsev S, Mehling M, Winchester N, Morris J, Ledvina K. Pathways to Paris: ASEAN. MIT Joint Program Special Report. Boston: MIT; 2018.
[37] Asian Development Bank. Cambodia: Energy Sector Assessment, Strategy, and Road Map. Manila: Asian Development Bank, 2018.
[38] Government of Myanmar. Nationally Determined Contributions. UNFCCC; 2021.
[39] Asian Development Bank. Lao People's Democratic Republic: Energy Sector Assessment, Strategy, and Road Map. Manila: Asian Development Bank, 2019.
[40] DOE of the Philippines. Philippines Energy Plan 2020-2040. Manila: Department of Energy (DOE), 2021.
[41] NCCS. Update of the Solar Photovoltaic (PV) Roadmap for Singapore. Singapore: NCCS; 2020.
[42] Baker & McKenzie. Vietnam: Key Highlights of New Draft of National Power Development Plan (Draft PDP8). Baker & McKenzie; 2021.
[43] Ministry of Economy of Malaysia. National Energy Transition Roadmap. Ministry of Economy of Malaysia; 2023.
[44] Asian Development Bank. Philippines: Energy Sector Assessment, Strategy, and Road Map. Manila: Asian Development Bank, 2018.
[45] IRENA. Renewable Energy Outlook: Thailand. Abu Dhabi: IRENA, 2017.
[46] Tun MM, Juchelkova D, Win MM, Thu AM, Puchor T. Biomass Energy: An Overview of Biomass Sources, Energy Potential, and Management in Southeast Asian Countries. Resources. 2019;8(2).
[47] IRENA. Renewables Readiness Assessment: The Philippines. Abu Dhabi: IRENA, 2017.
[48] IEA. Southeast Asia Energy Outlook 2019. Paris: IEA; 2019.
[49] IEA. Establishing multilateral power trade in ASEAN. Paris: IEA; 2019.
[50] EMA. Charting the Energy Transition to 2050: Energy 2050 Committee Report. Singapore: EMA; 2022.
[51] ERIA. Investing in Power Grid Interconnection in East Asia. Jakarta: ERIA; 2014.
[52] IEA. Energy Prices. Paris: IEA; 2021.
[53] EIA. Annual Energy Outlook 2023. Washington, D.C.: U.S. Energy Information Administration; 2023.
[54] World Bank. Commodity Markets. Washington D.C.: World Bank; 2023.
[55] OPIS. McCloskey by OPIS. OPIS; 2023.
[56] IEA. Global Energy and Climate Model. Paris: IEA; 2022.
[57] ACE. Levelised Costs of Electricity for Renewable Energy Technologies in ASEAN Member States II. Jakarta: ASEAN Centre for Energy; 2019.
[58] Paardekooper S, Lund RS, Mathiesen BV, Chang M, Petersen UR, Grundahl L, et al. EU28 Fuel Prices for 2015, 2030 and 2050. Heat Roadmap Europe 4: Quantifying the Impact of Low-Carbon Heating and Cooling Roadmaps2018.
[59] World Bank. Climate Change Knowledge Portal. Washington DC: World Bank; 2021.
[60] NASA. POWER data Access Viewer. NASA; 2021.
[61] Penn State University. Wind Energy and Power Calculations. Penn State College of Earth and Mineral Sciences; 2023.
[62] U.S. Department of Energy. Wind Turbines: the Bigger, the Better. Washington DC: Office of Energy Efficiency & Renewable Energy; 2022.

[63] Pfenninger S, Staffell I. Renewables.ninja. 2024.
[64] IEA. Offshore Wind Outlook. Paris: IEA; 2019.
[65] European Commission. PVGIS Photovoltaic Geographical Information System. Joint Research Centre; 2022.
